# Supplementary material for: CLDN1 Sensitizes Triple-Negative Breast Cancer Cells to Chemotherapy
Source: Cancers (Basel). 2022 Oct 14;14(20):5026. doi: 10.3390/cancers14205026 (PMC9599637; doi:10.3390/cancers14205026)

Membrane management

All nitrocellulose membranes are cut after transfer to be hybridized with different antibodies. The membranes are cut into 3 pieces:

- from 0 to 30 kDa
- from 30 to 75 kDa
- from 75 to 250 kDa

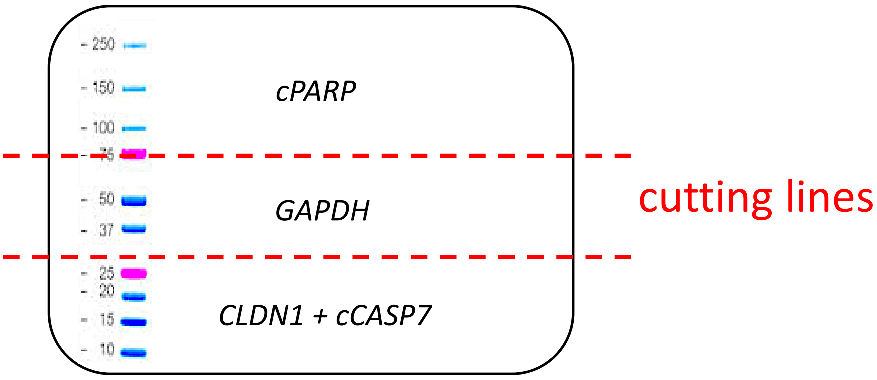

The 0 to 30 kDa membrane pieces are hybridized with the ani-CLDN1 or anti-cCASP7 antibody.
The 30 to 75 kDa pieces are hybridized with the anti-GAPDH antibody.
The 75 to 250 kDa pieces are hybridized with the anti-cPARP antibody.

Membranes are revealed using the peroxidase system in Chemidocs plus. Several membranes are placed in the device at the same time.

FIGURE 2D HCC1806 5-FU n°1

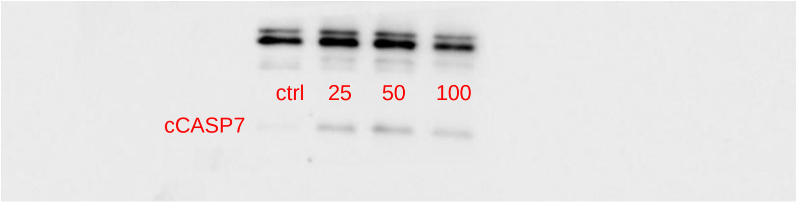

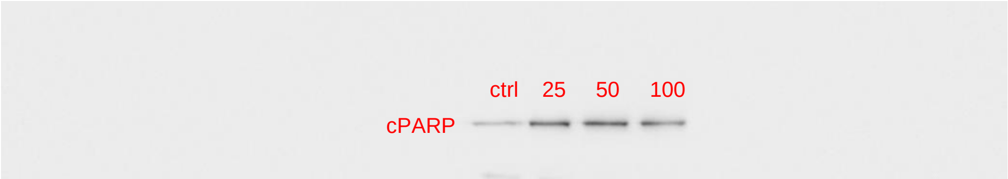

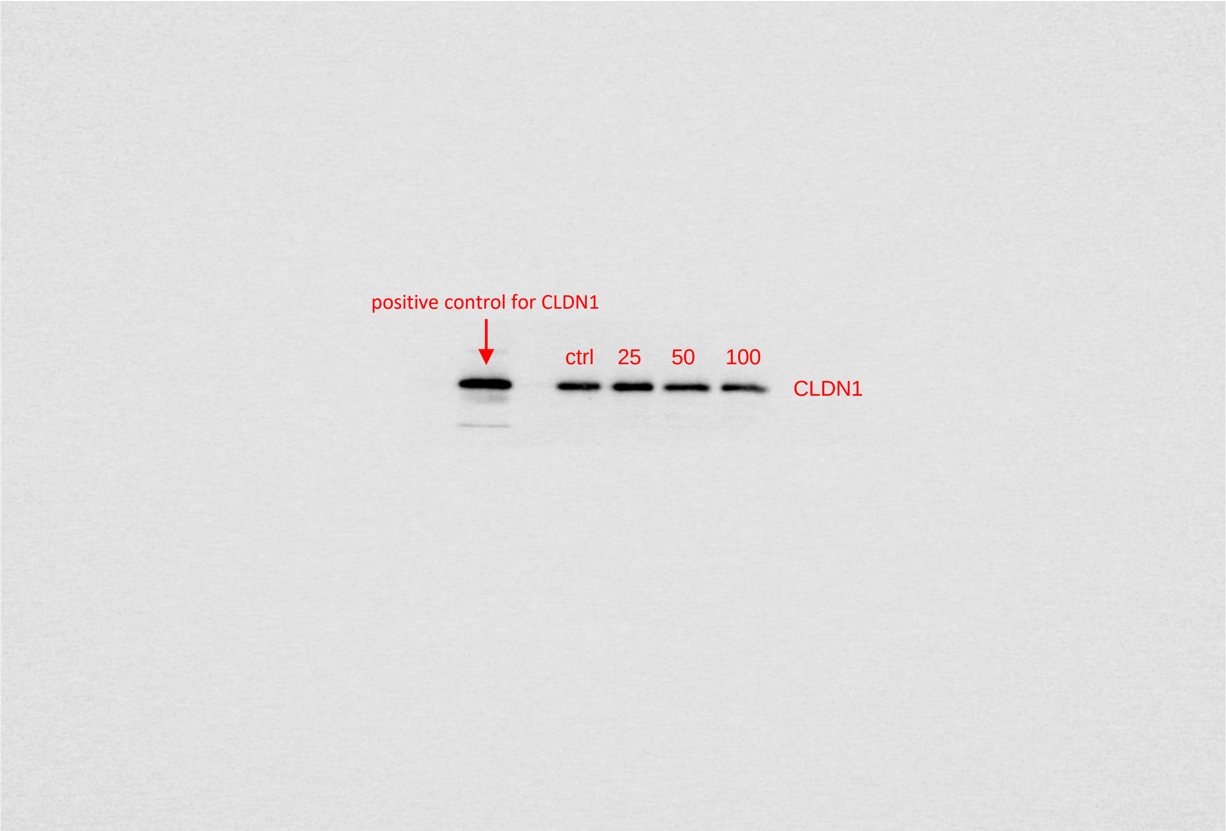

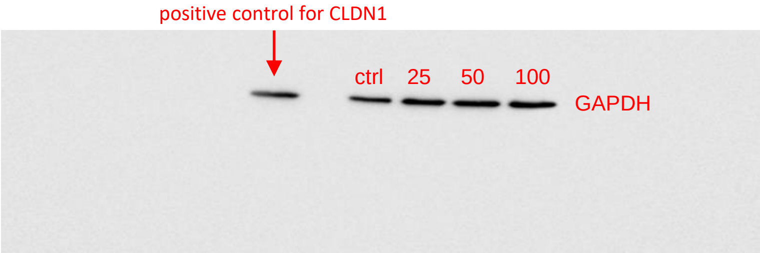

FIGURE 2D HCC1806 5-FU n°2

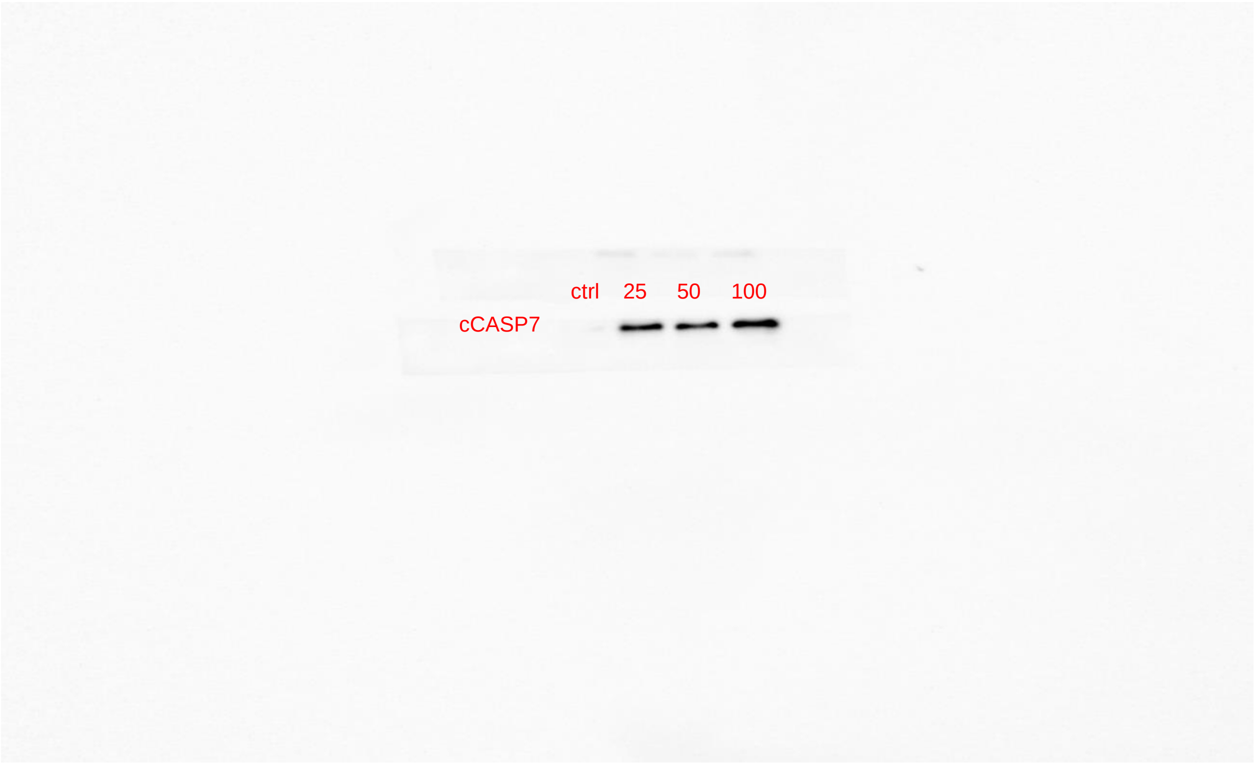

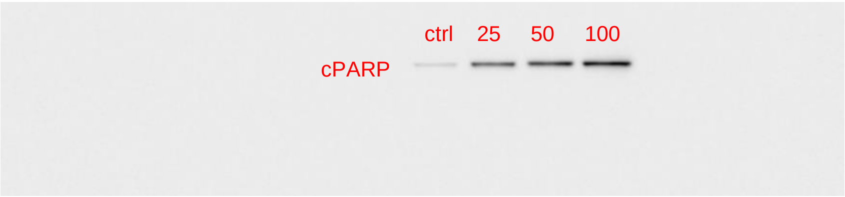

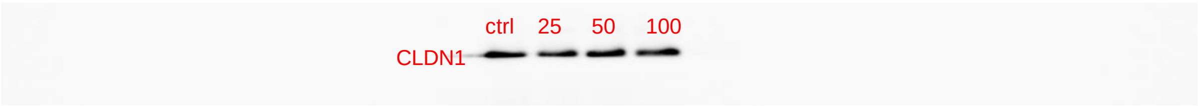

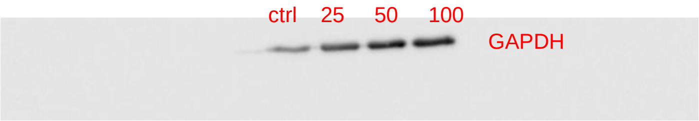

FIGURE 2D HCC1806 5-FU n°3

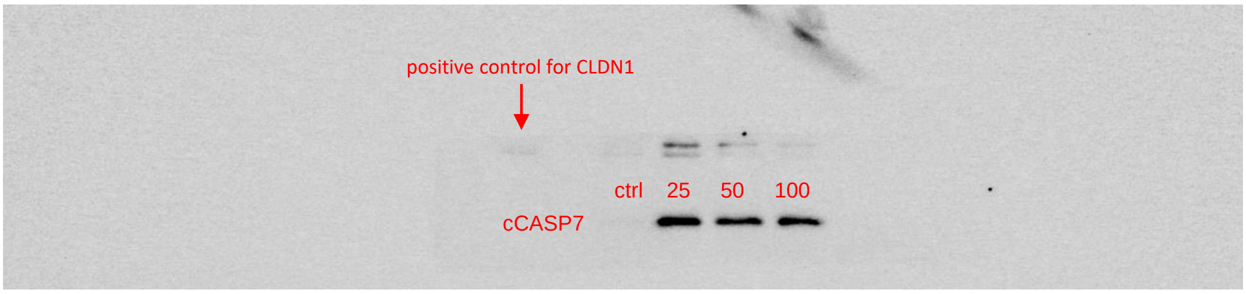

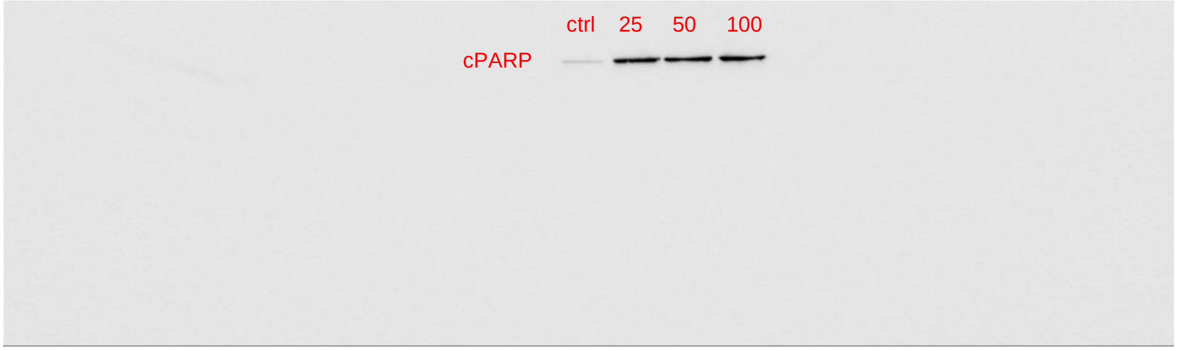

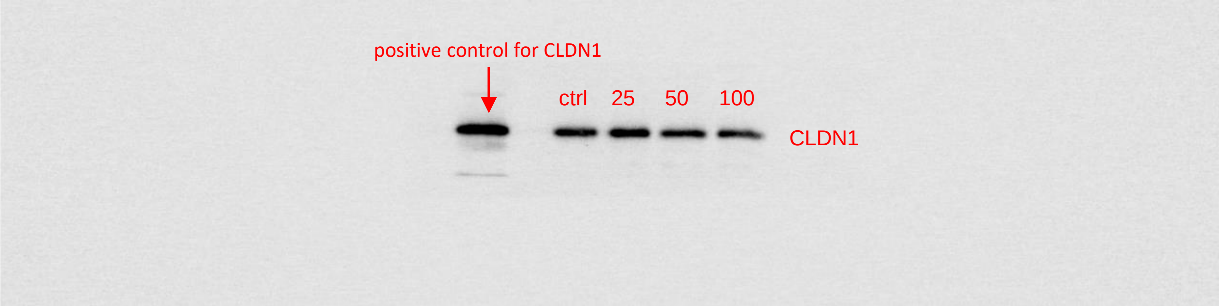

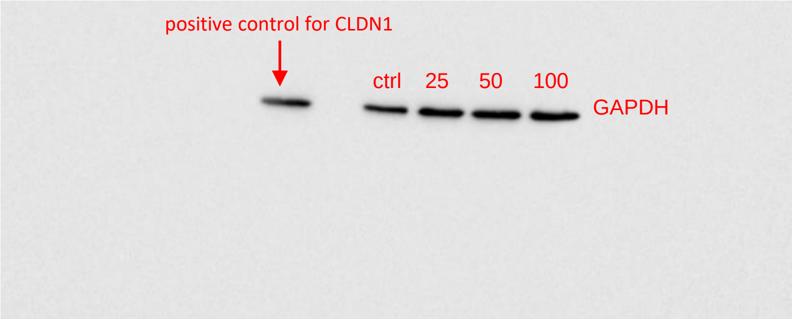

FIGURE 2D HCC1806 5-FU n°4

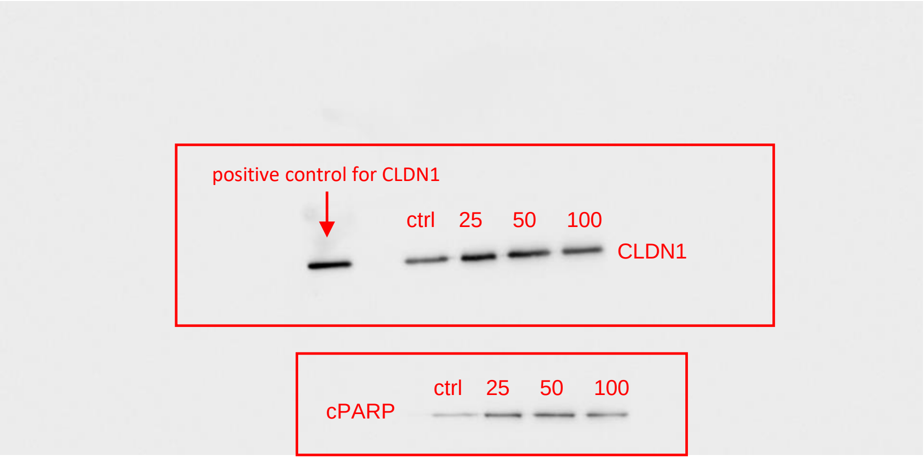

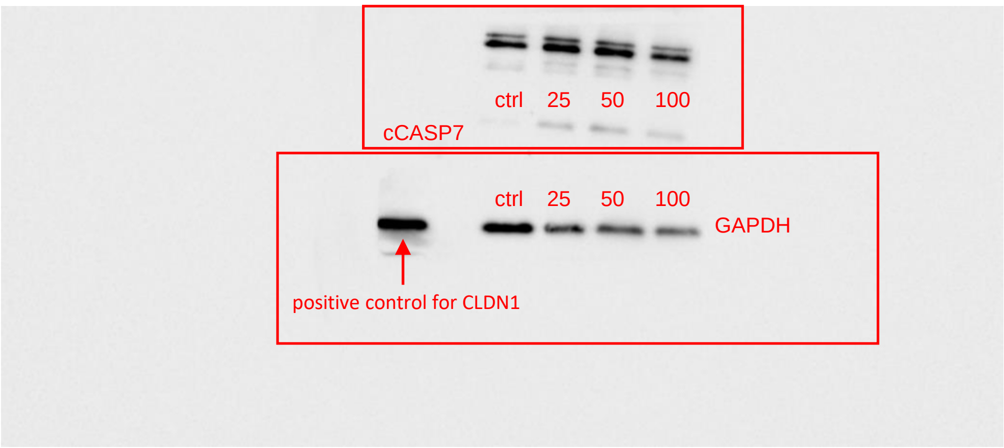

FIGURE 2D MDA-MB-231 5-FU n°1

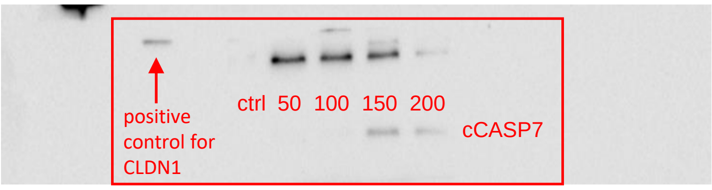

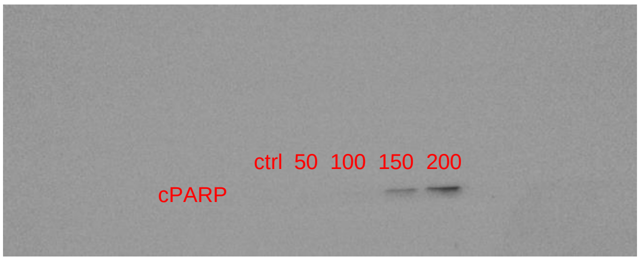

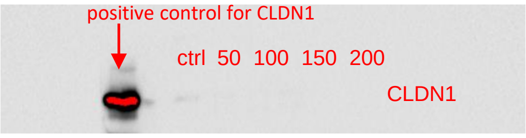

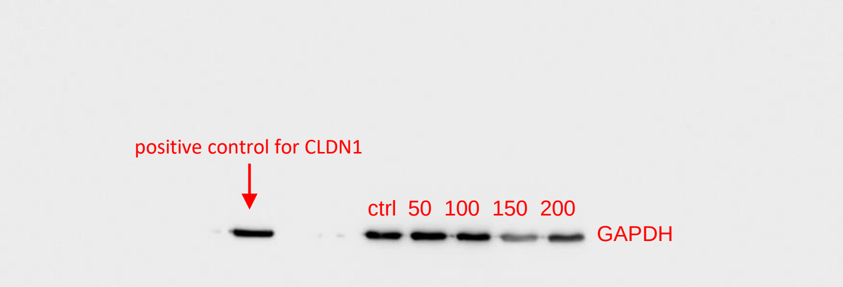

FIGURE 2D MDA-MB-231 5-FU n°2

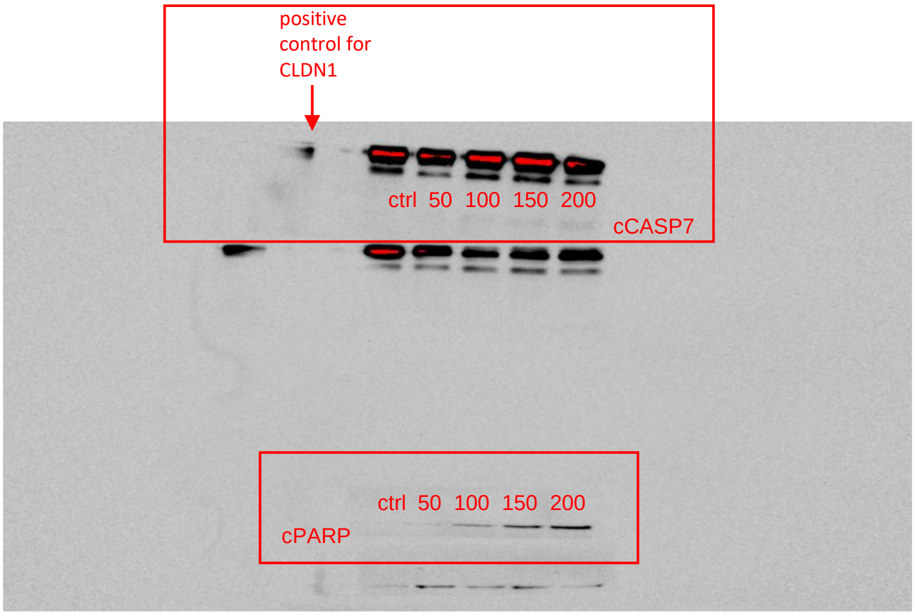

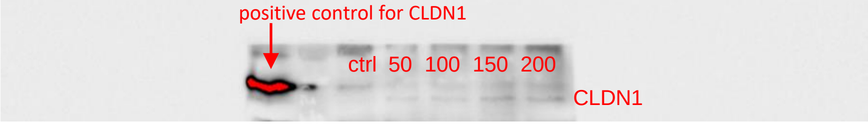

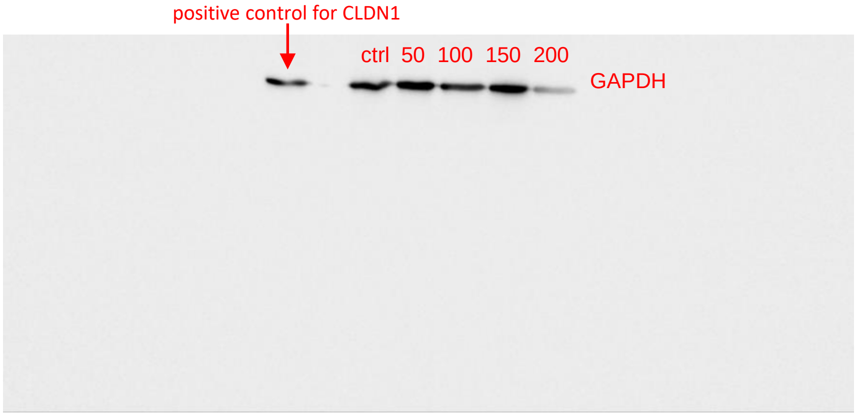

FIGURE 2D MDA-MB-231 5-FU n°3

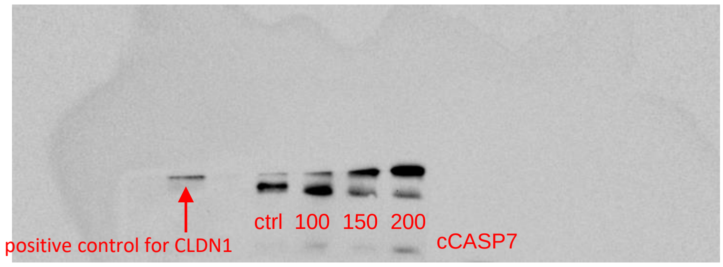

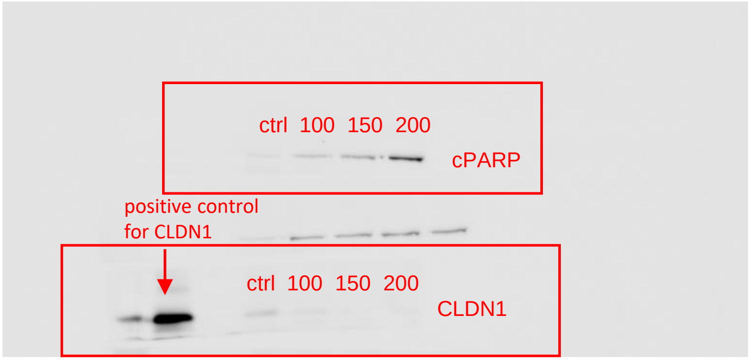

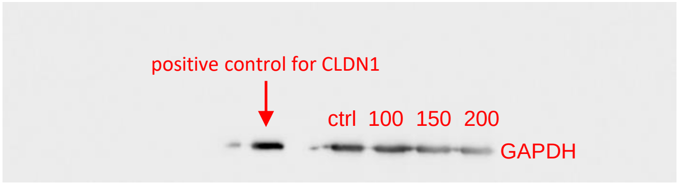

FIGURE 2D MDA-MB-231 5-FU n°4

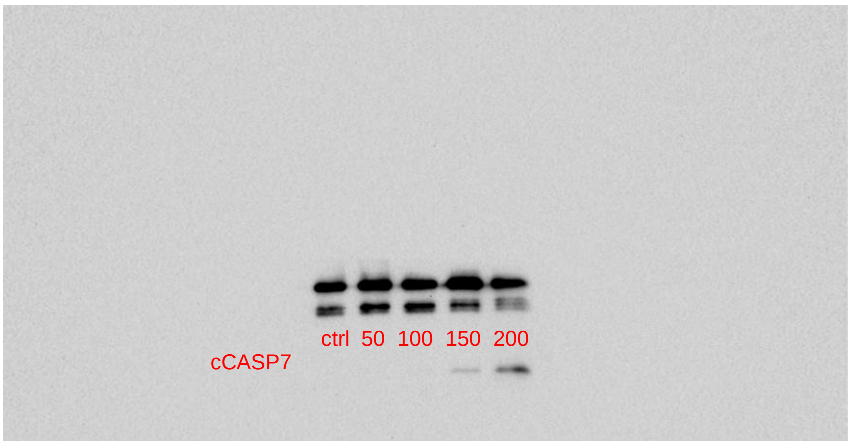

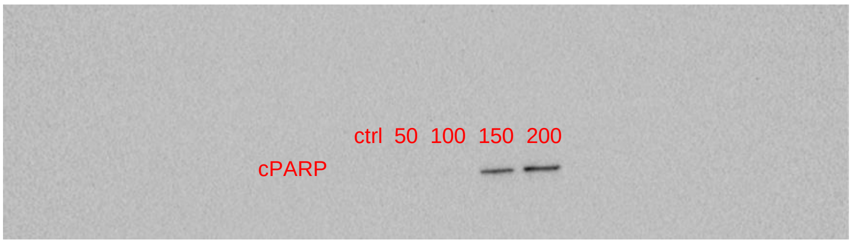

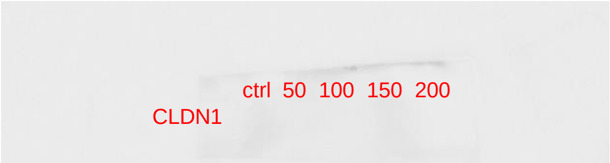

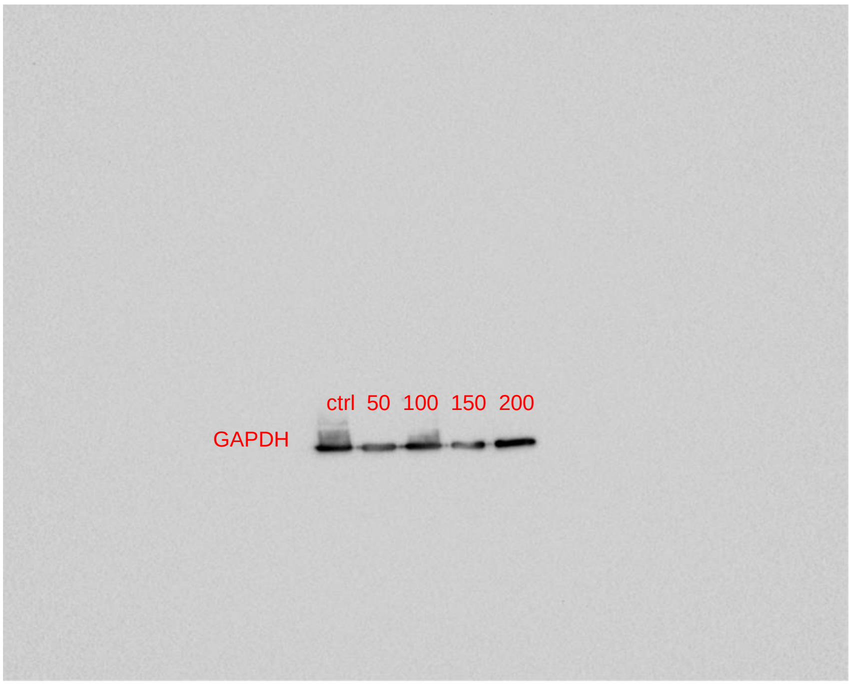

FIGURE 2D Hs578T 5-FU n°1

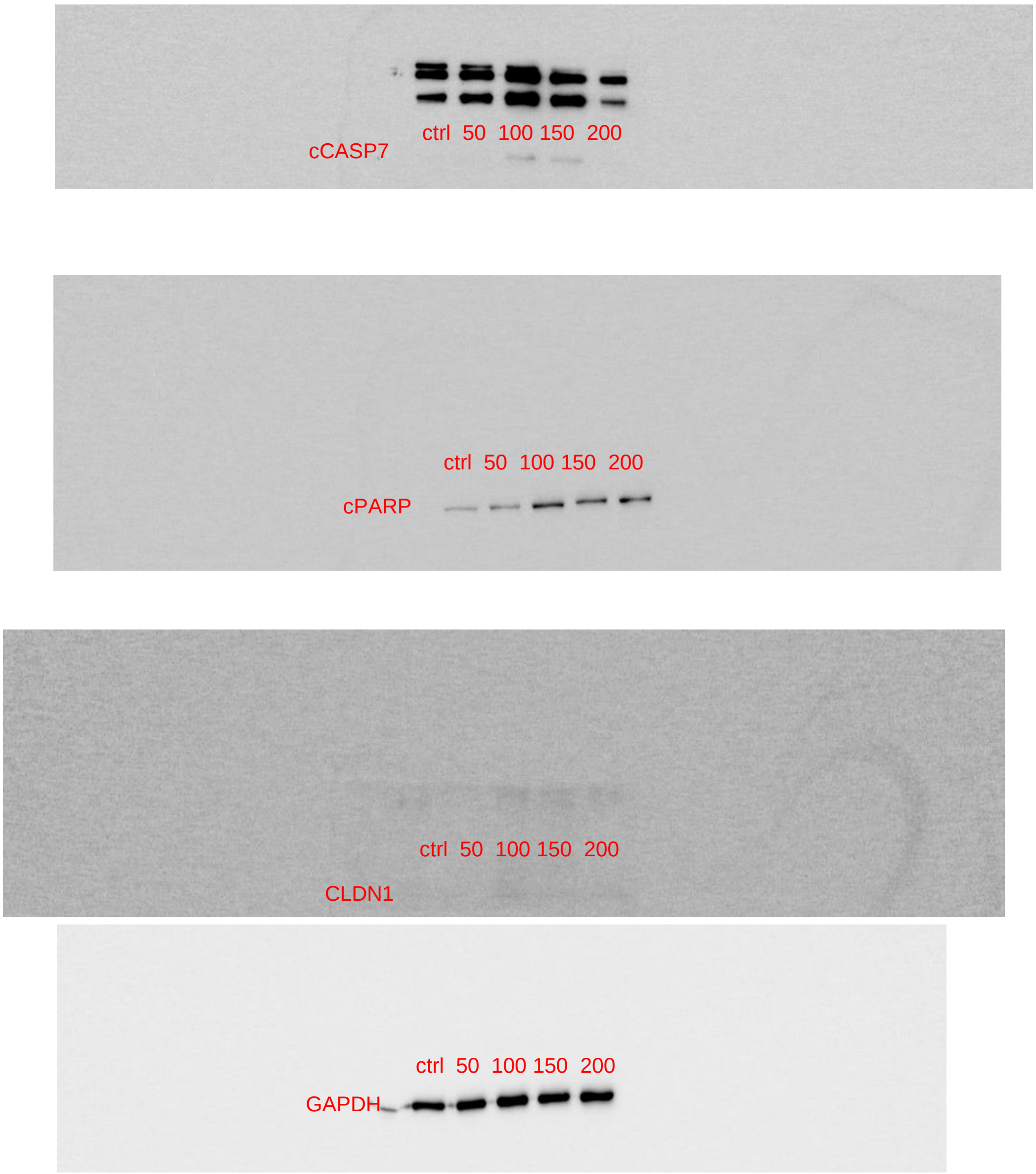

FIGURE 2D Hs578T 5-FU n°2

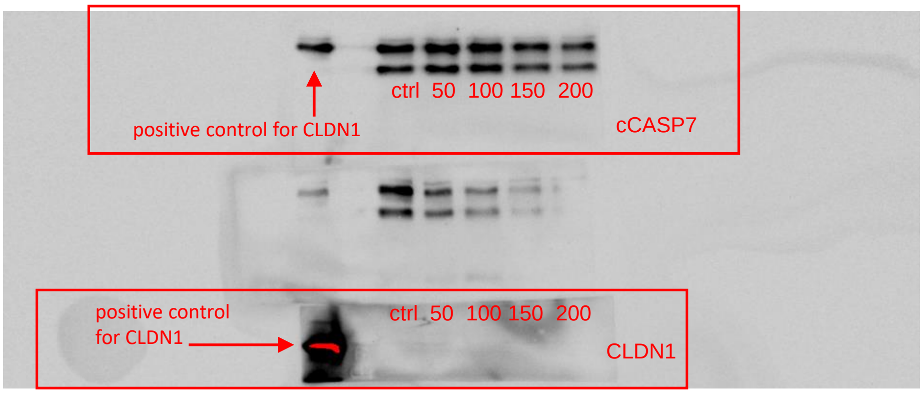

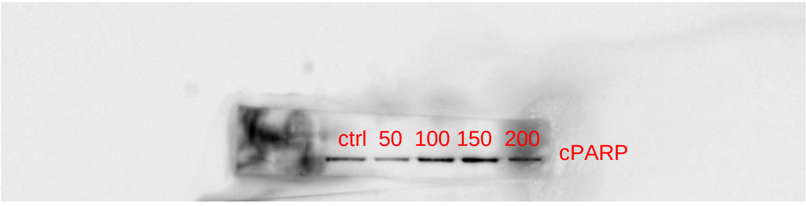

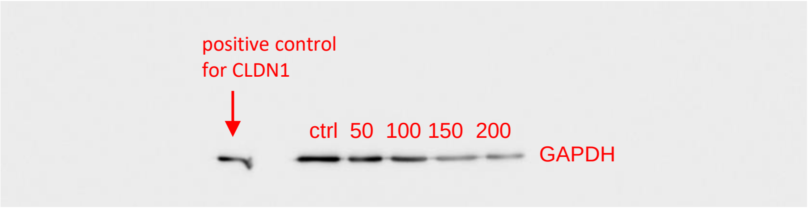

FIGURE 2D Hs578T 5-FU n°3

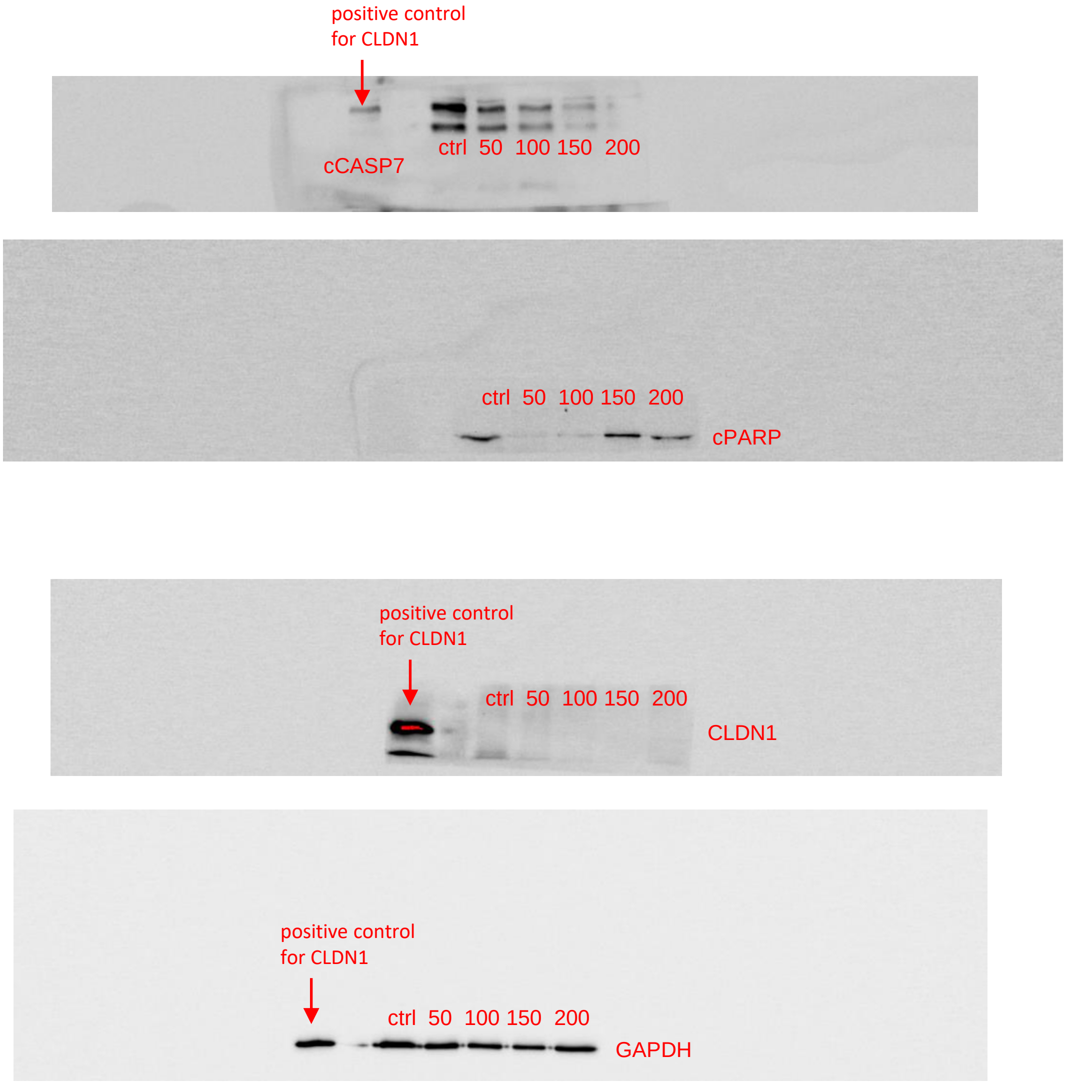

FIGURE 2D Hs578T 5-FU n°4

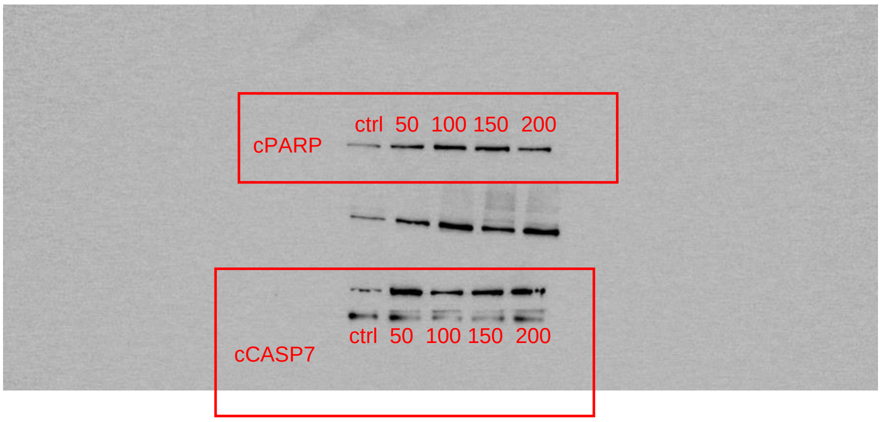

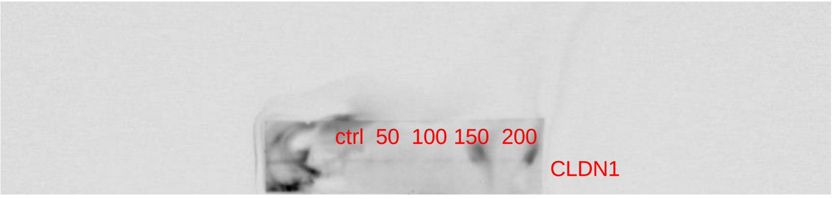

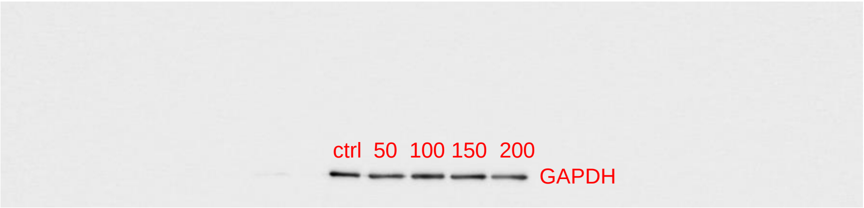

FIGURE 2E HCC1806 PTX n°1

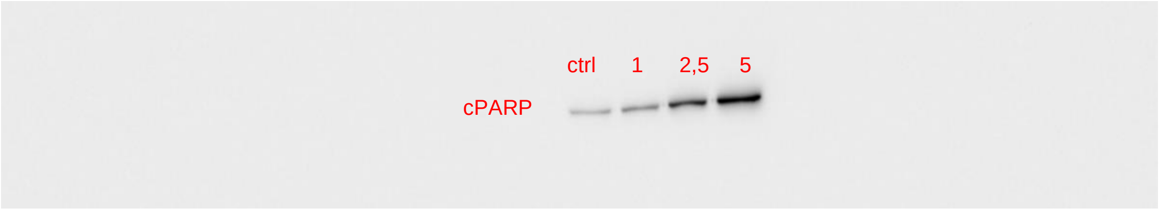

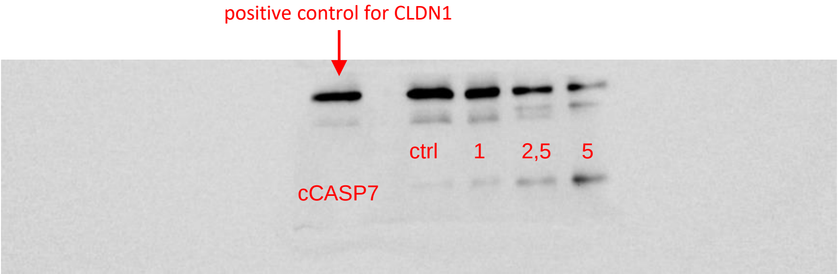

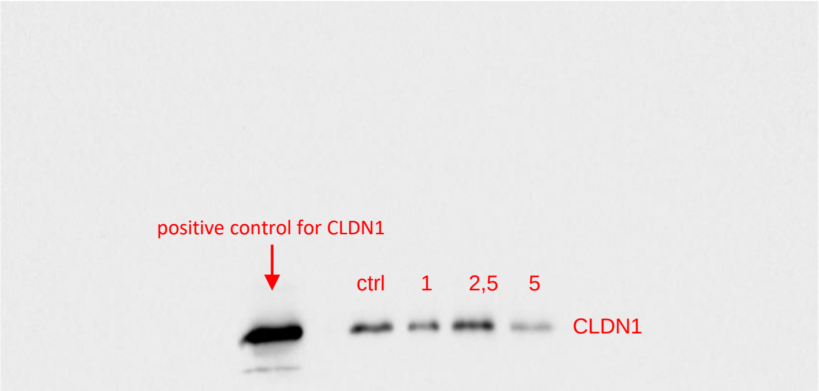

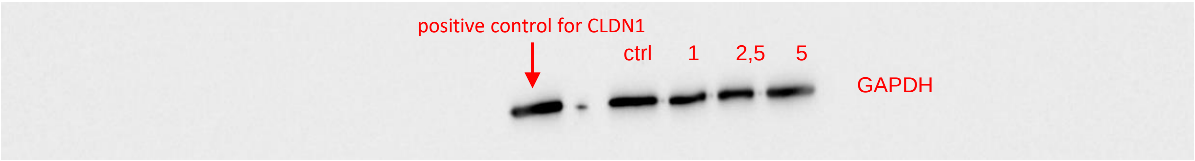

FIGURE 2E HCC1806 PTX n°2

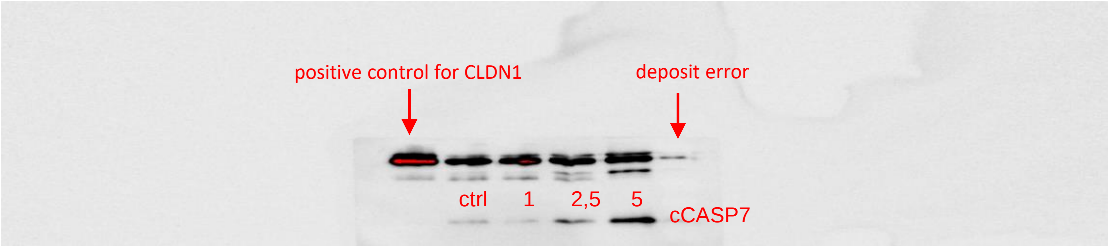

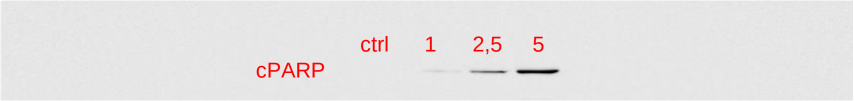

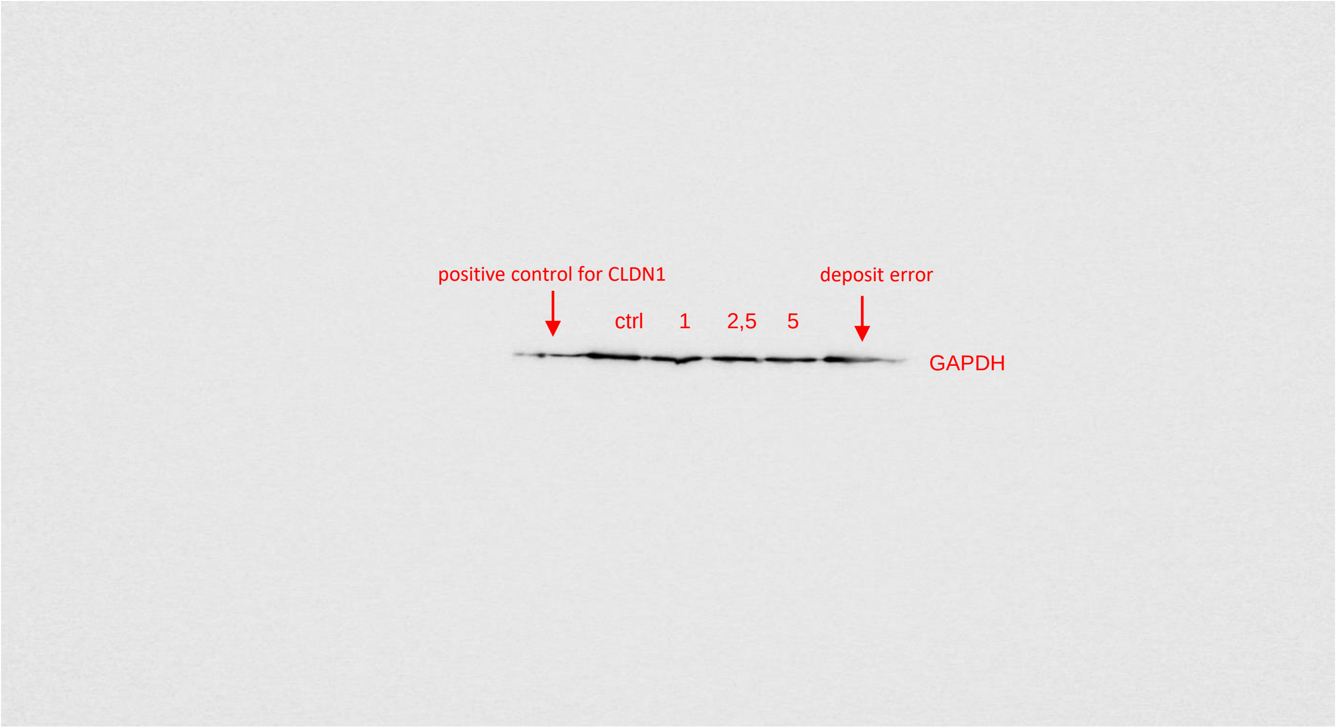

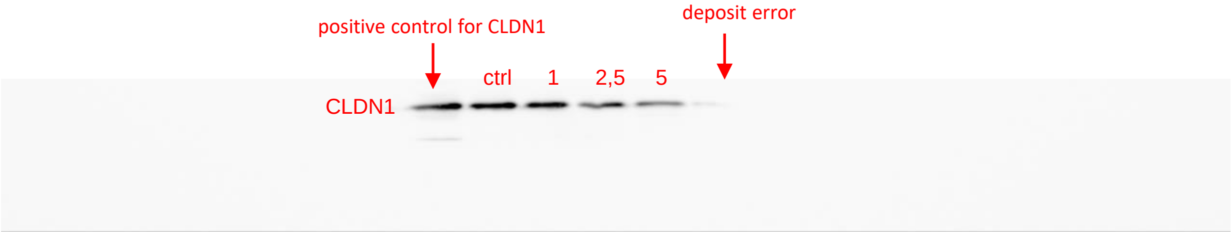

FIGURE 2E HCC1806 PTX n°3

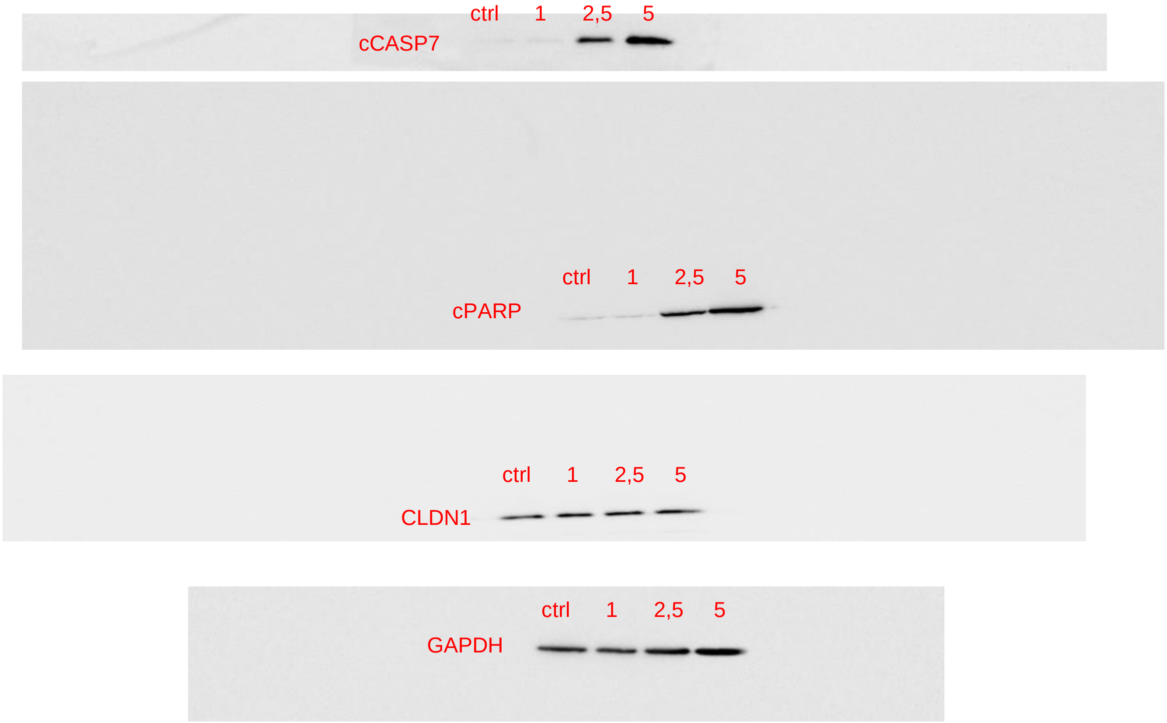

FIGURE 2E HCC1806 PTX n°4

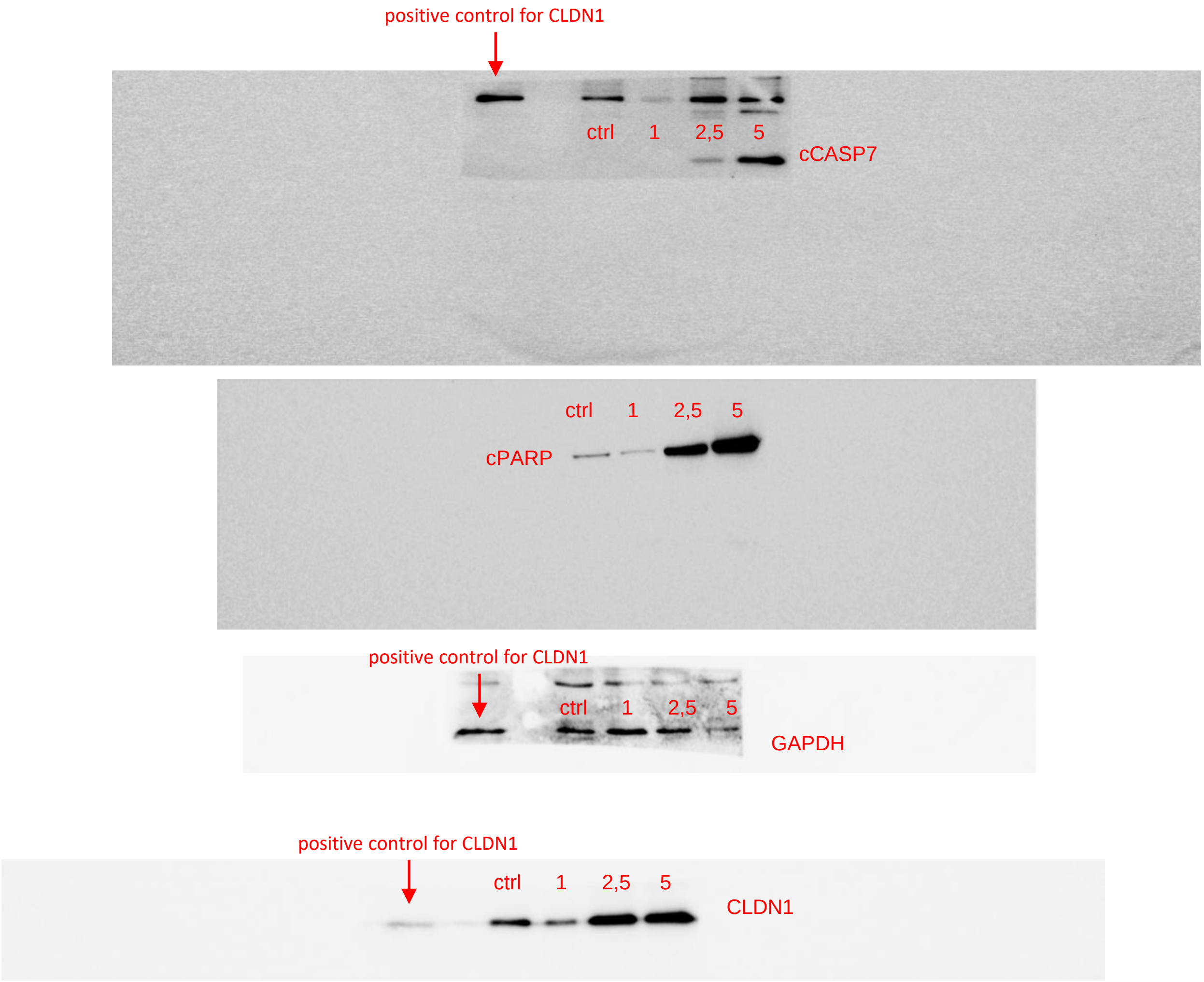

FIGURE 2E MDA-MB-231 PTX n°1

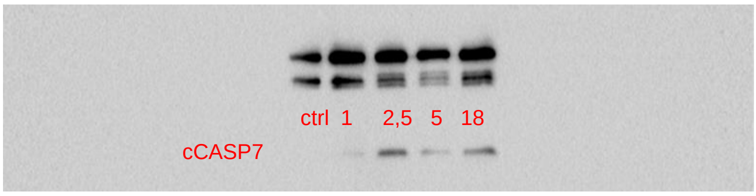

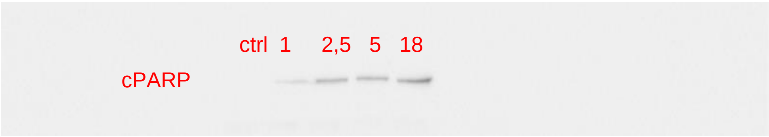

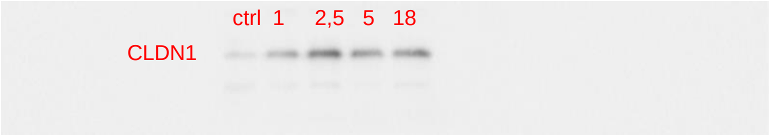

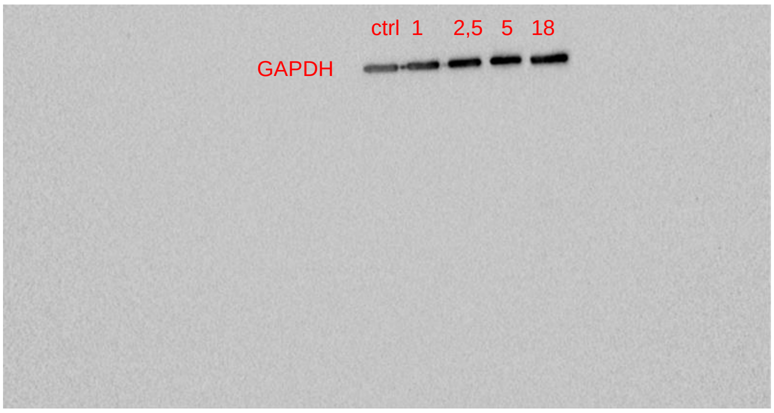

FIGURE 2E MDA-MB-231 PTX n°2

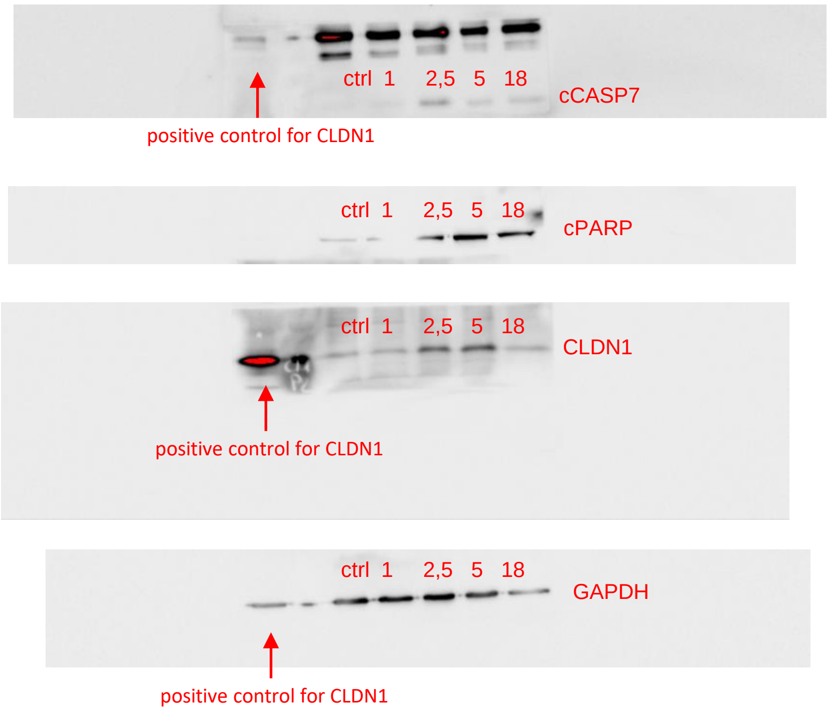

FIGURE 2E MDA-MB-231 PTX n°3

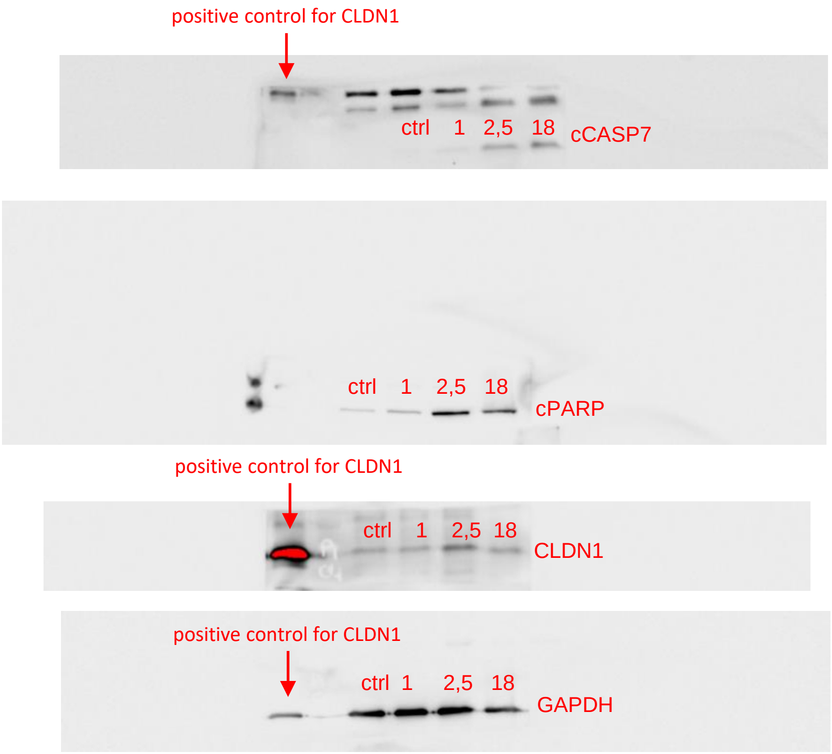

FIGURE 2E Hs578T PTX n°1

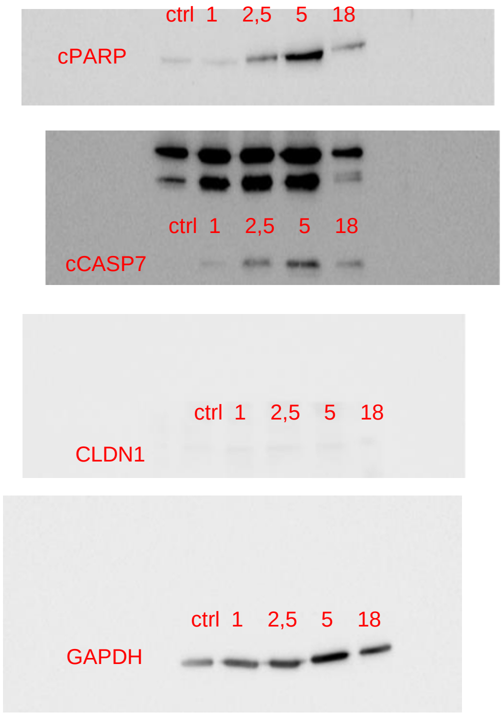

FIGURE 2E Hs578T PTX n°2

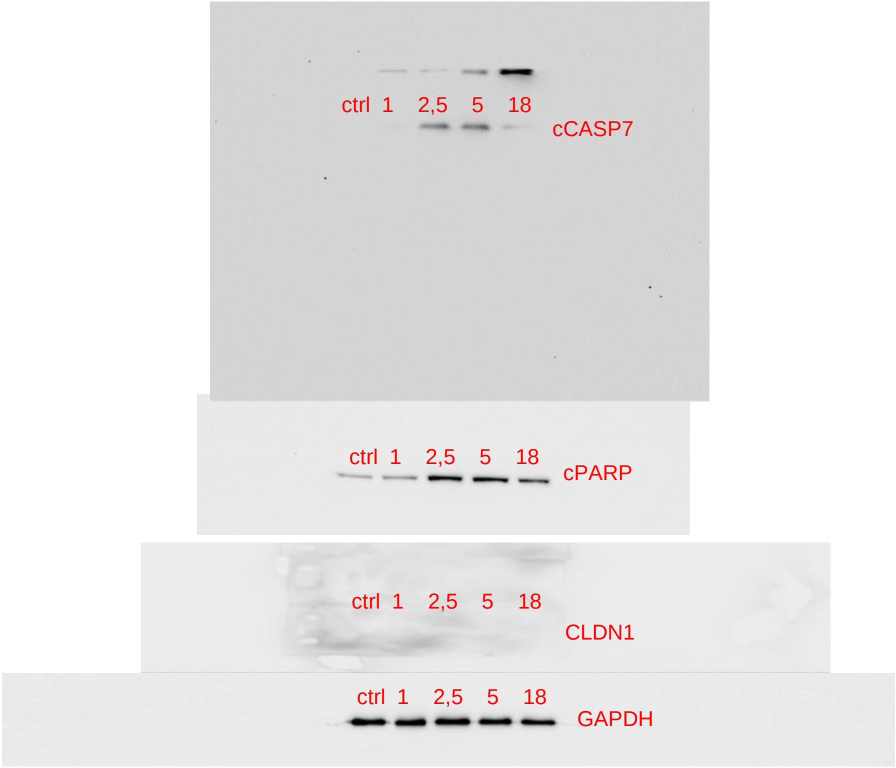

FIGURE 2E Hs578T PTX n°3

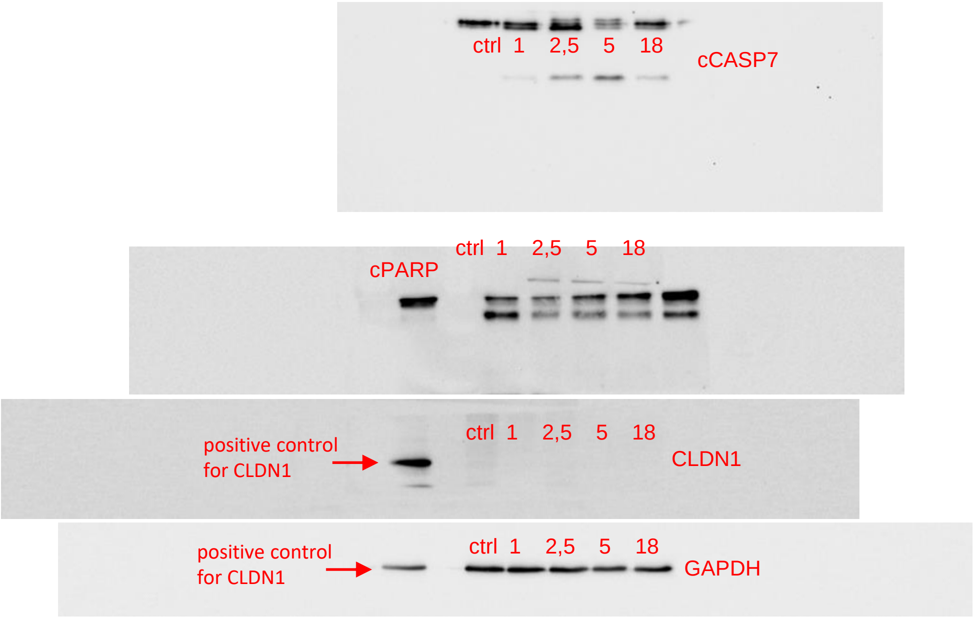

FIGURE 2F HCC1806 DOX n°1

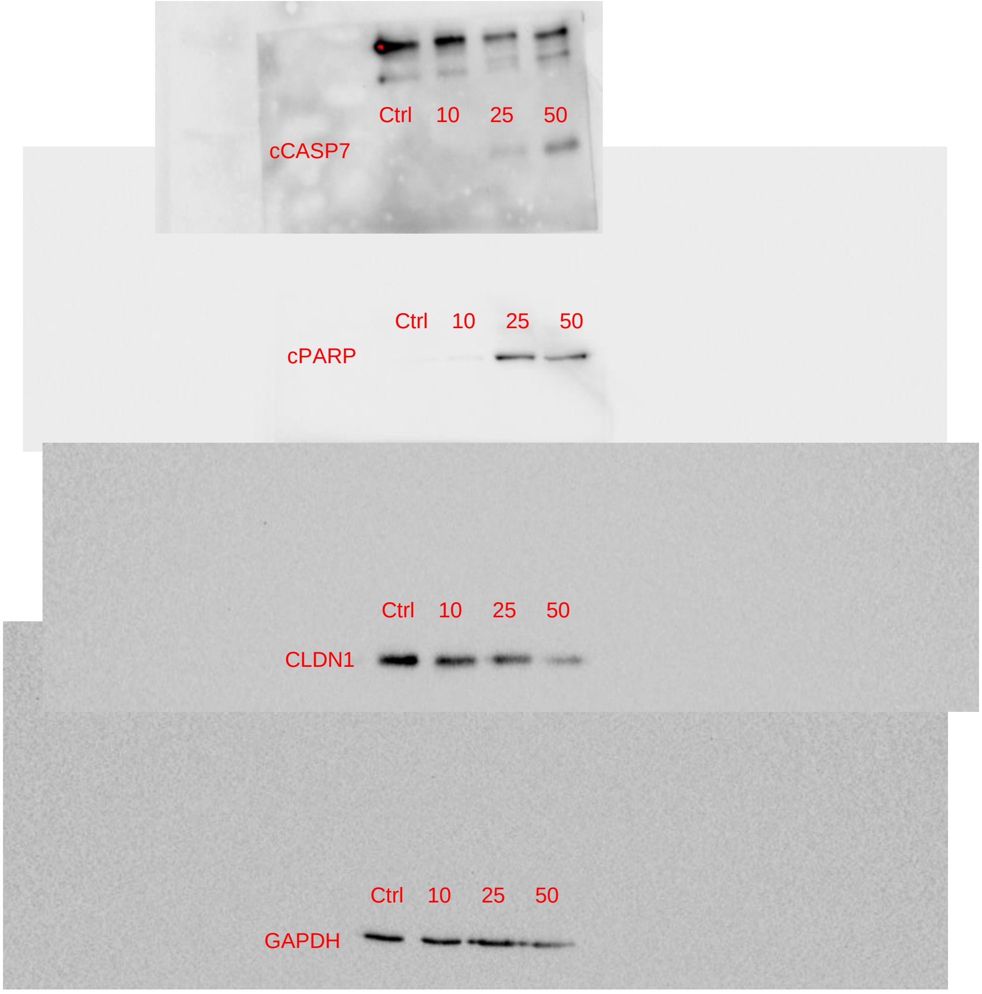

FIGURE 2F HCC1806 DOX n°2

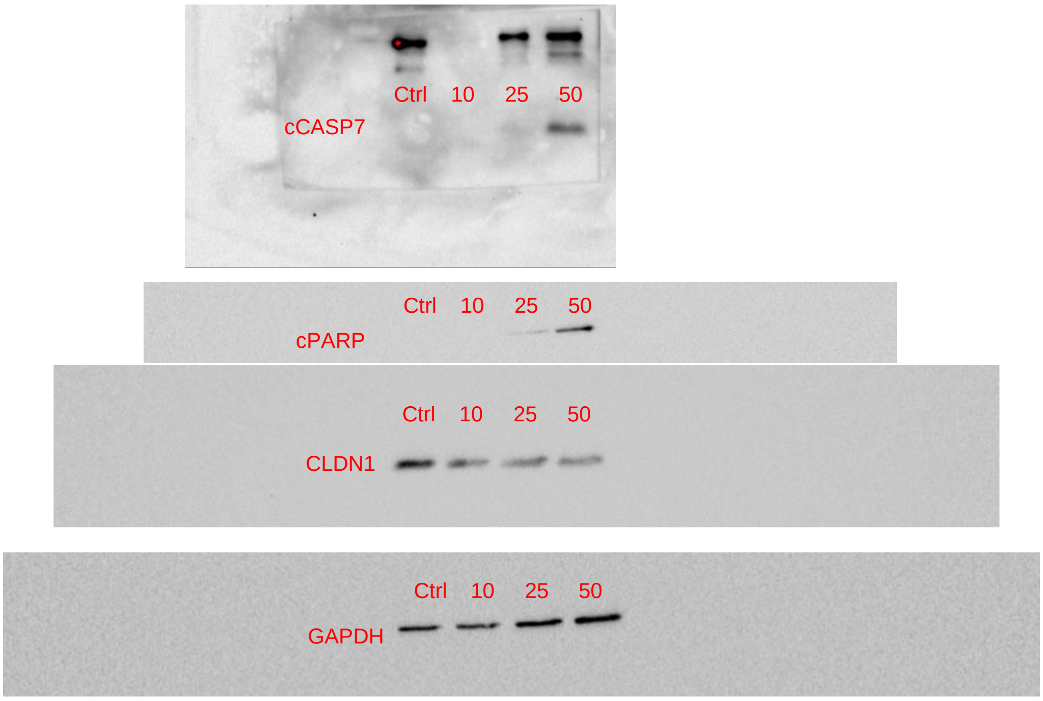

FIGURE 2F HCC1806 DOX n°3

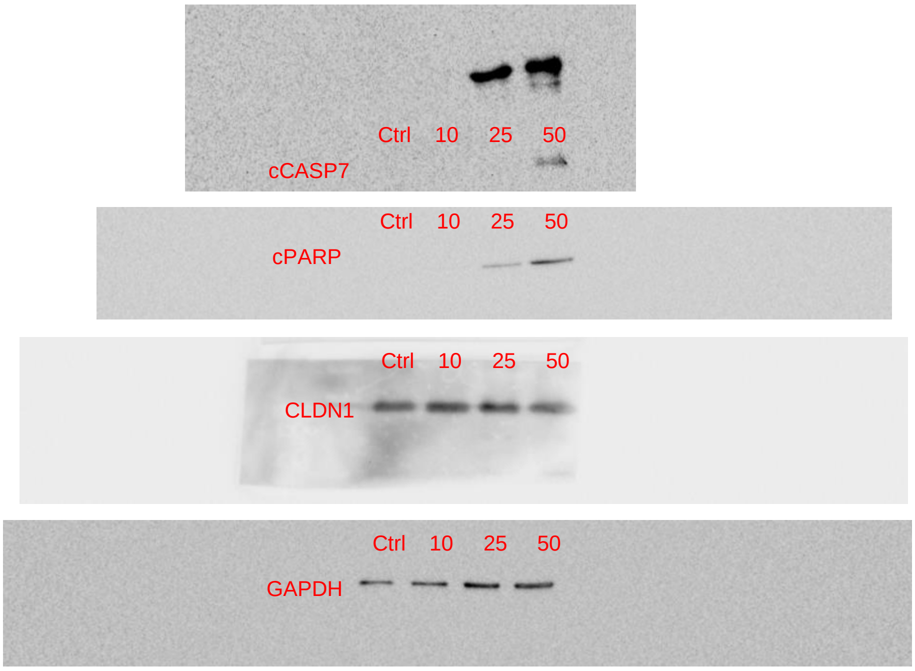

FIGURE 2F HCC1806 DOX n°4

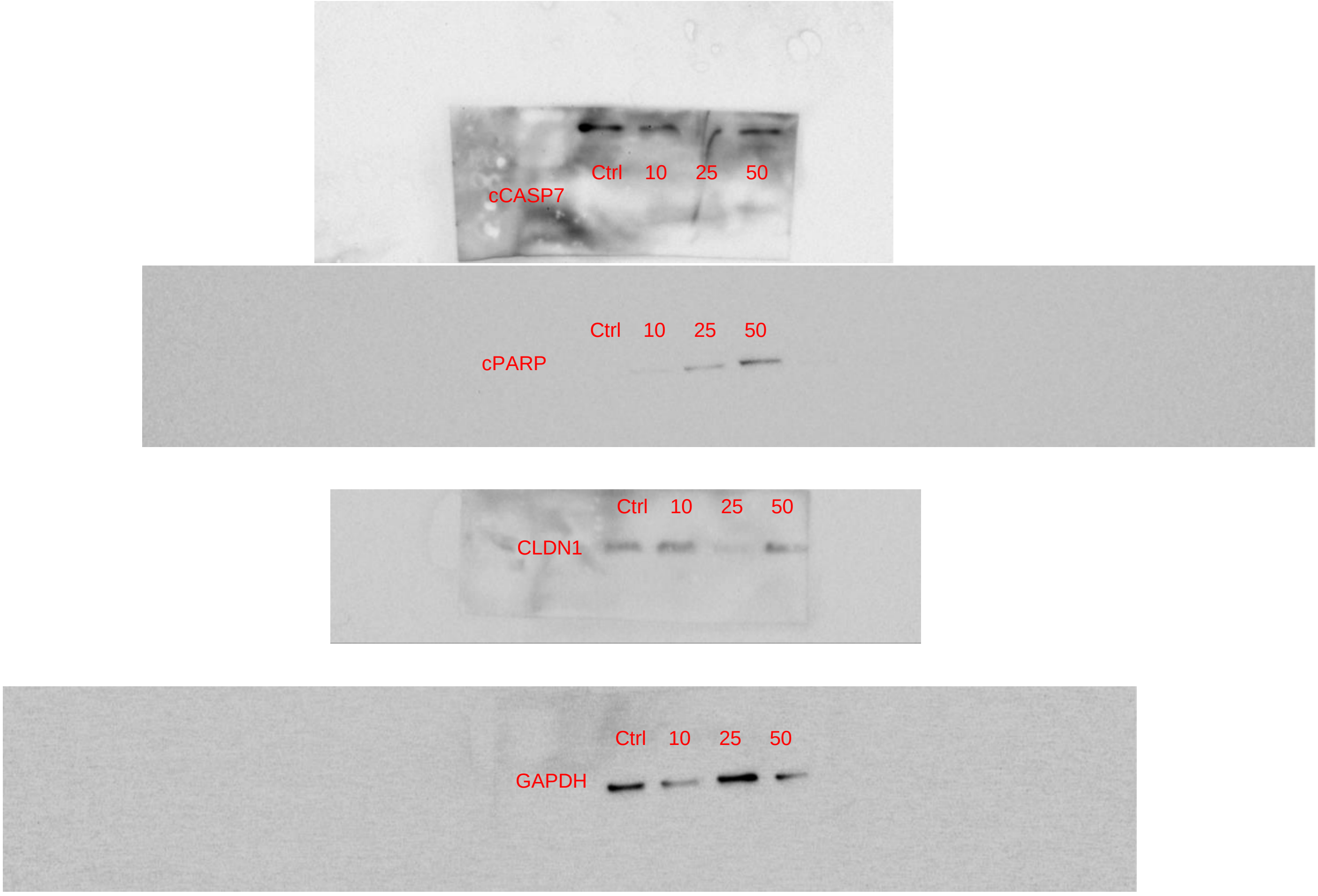

FIGURE 2F MDA-MB-231 DOX n°1

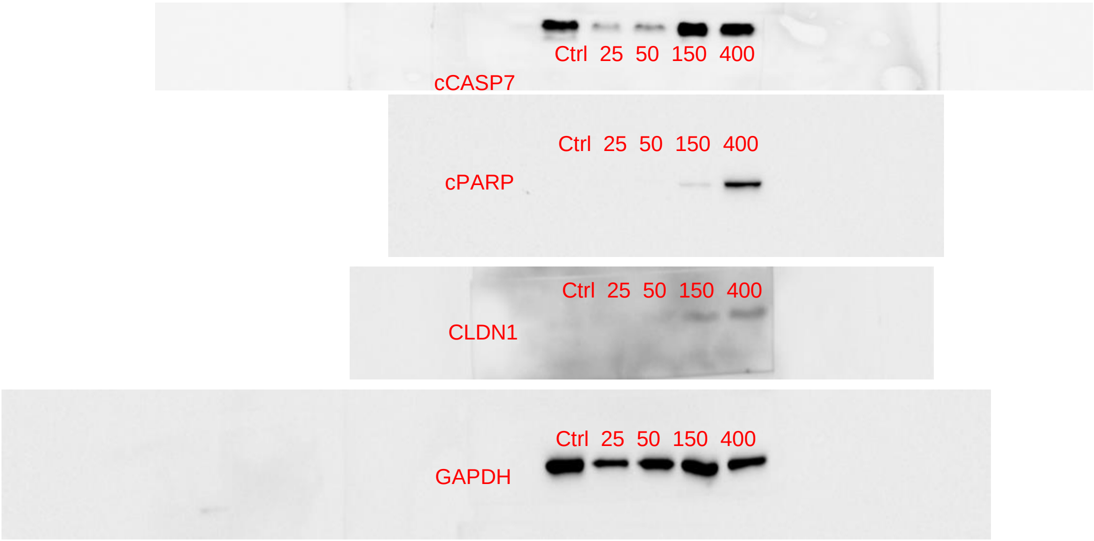

FIGURE 2F MDA-MB-231 DOX n°2

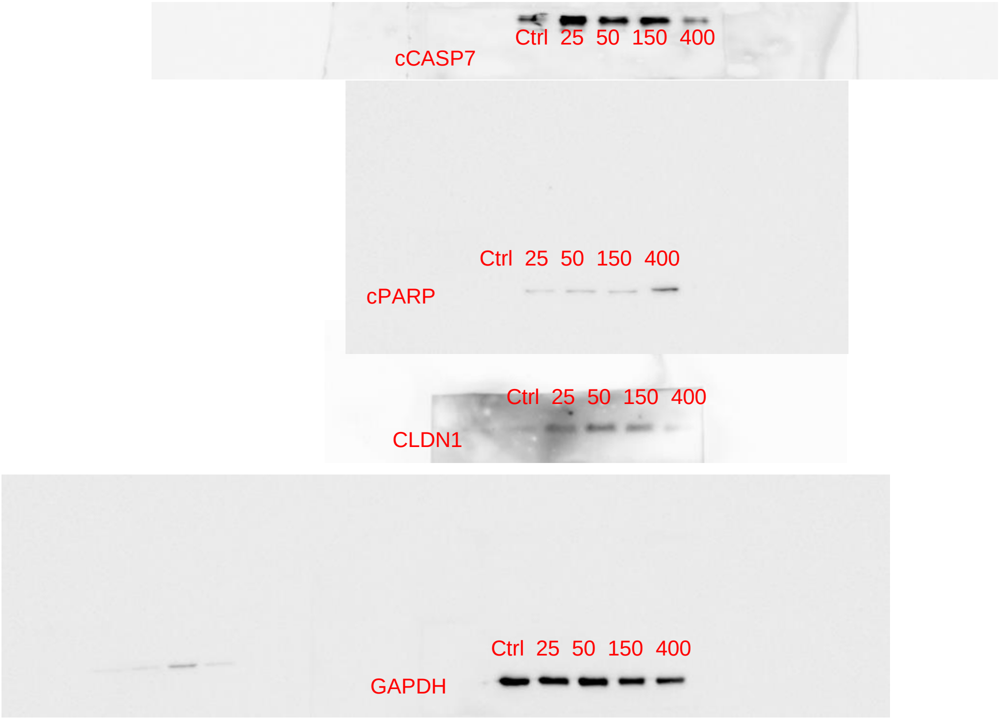

FIGURE 2F MDA-MB-231 DOX n°3

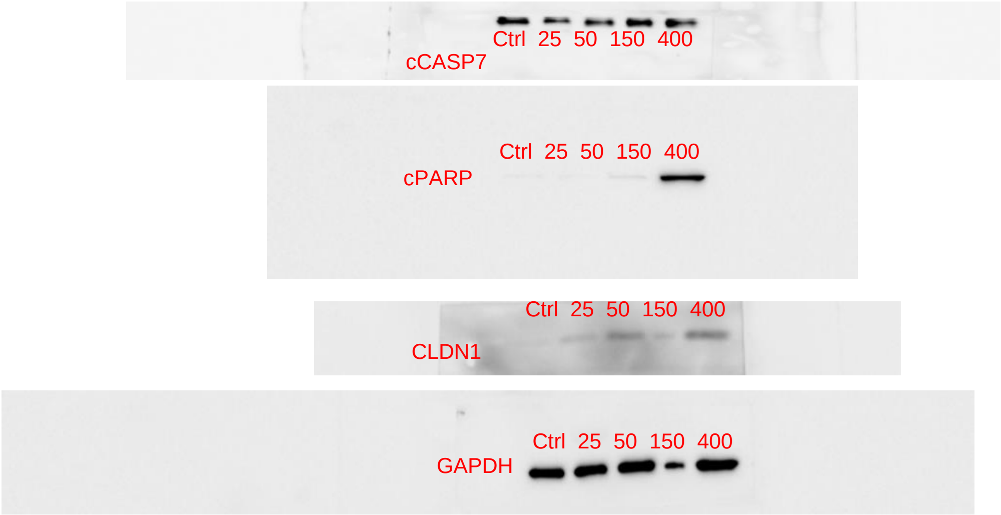

FIGURE 2F MDA-MB-231 DOX n°4

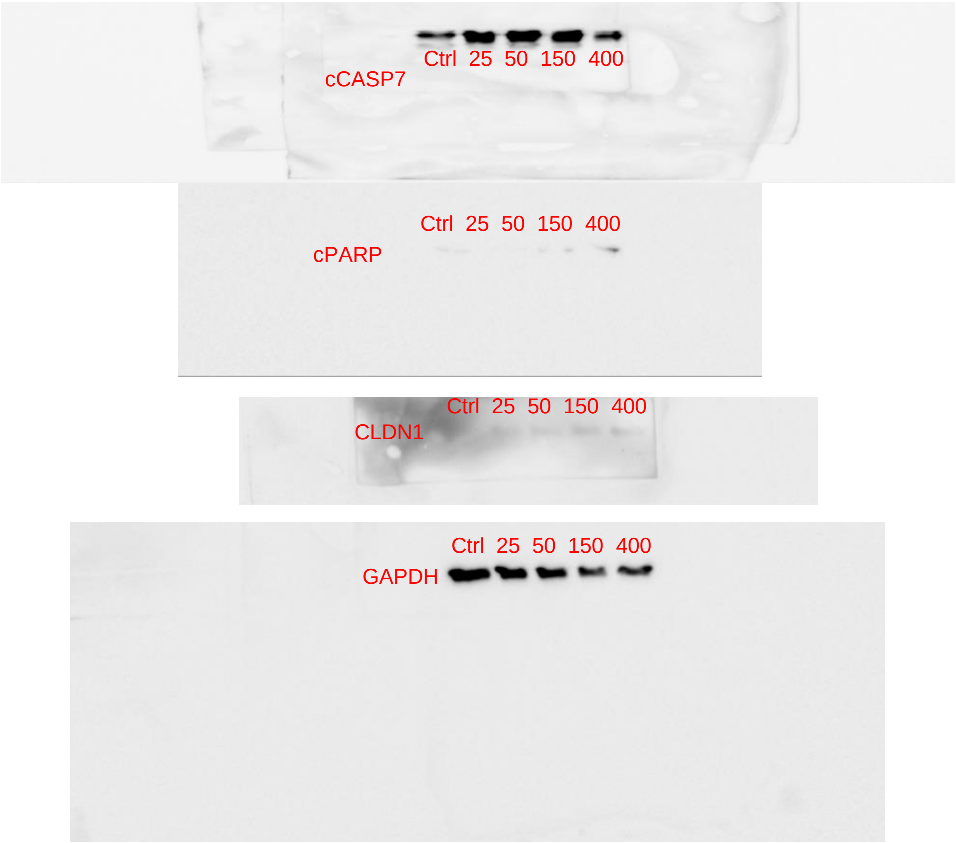

FIGURE 2F Hs578T DOX n°1

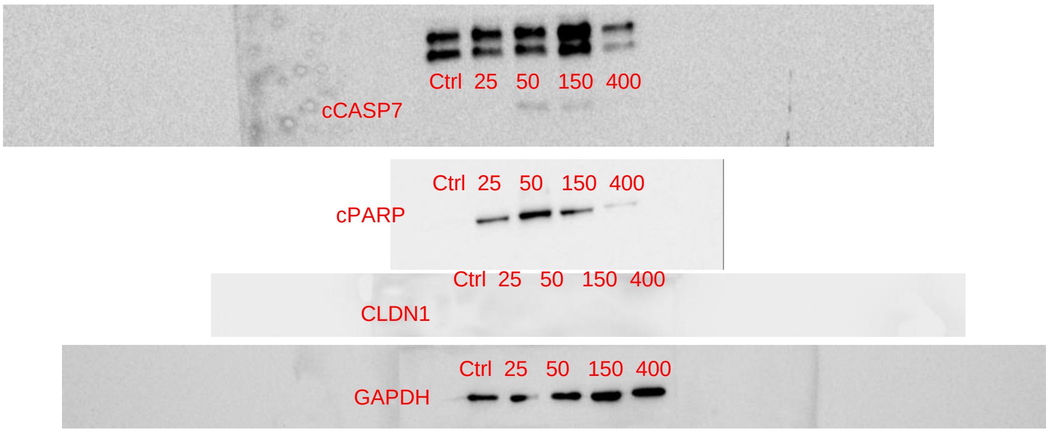

FIGURE 2F Hs578T DOX n°2

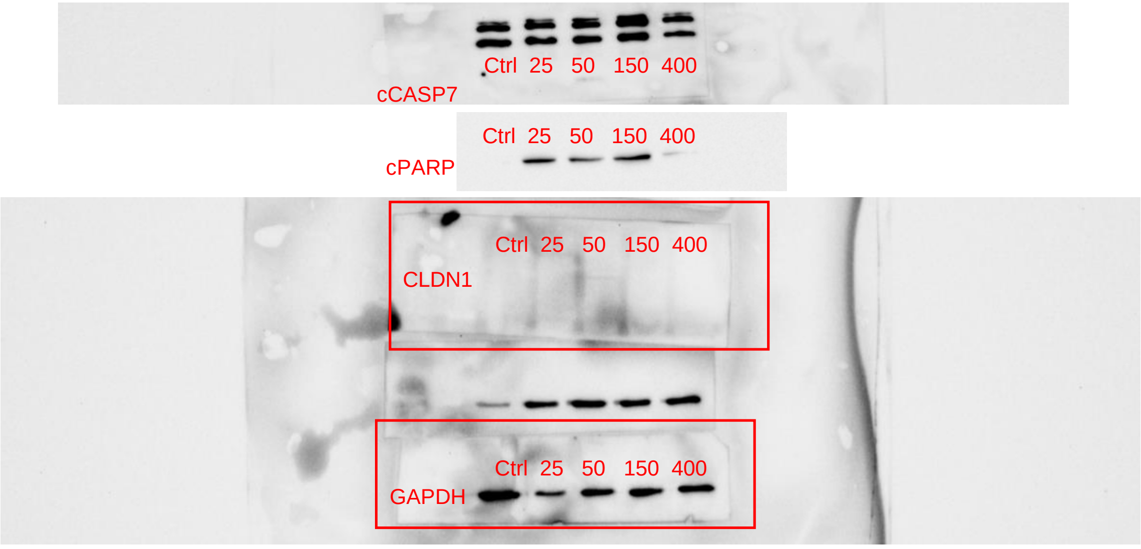

FIGURE 2F Hs578T DOX n°3

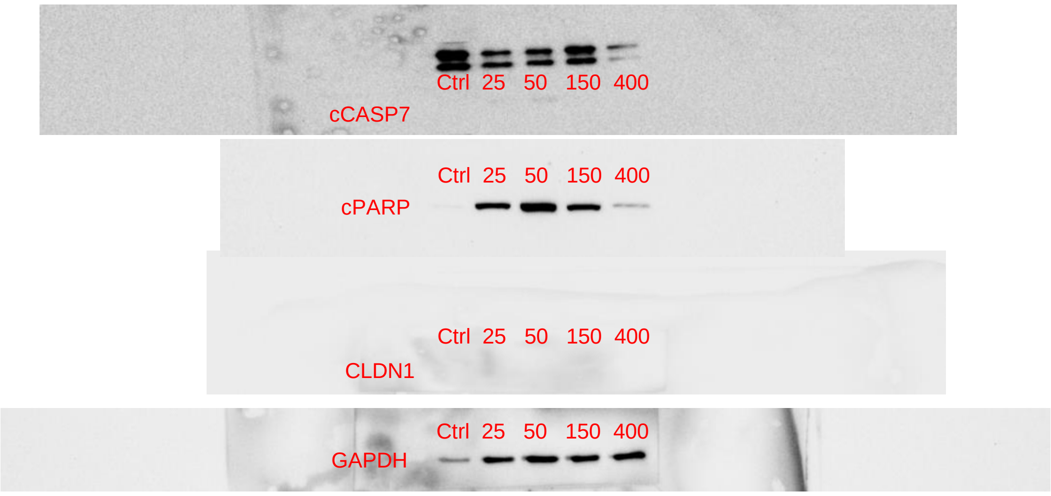

FIGURE 2F Hs578T DOX n°4

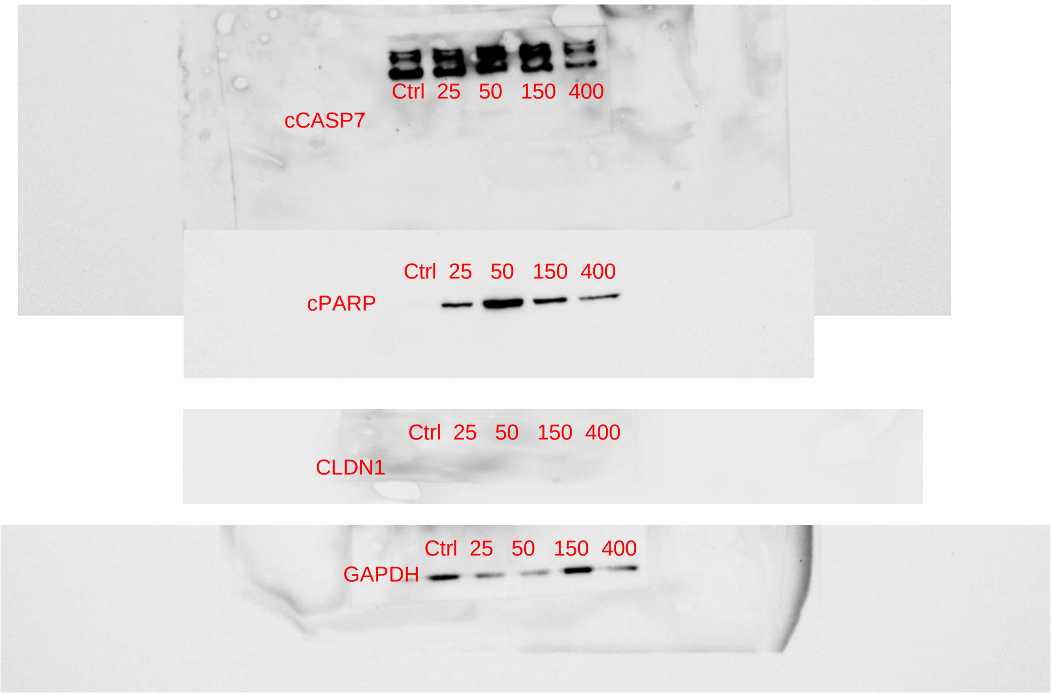

FIGURE 4D, G & J n°1

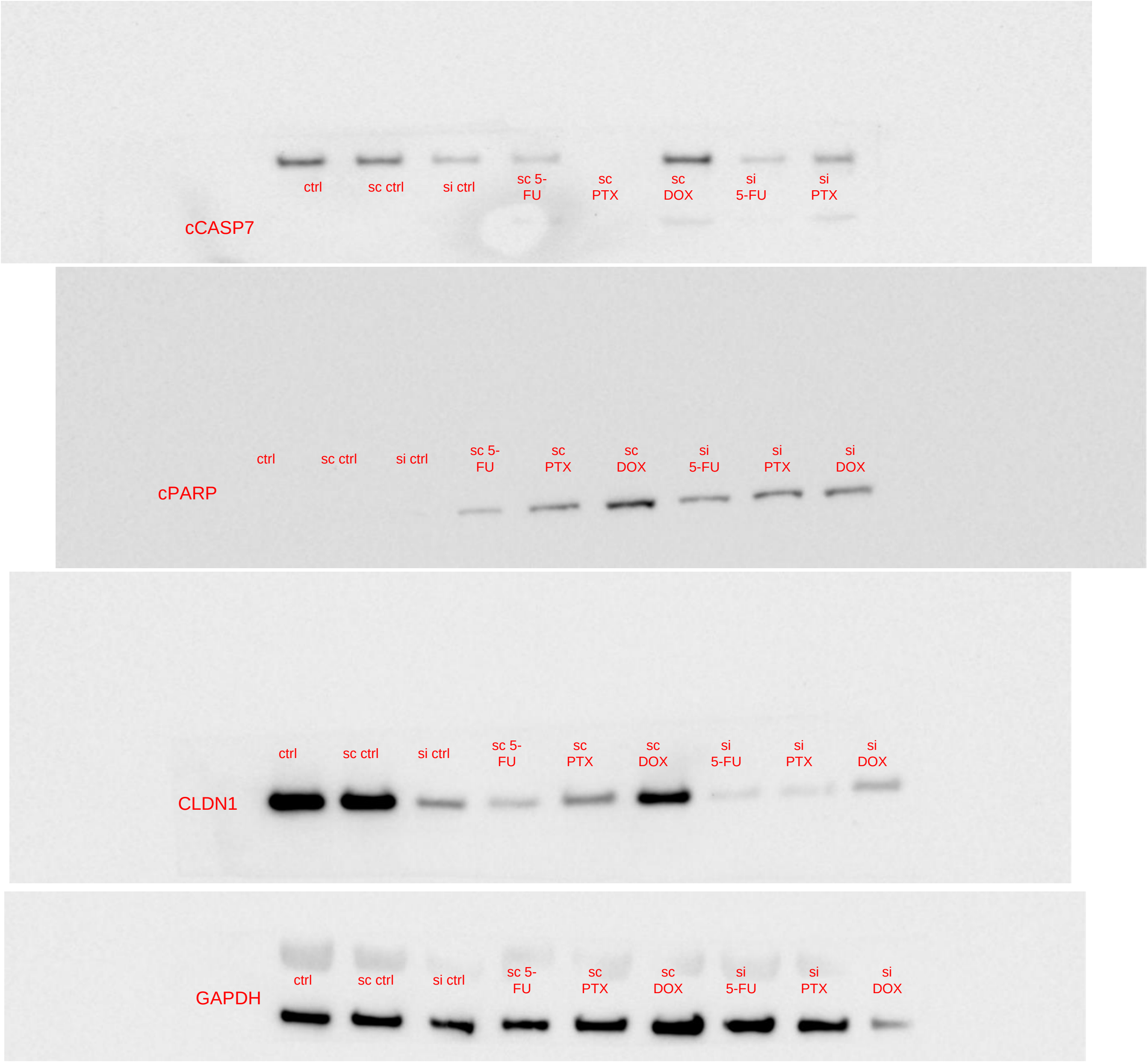

FIGURE 4D, G & J n°2

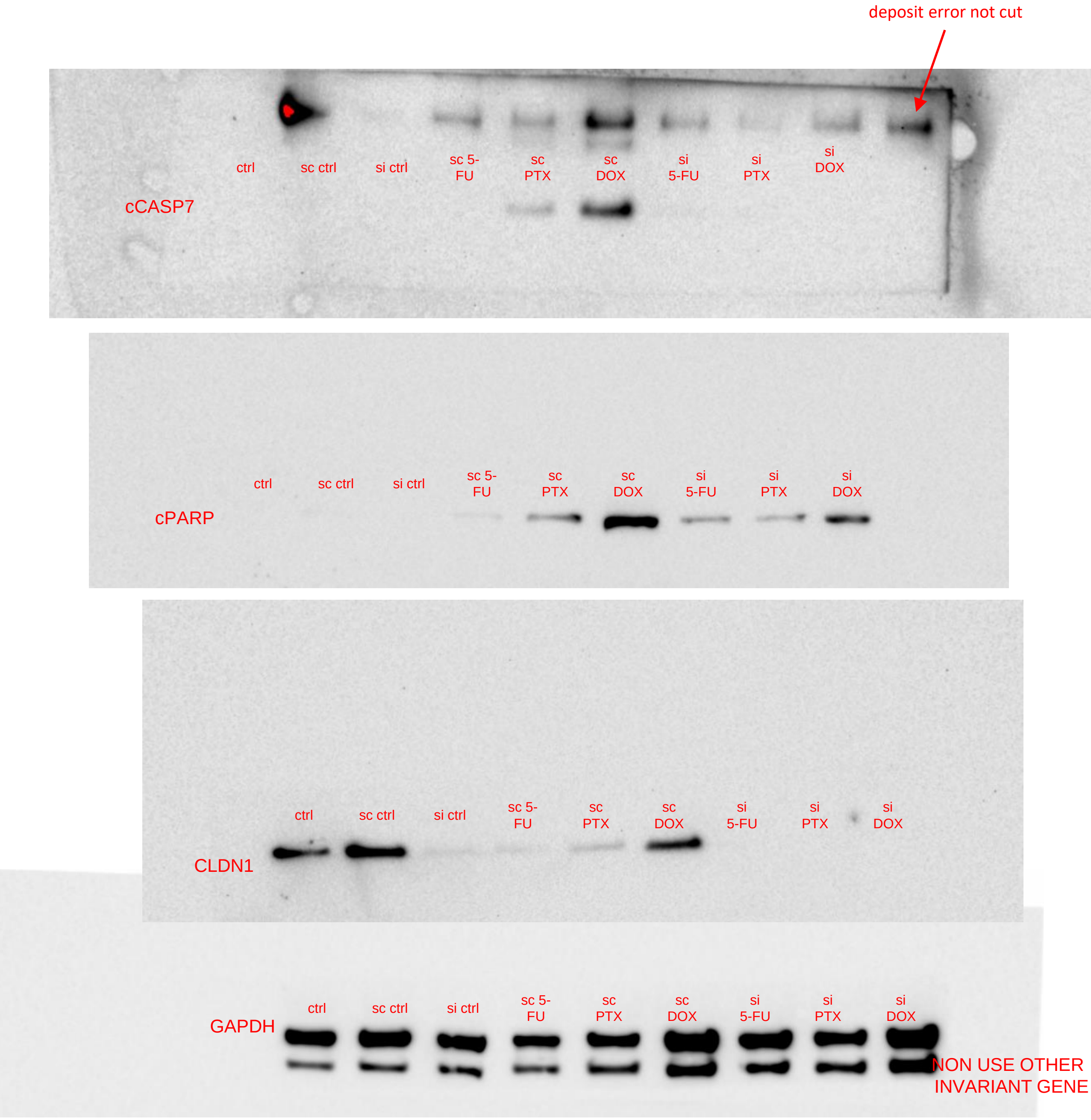

FIGURE 4D, G & J n°3

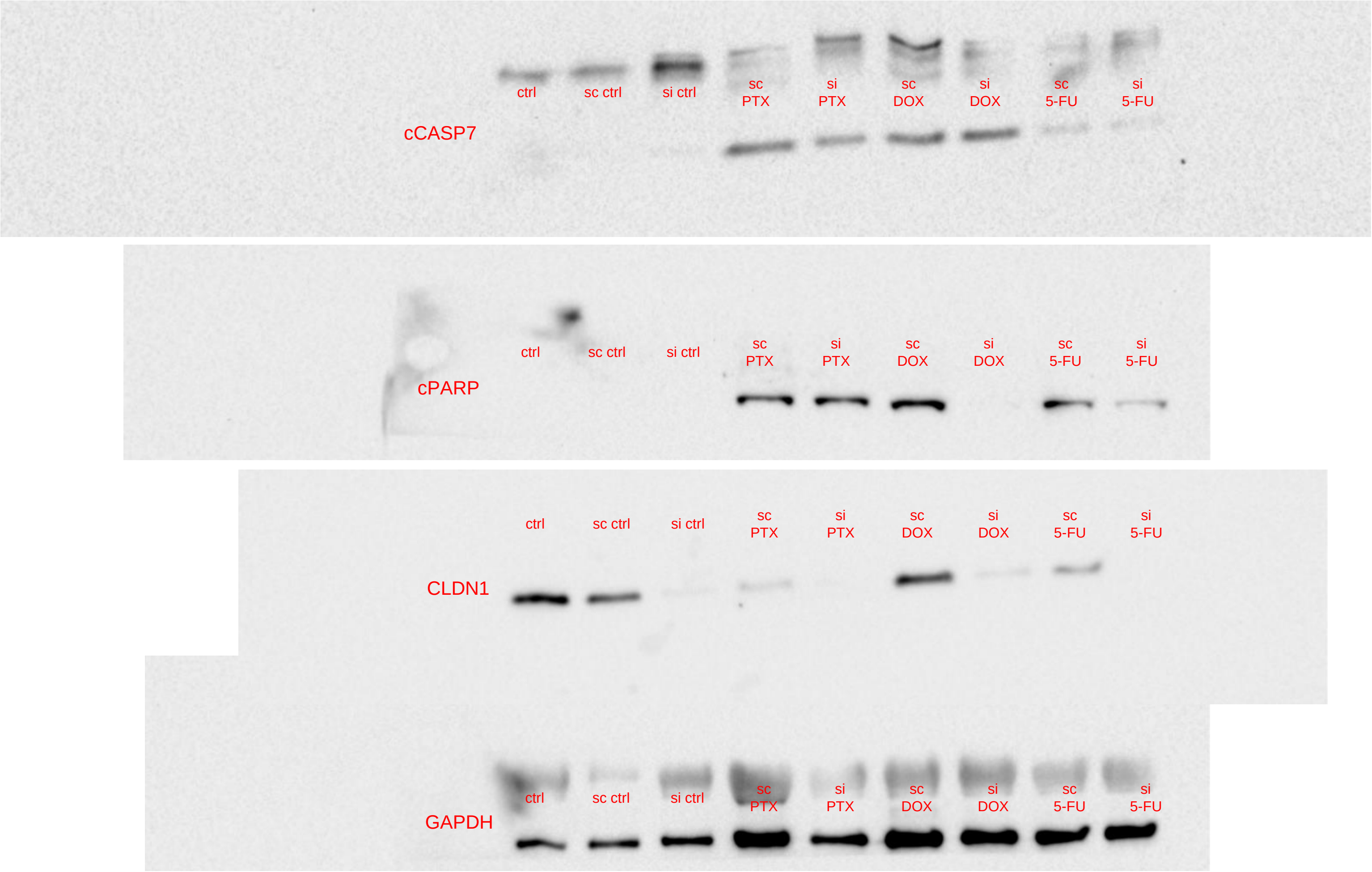

FIGURE 4D, G & J n°4

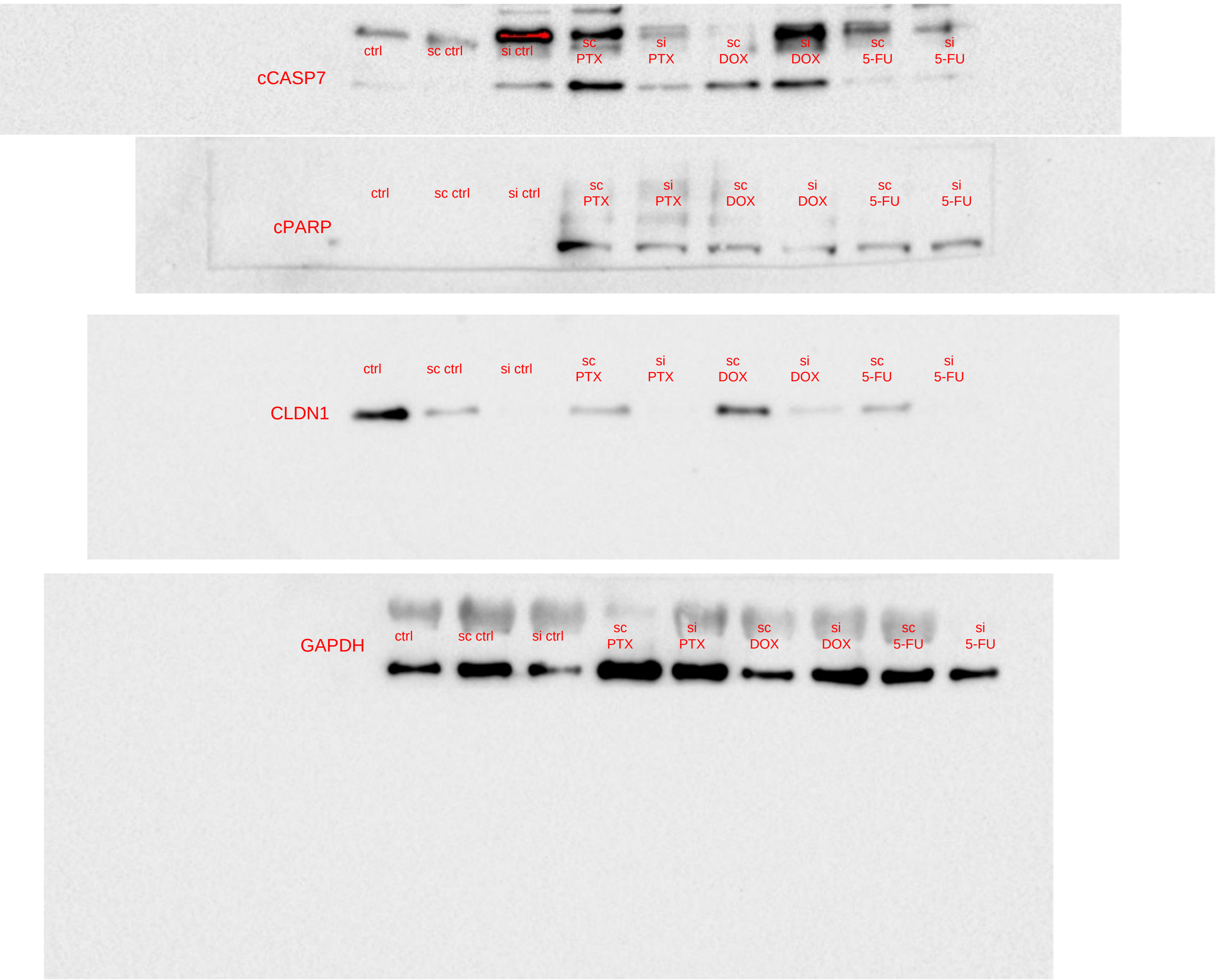

FIGURE 4D, G & J n°5

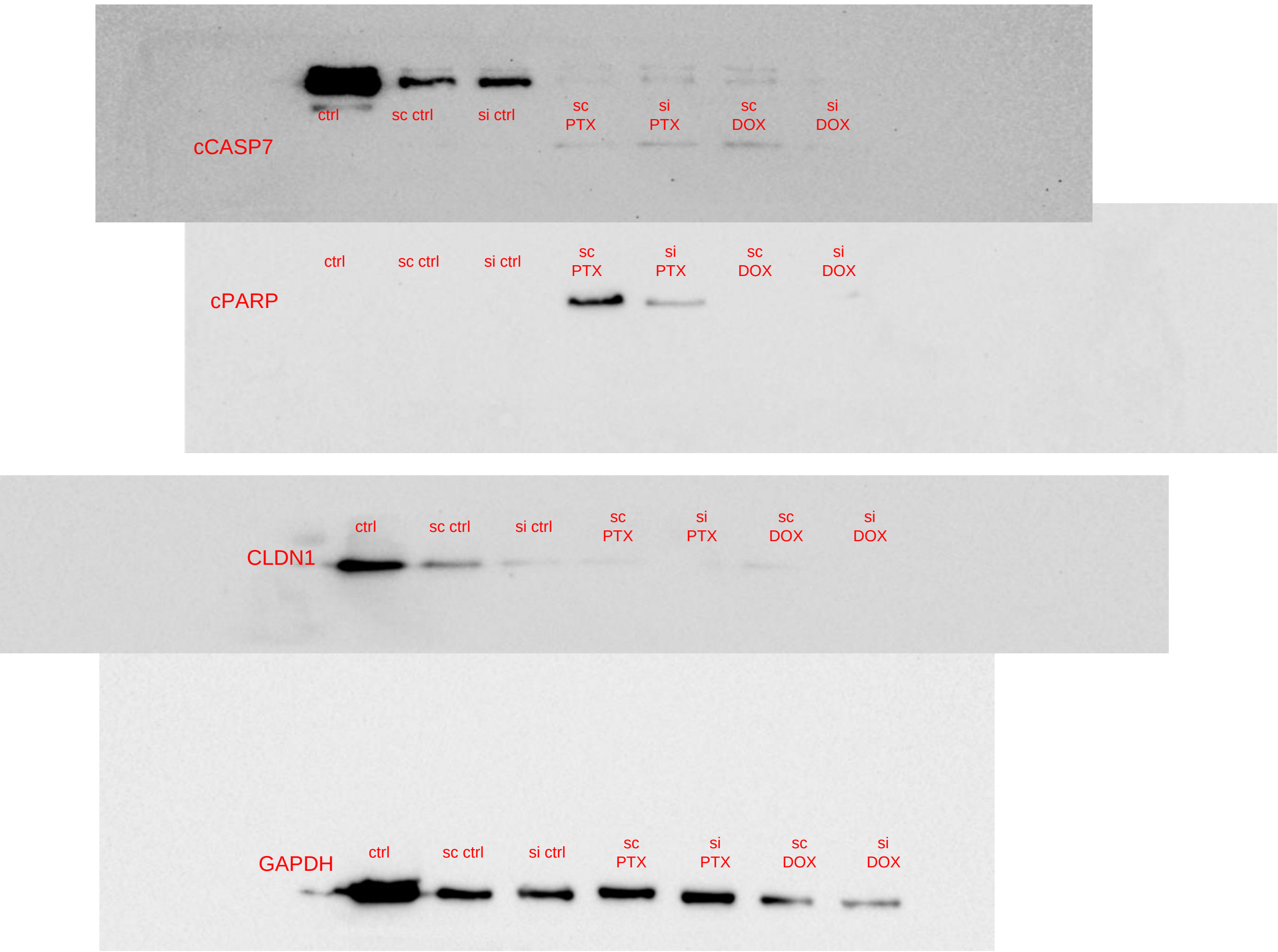

FIGURE 4D, G & J n°6

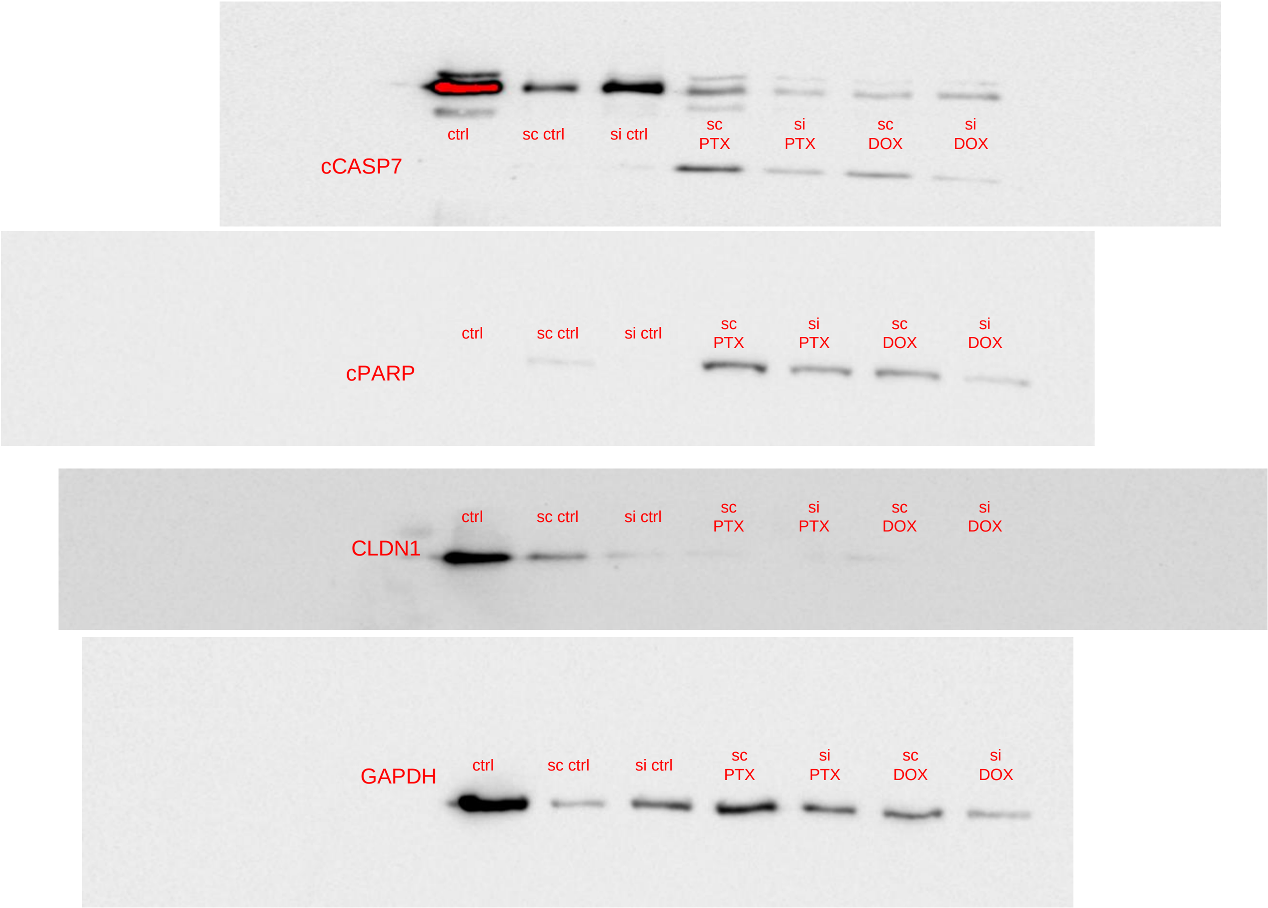

FIGURE 4D, G & J n°6

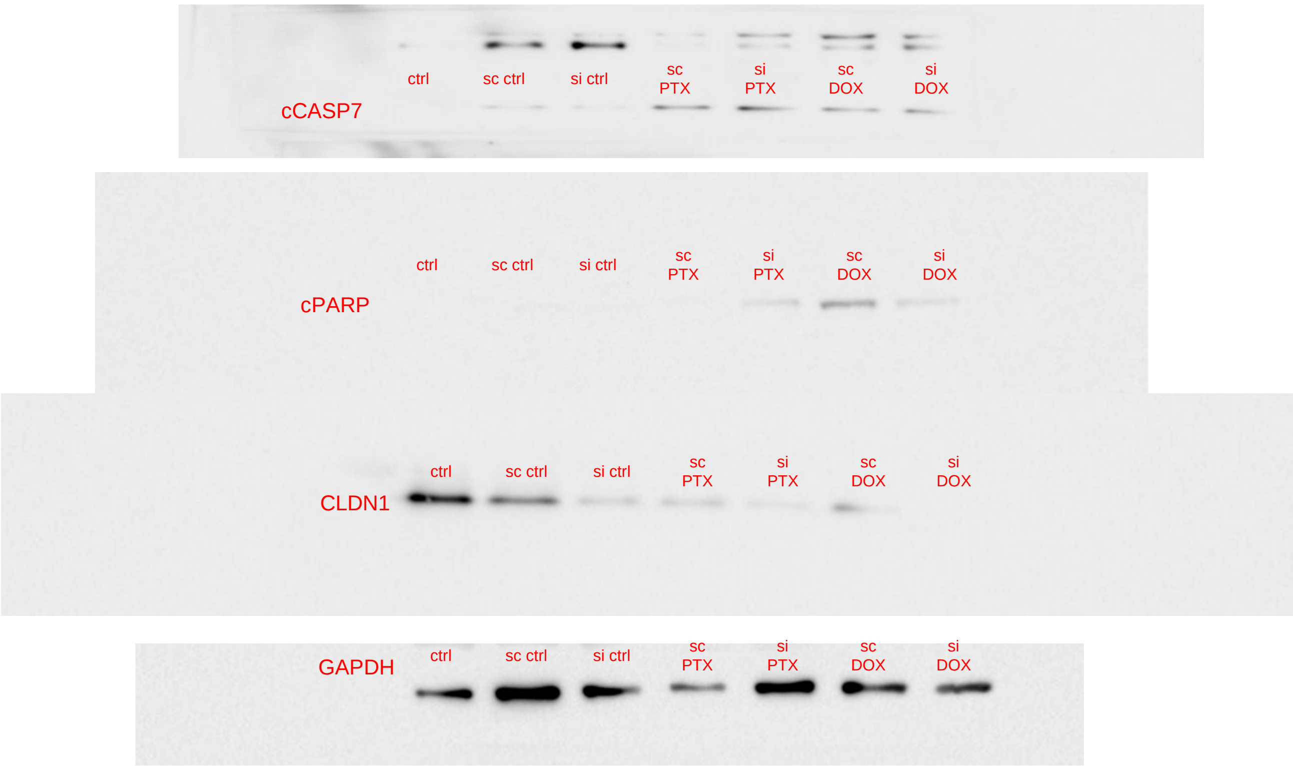

FIGURE 4D, G & J n°7

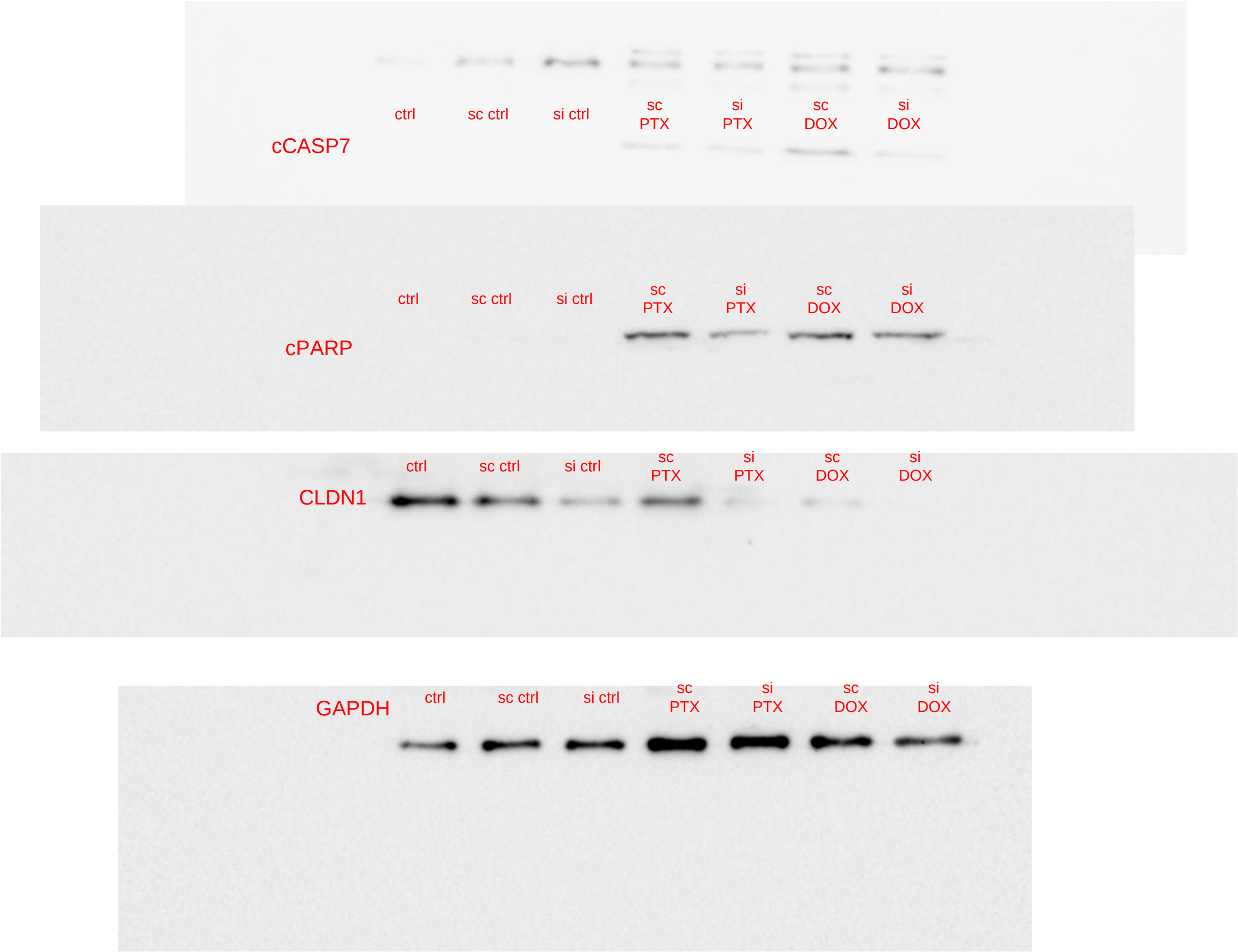

FIGURE 4D, G & J n°8

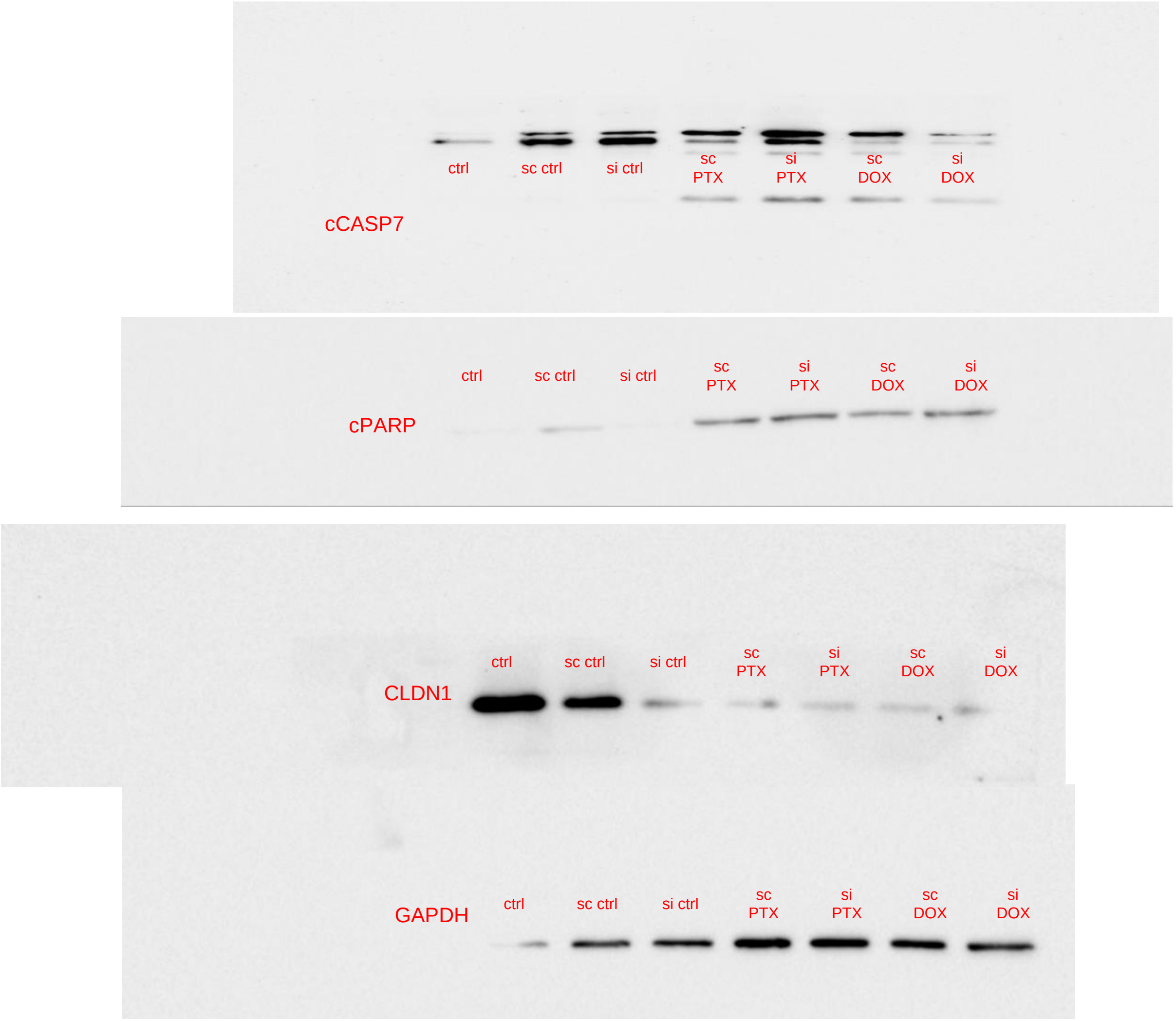

FIGURE 5C n°1 & 2

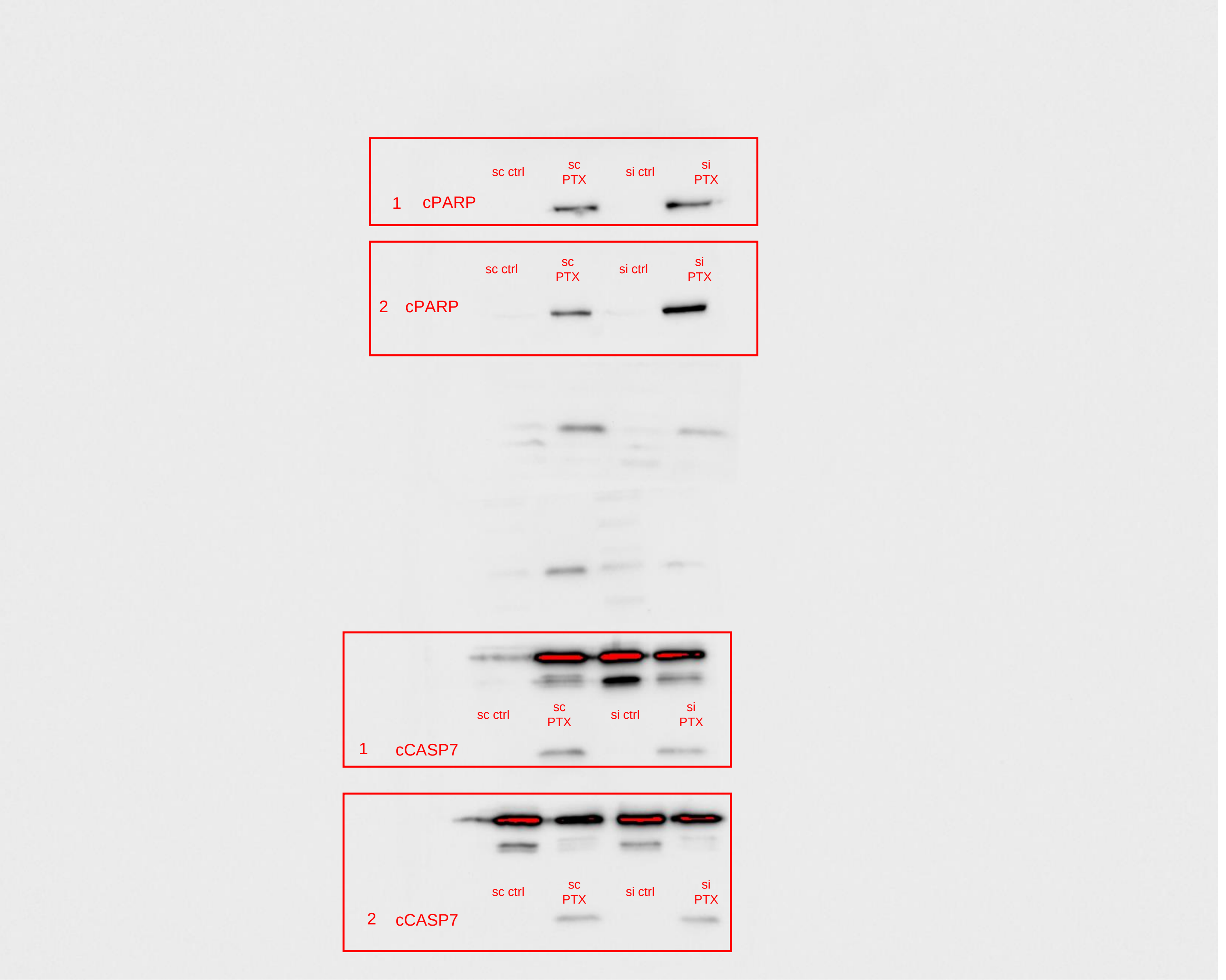

FIGURE 5C n°1 & 2

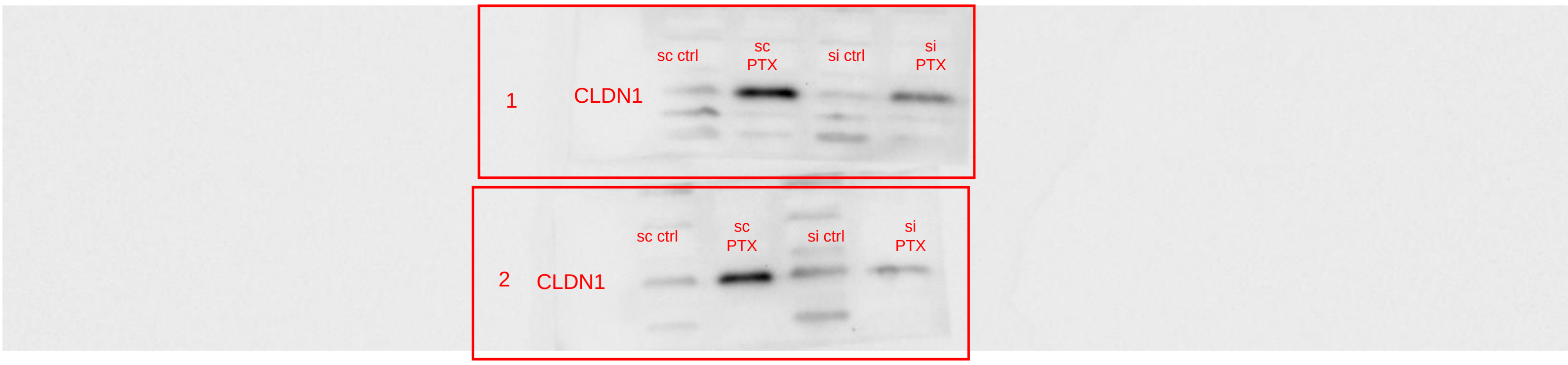

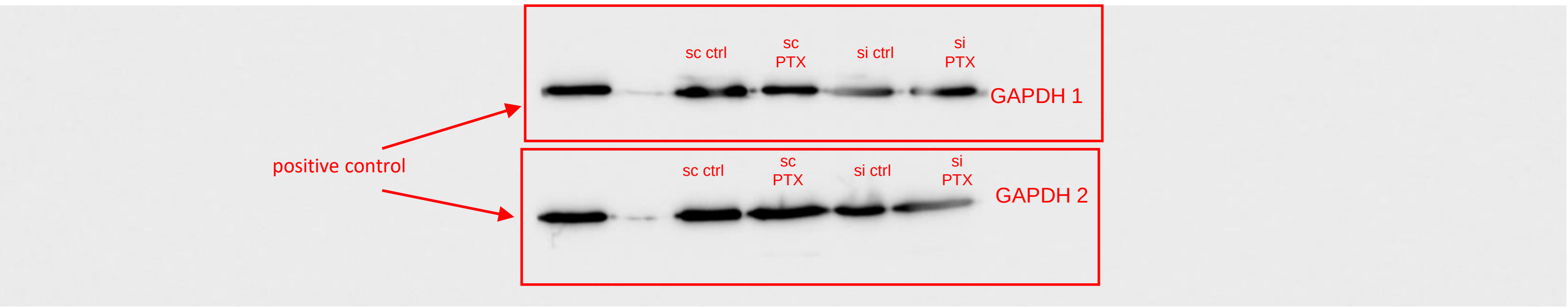

FIGURE 5C n°3 & 4

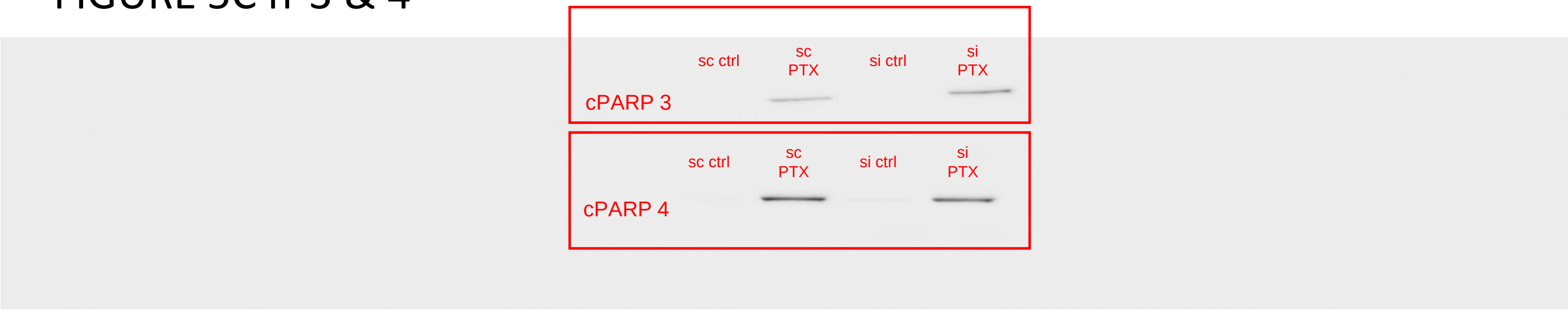

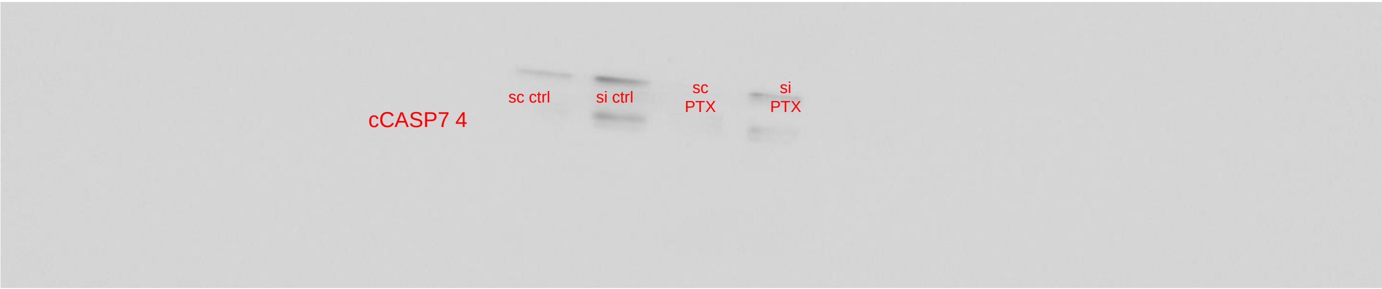

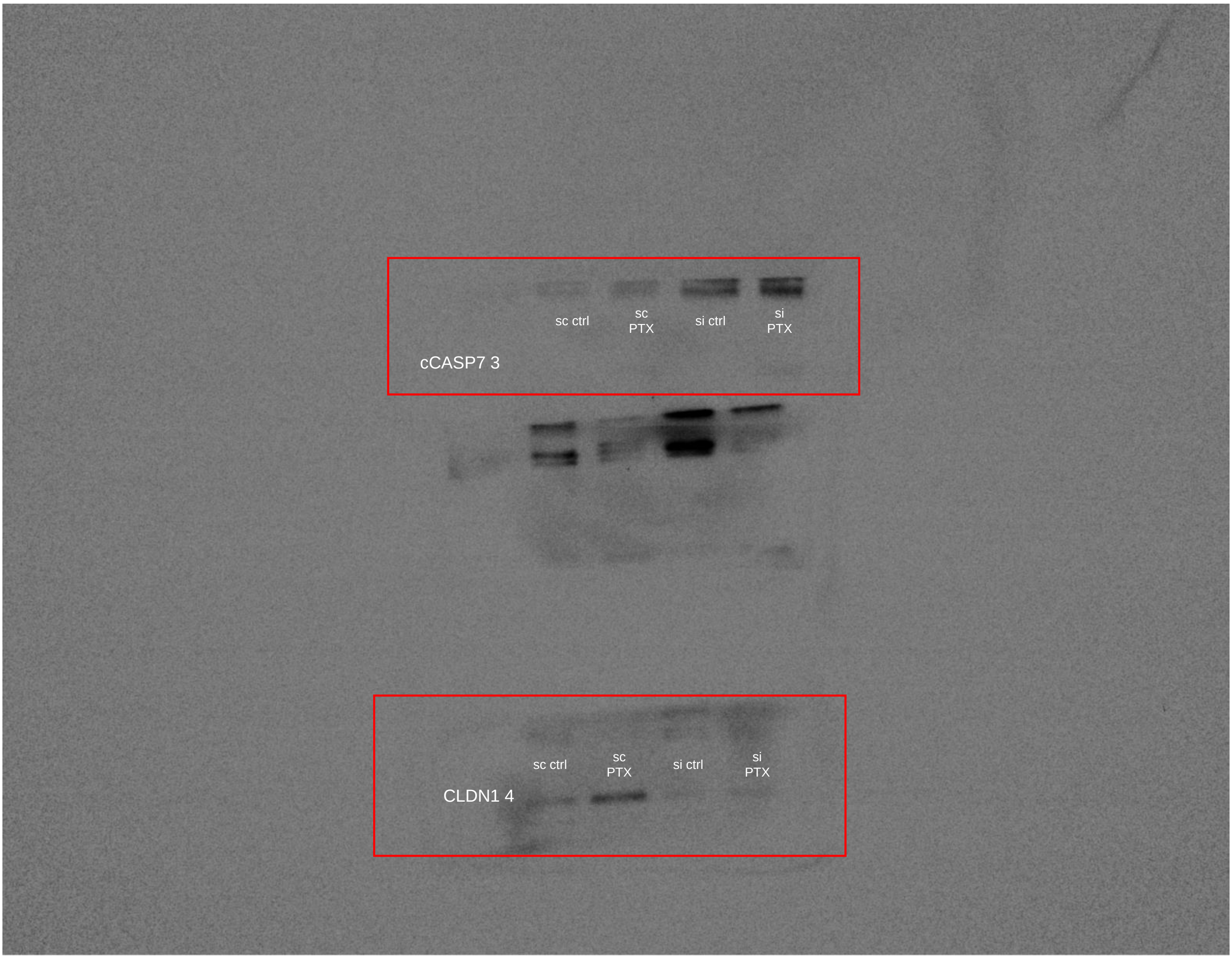

FIGURE 5C n°3 & 4

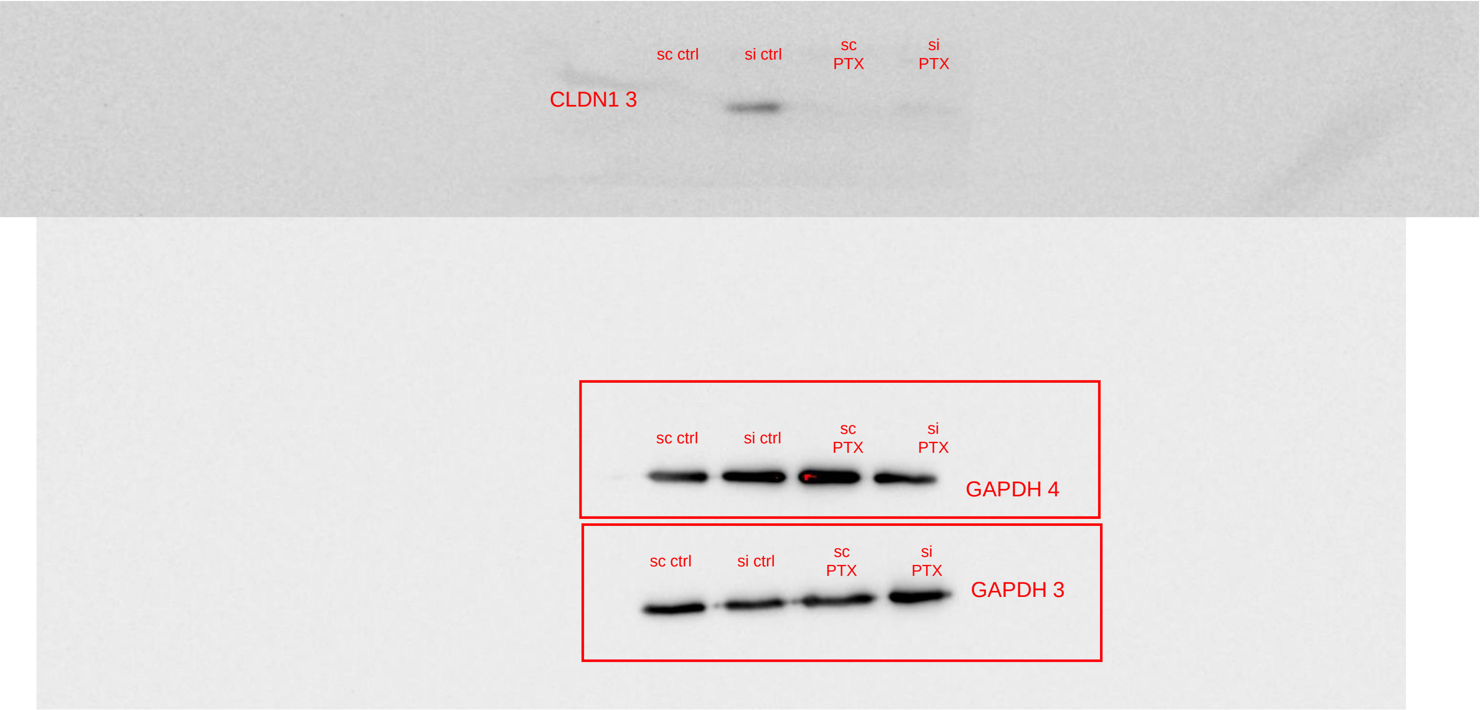

FIGURE 6F n°1, 2 & 3

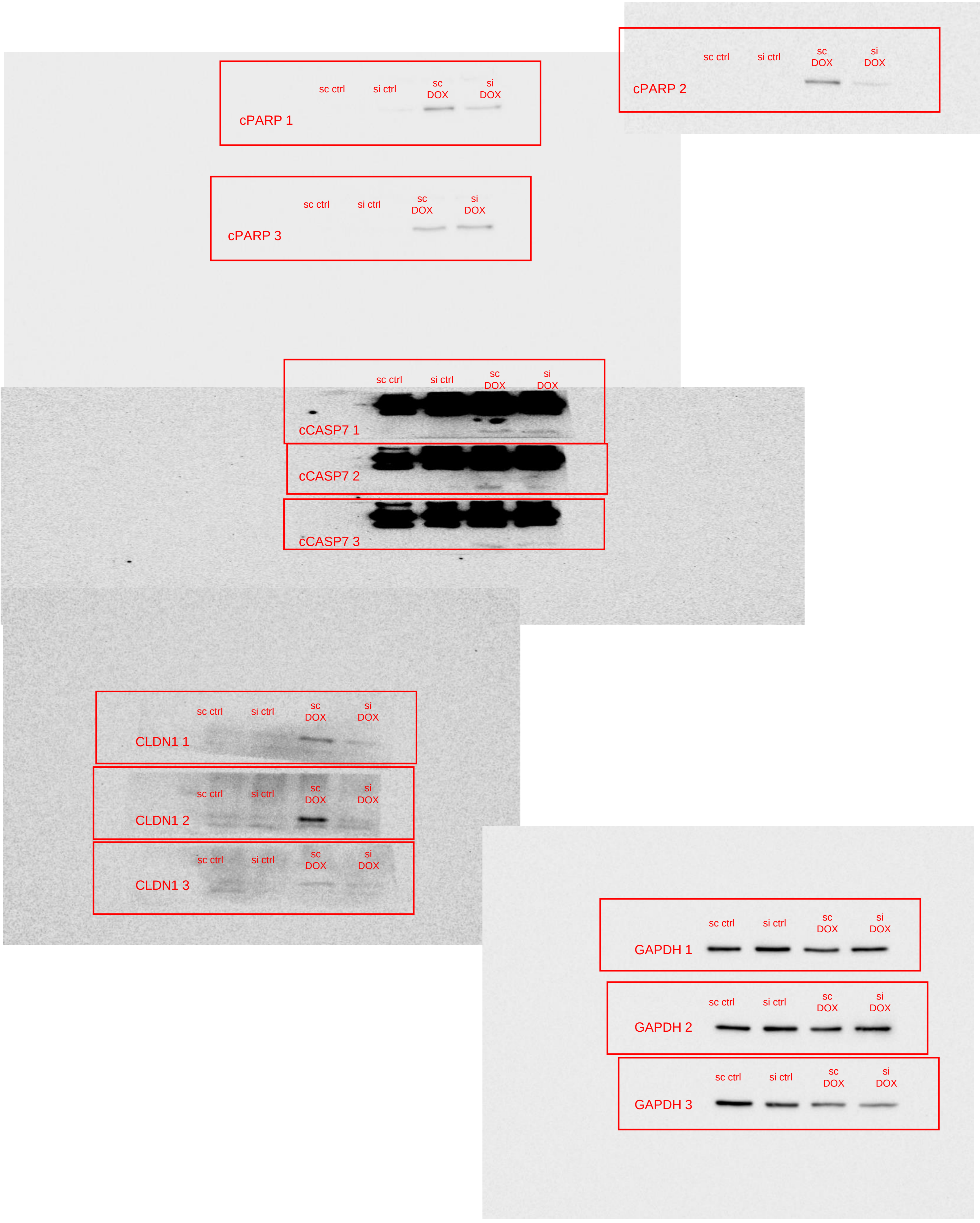

FIGURE 6F n° 4 & 5

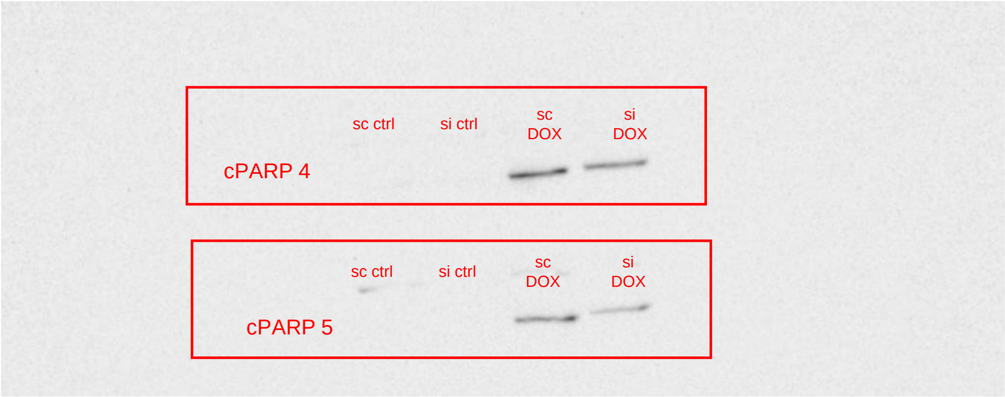

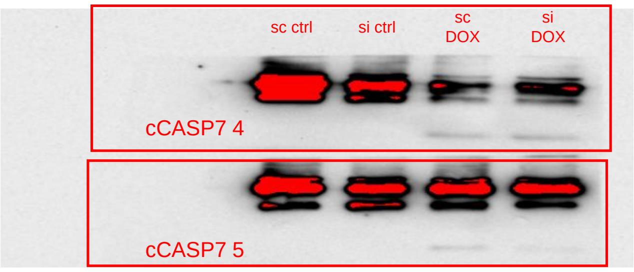

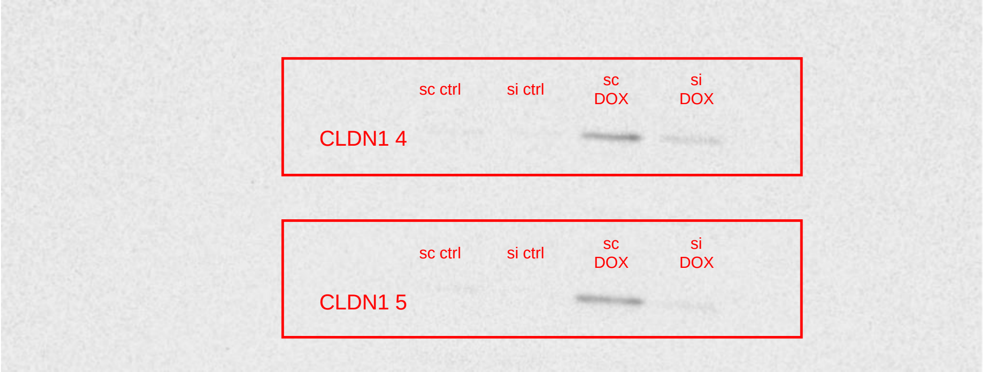

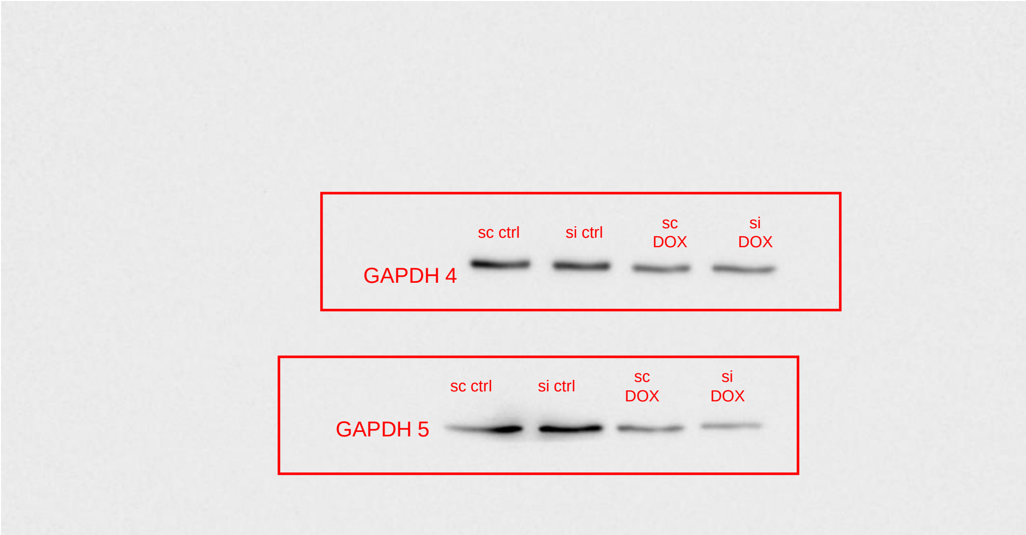

FIGURE 6C n° 1 & 2

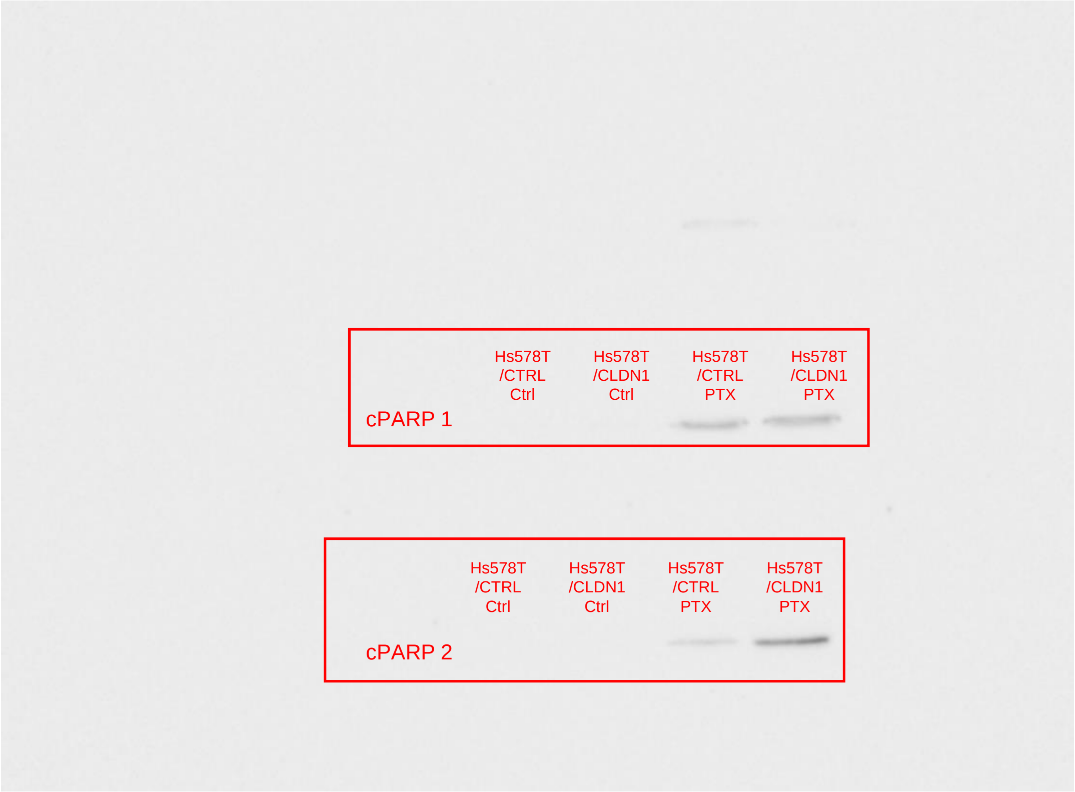

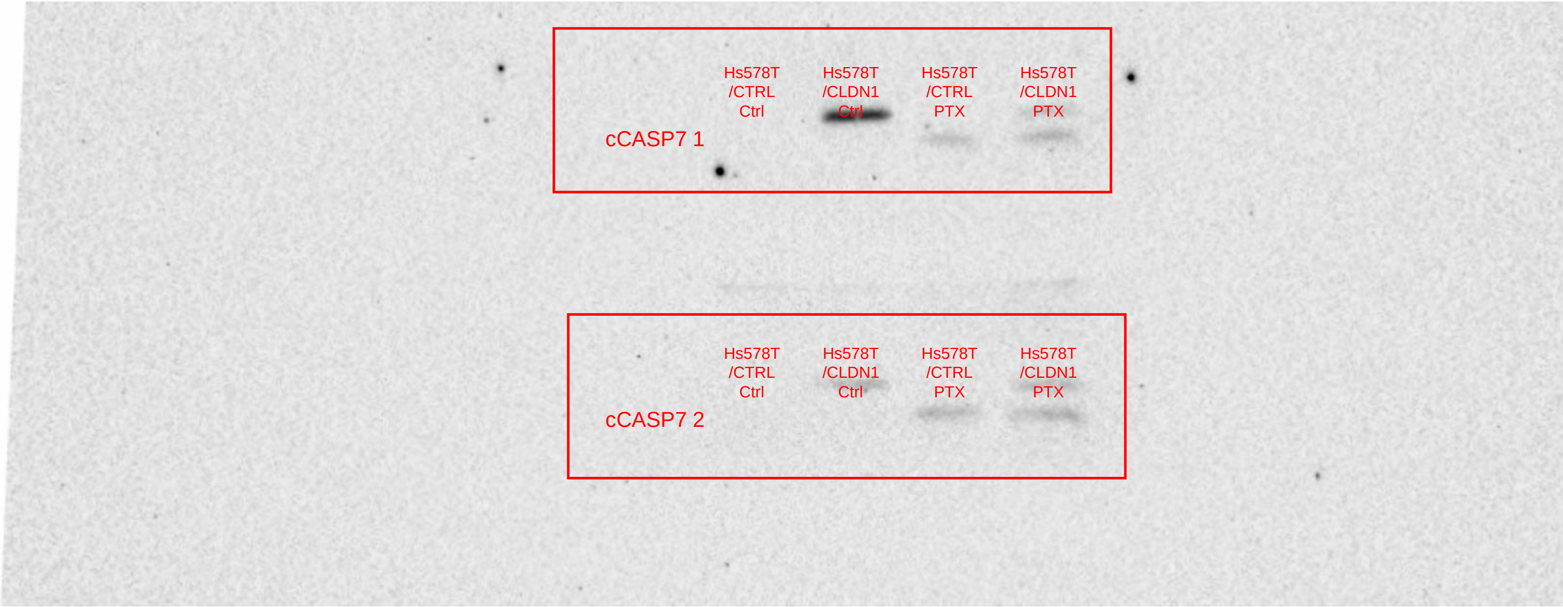

FIGURE 6C n° 1 & 2

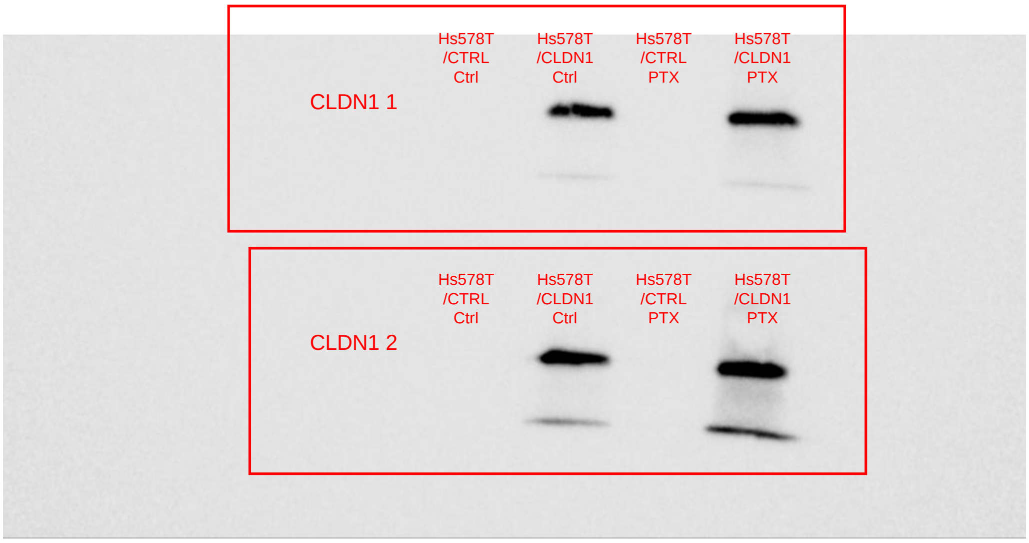

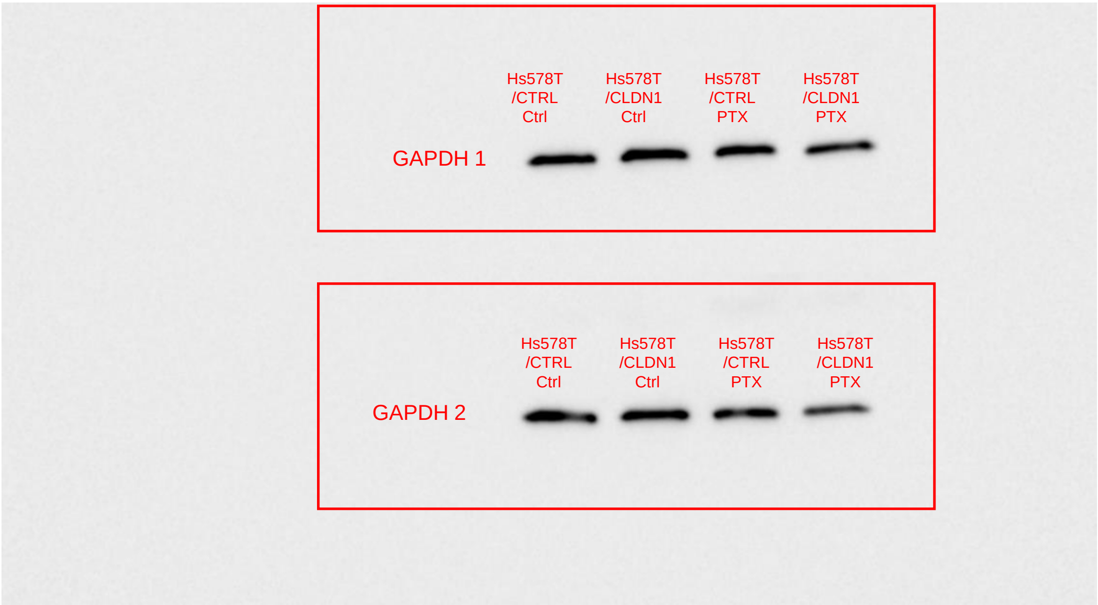

FIGURE 6C n° 3

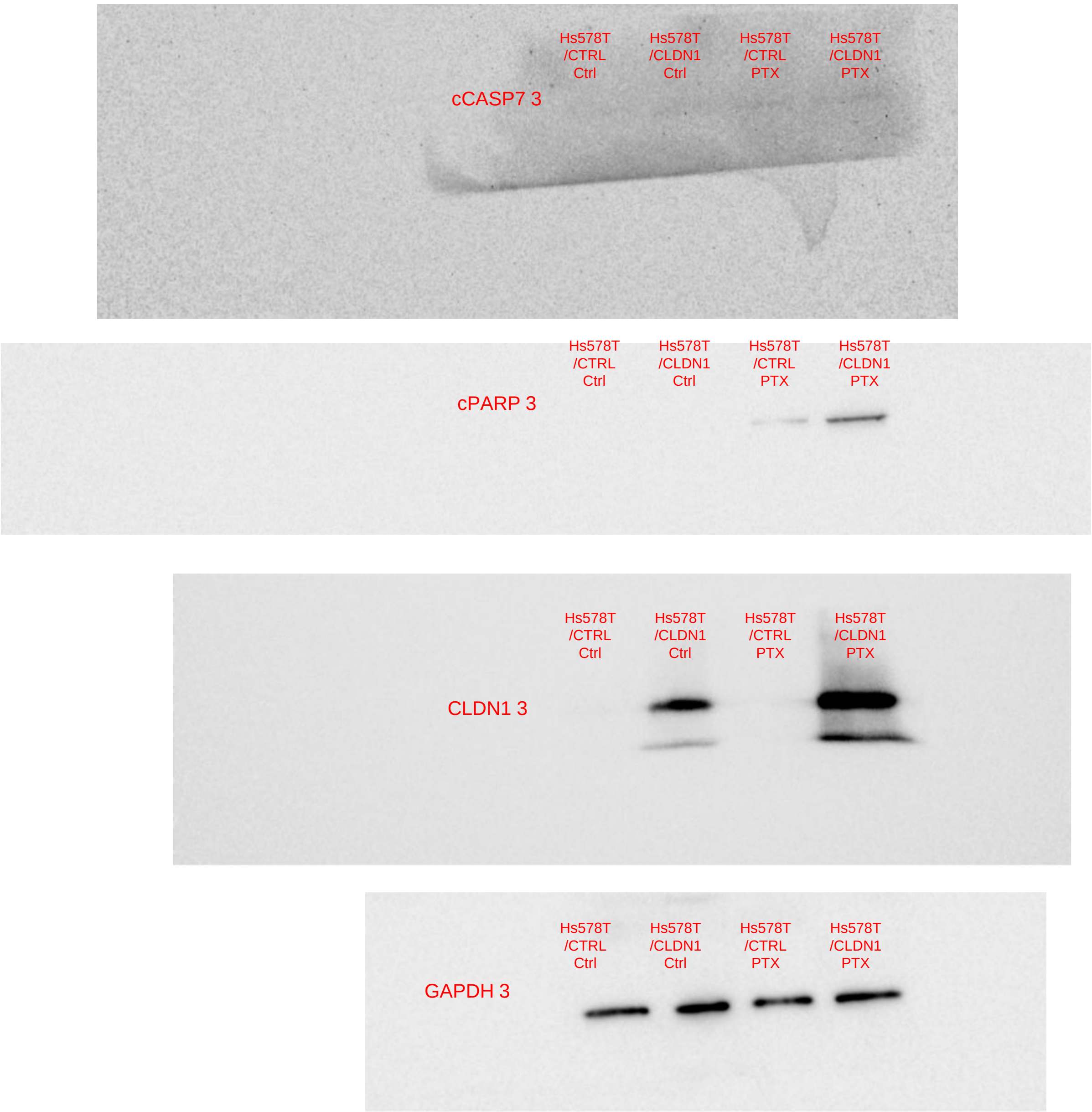

Hs578T /CTRL Ctrl
Hs578T /CLDN1 Ctrl
Hs578T /CTRL PTX
Hs578T /CLDN1 PTX
cCASP7 3
Hs578T /CTRL Ctrl
Hs578T /CLDN1 Ctrl
Hs578T /CTRL PTX
Hs578T /CLDN1 PTX
cPARP 3
Hs578T /CTRL Ctrl
Hs578T /CLDN1 Ctrl
Hs578T /CTRL PTX
Hs578T /CLDN1 PTX
CLDN1 3
Hs578T /CTRL Ctrl
Hs578T /CLDN1 Ctrl
Hs578T /CTRL PTX
Hs578T /CLDN1 PTX
GAPDH 3

FIGURE 6E n° 1

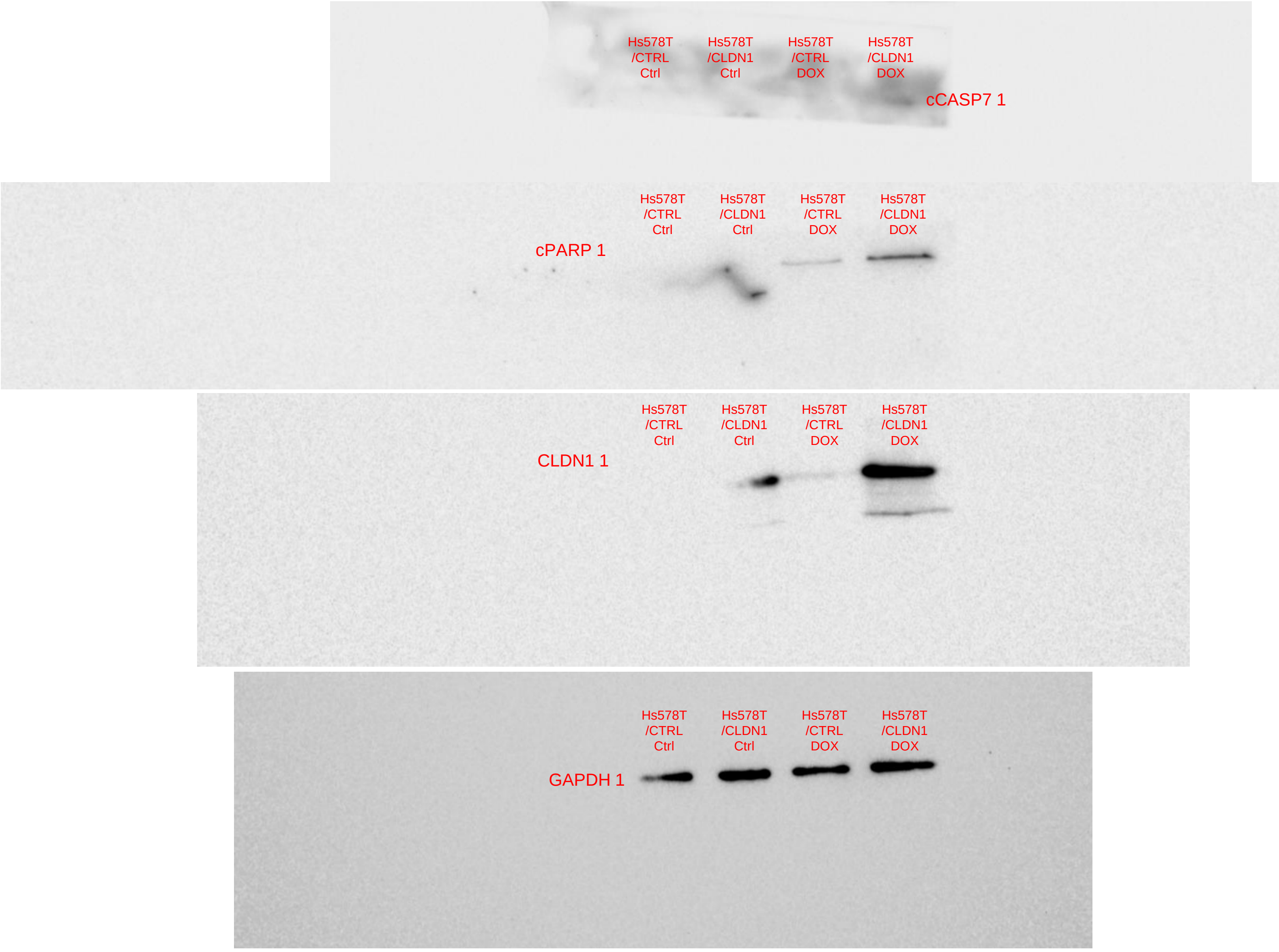
Hs578T /CTRL Ctrl
Hs578T /CLDN1 Ctrl
Hs578T /CTRL DOX
Hs578T /CLDN1 DOX
cCASP7 1
Hs578T /CTRL Ctrl
Hs578T /CLDN1 Ctrl
Hs578T /CTRL DOX
Hs578T /CLDN1 DOX
cPARP 1
Hs578T /CTRL Ctrl
Hs578T /CLDN1 Ctrl
Hs578T /CTRL DOX
Hs578T /CLDN1 DOX
CLDN1 1
Hs578T /CTRL Ctrl
Hs578T /CLDN1 Ctrl
Hs578T /CTRL DOX
Hs578T /CLDN1 DOX
GAPDH 1

FIGURE 6E n°2, 3 & 4

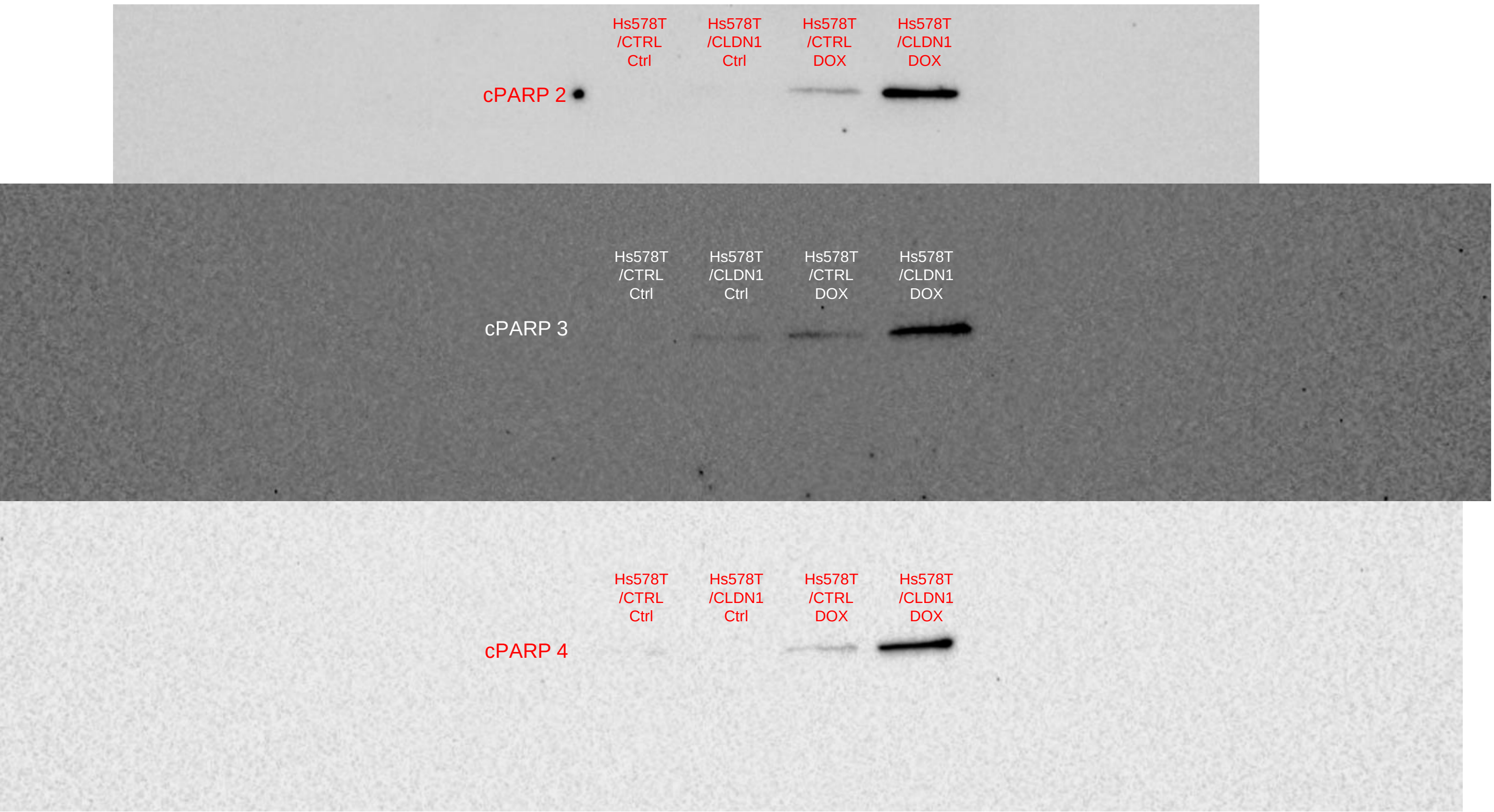

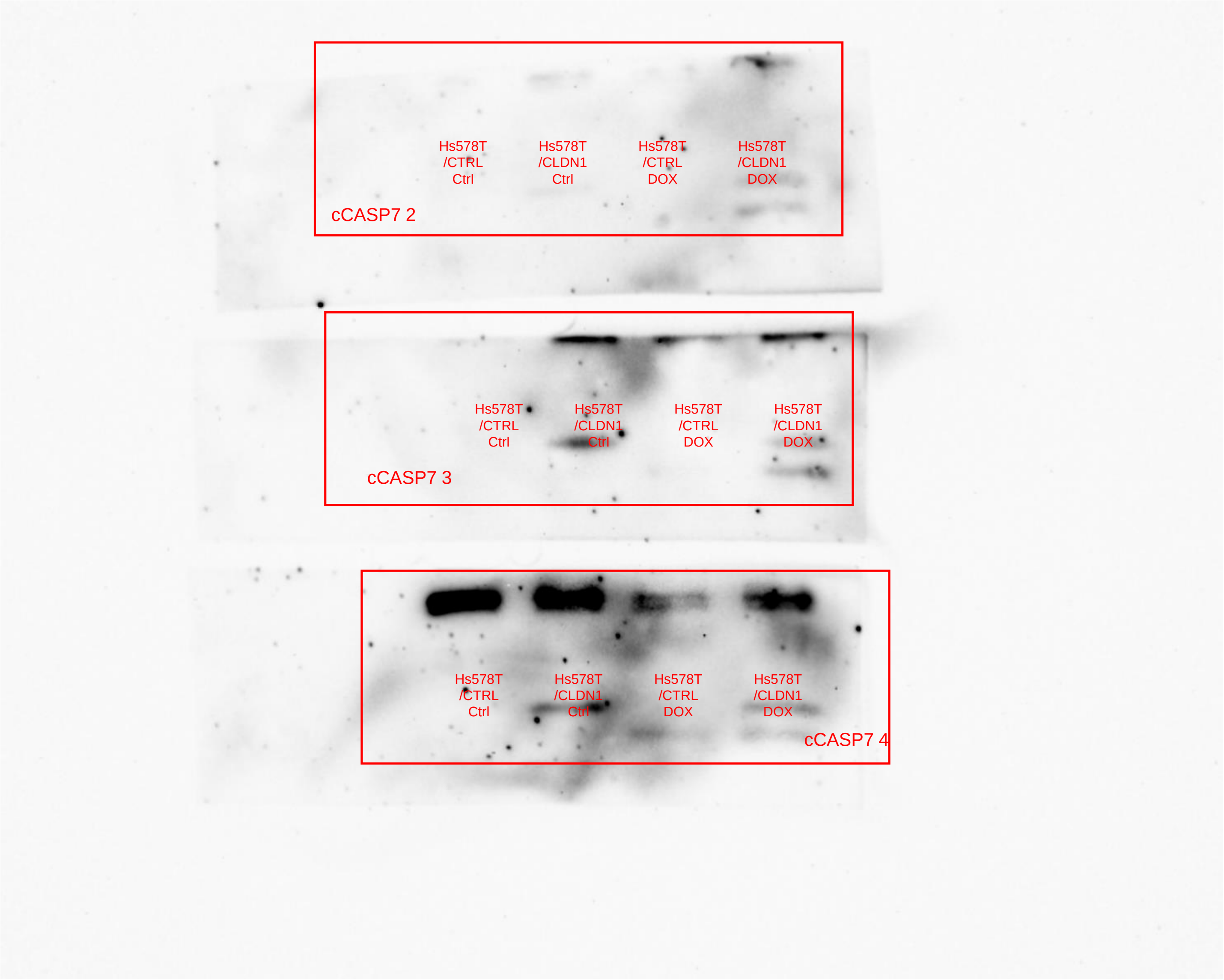

FIGURE 6D n° 2, 3 & 4

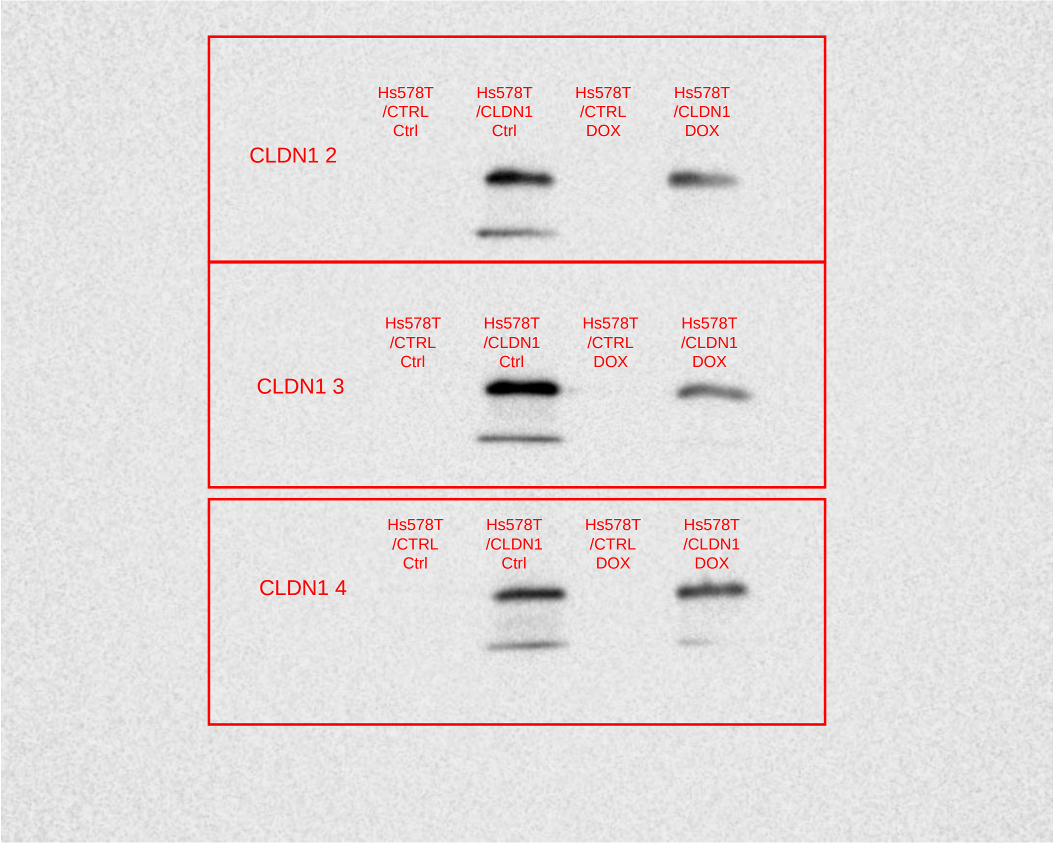

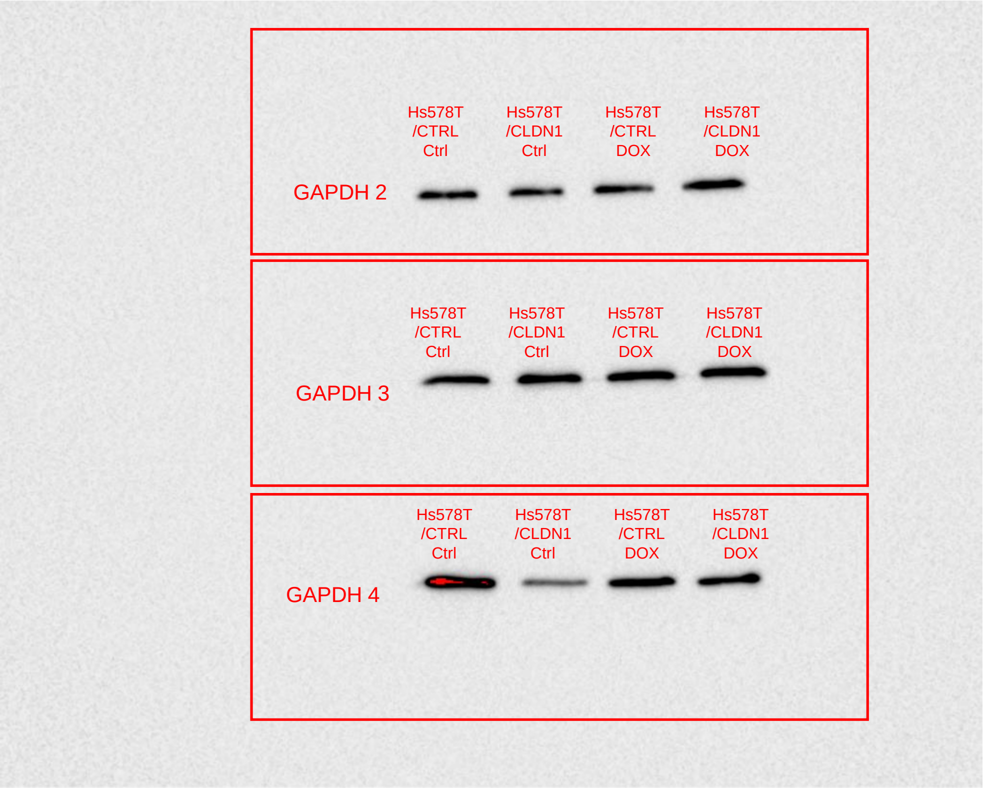

FIGURE 6G n°1, 2 & 3

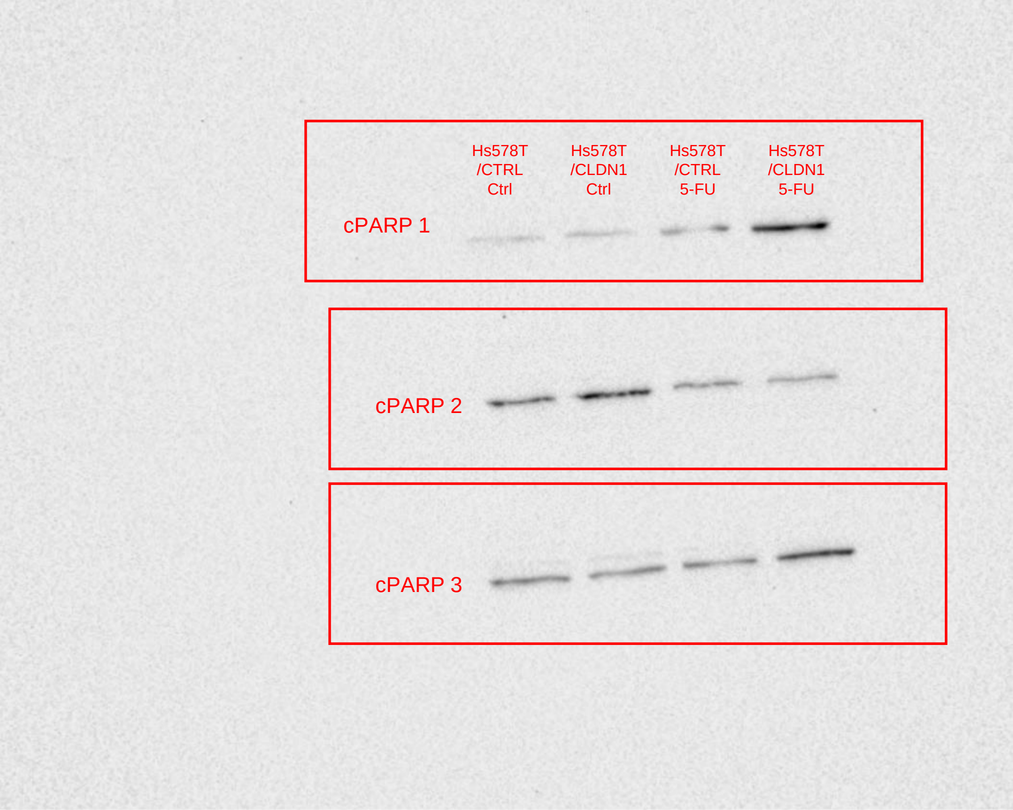

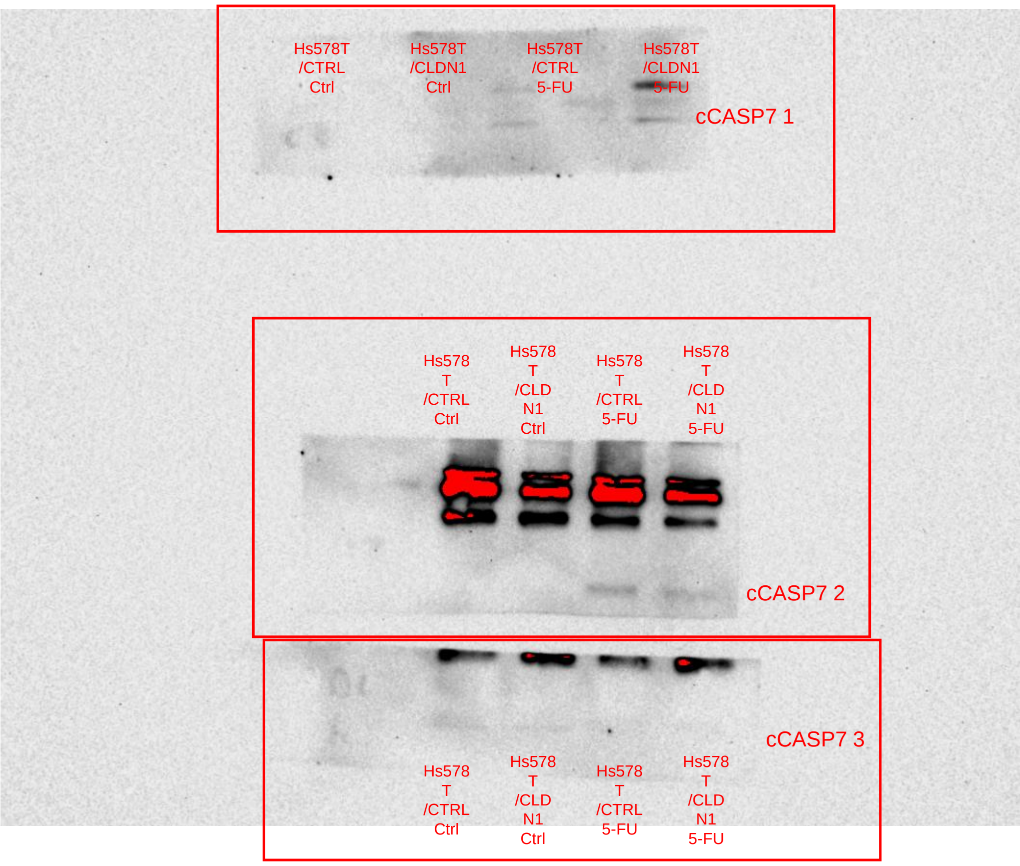

FIGURE 6G n°1, 2 & 3

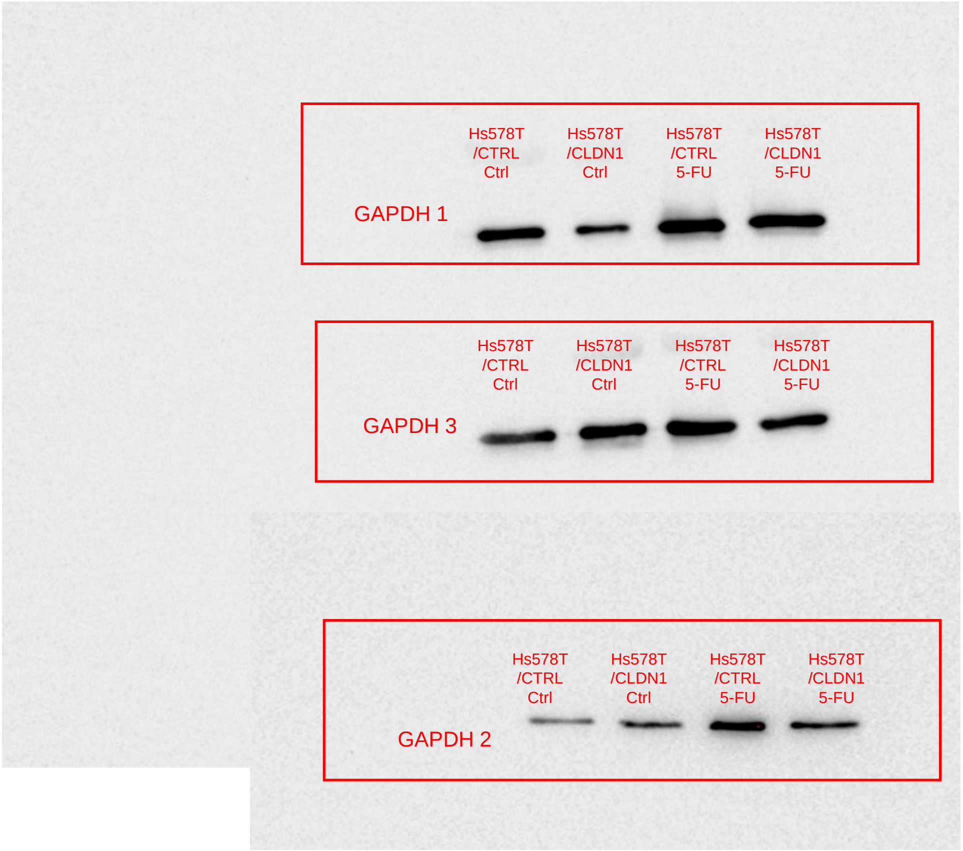

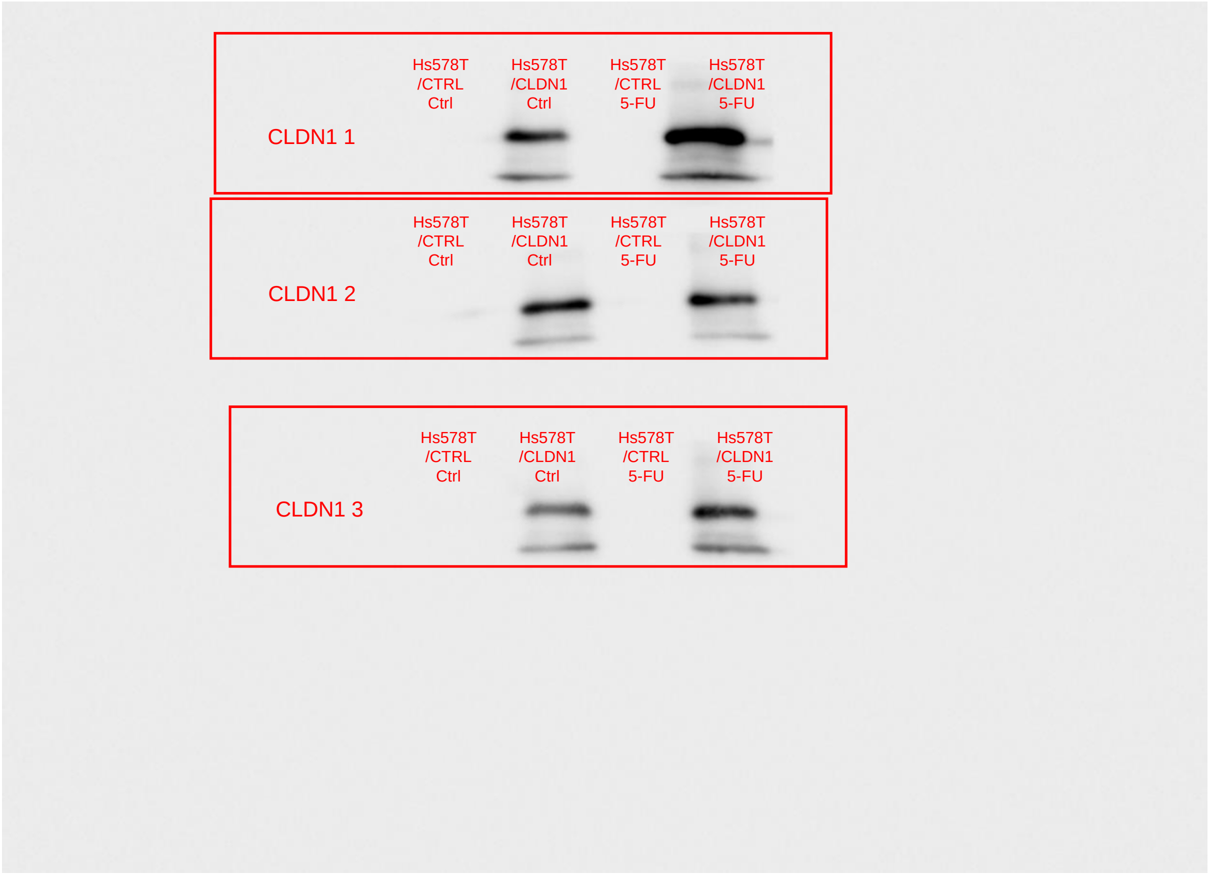

FIGURE 7C n°1 & 2

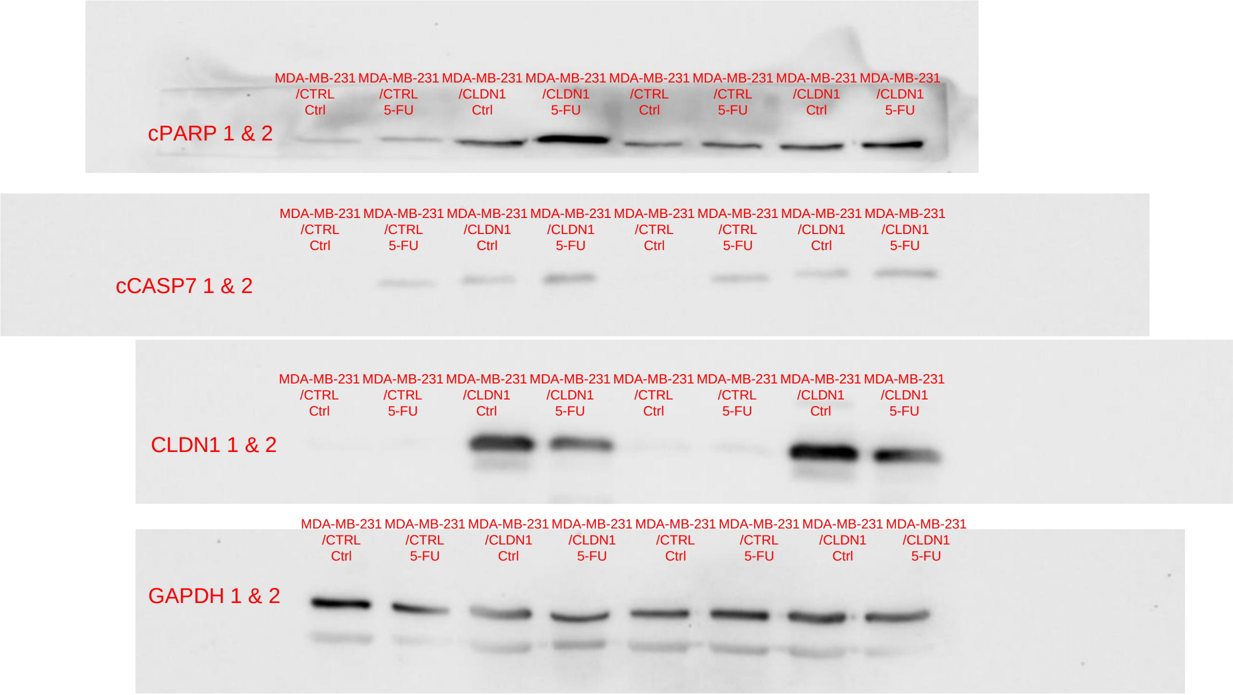

FIGURE 7C n°3 & 4

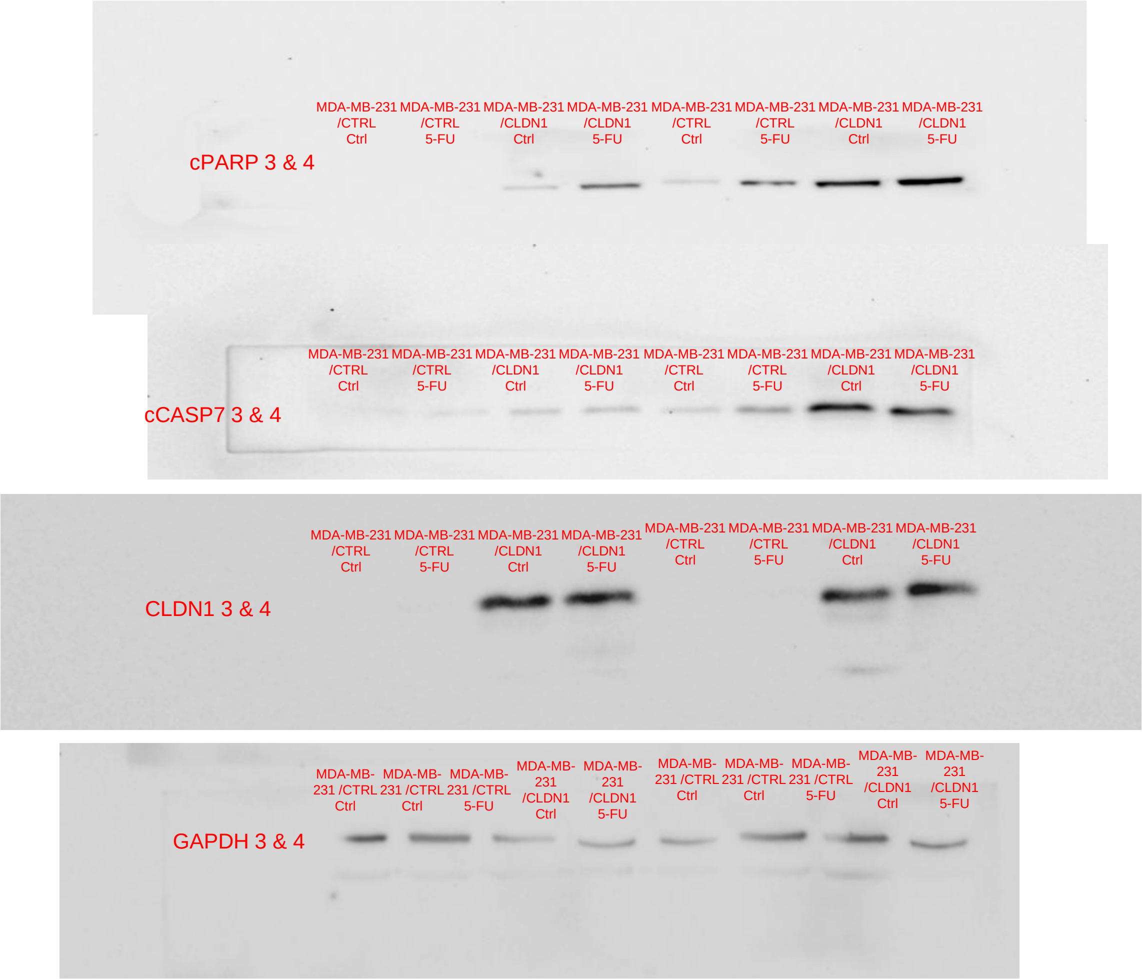

FIGURE 7C n°5

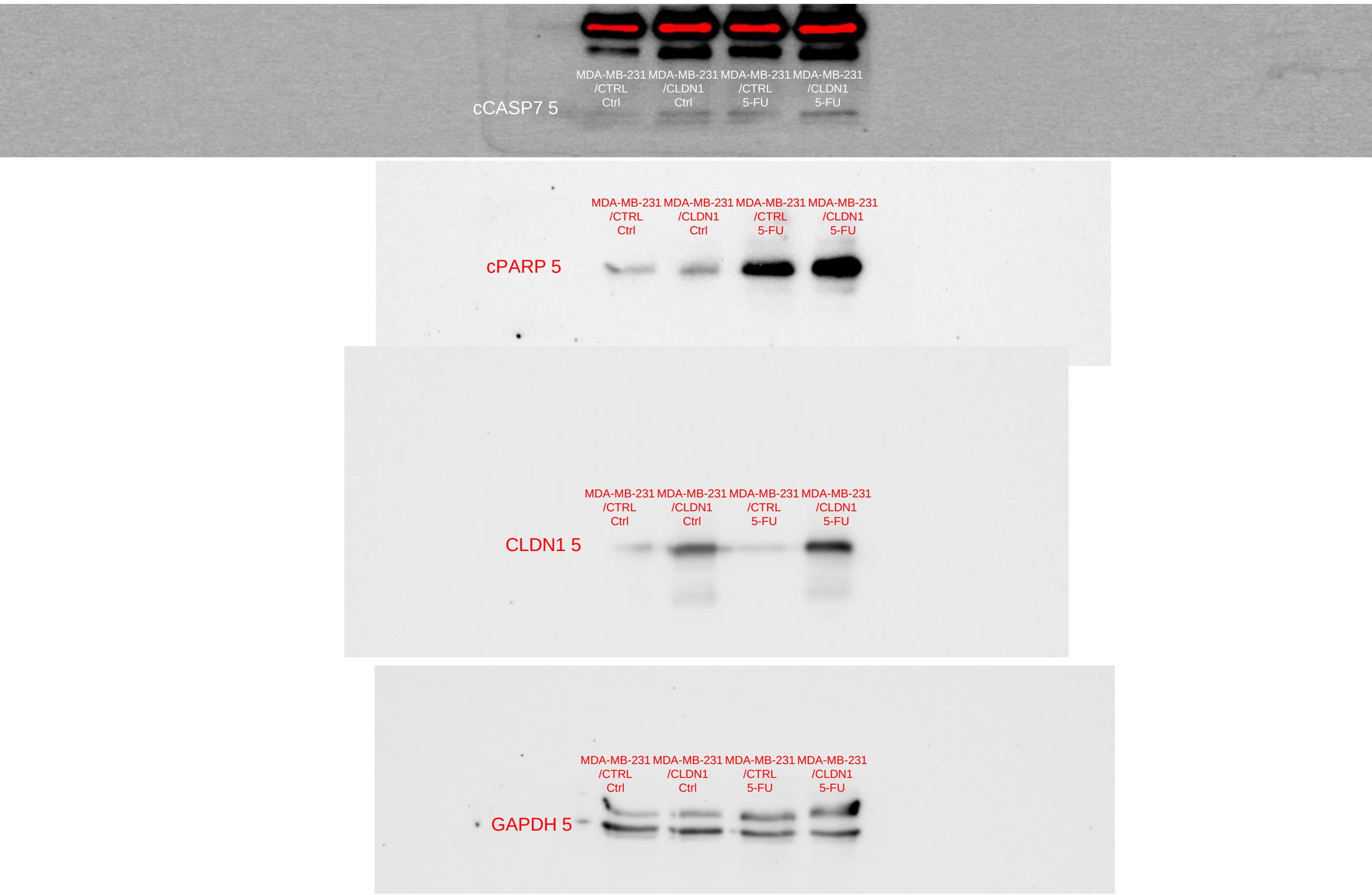
cCASP7 5
MDA-MB-231 /CTRL Ctrl
MDA-MB-231 /CLDN1 Ctrl
MDA-MB-231 /CTRL 5-FU
MDA-MB-231 /CLDN1 5-FU
cPARP 5
MDA-MB-231 /CTRL Ctrl
MDA-MB-231 /CLDN1 Ctrl
MDA-MB-231 /CTRL 5-FU
MDA-MB-231 /CLDN1 5-FU
CLDN1 5
MDA-MB-231 /CTRL Ctrl
MDA-MB-231 /CLDN1 Ctrl
MDA-MB-231 /CTRL 5-FU
MDA-MB-231 /CLDN1 5-FU
GAPDH 5
MDA-MB-231 /CTRL Ctrl
MDA-MB-231 /CLDN1 Ctrl
MDA-MB-231 /CTRL 5-FU
MDA-MB-231 /CLDN1 5-FU

FIGURE 7F n° 1, 2, 3 & 4

* : Other tested condition not included in the article

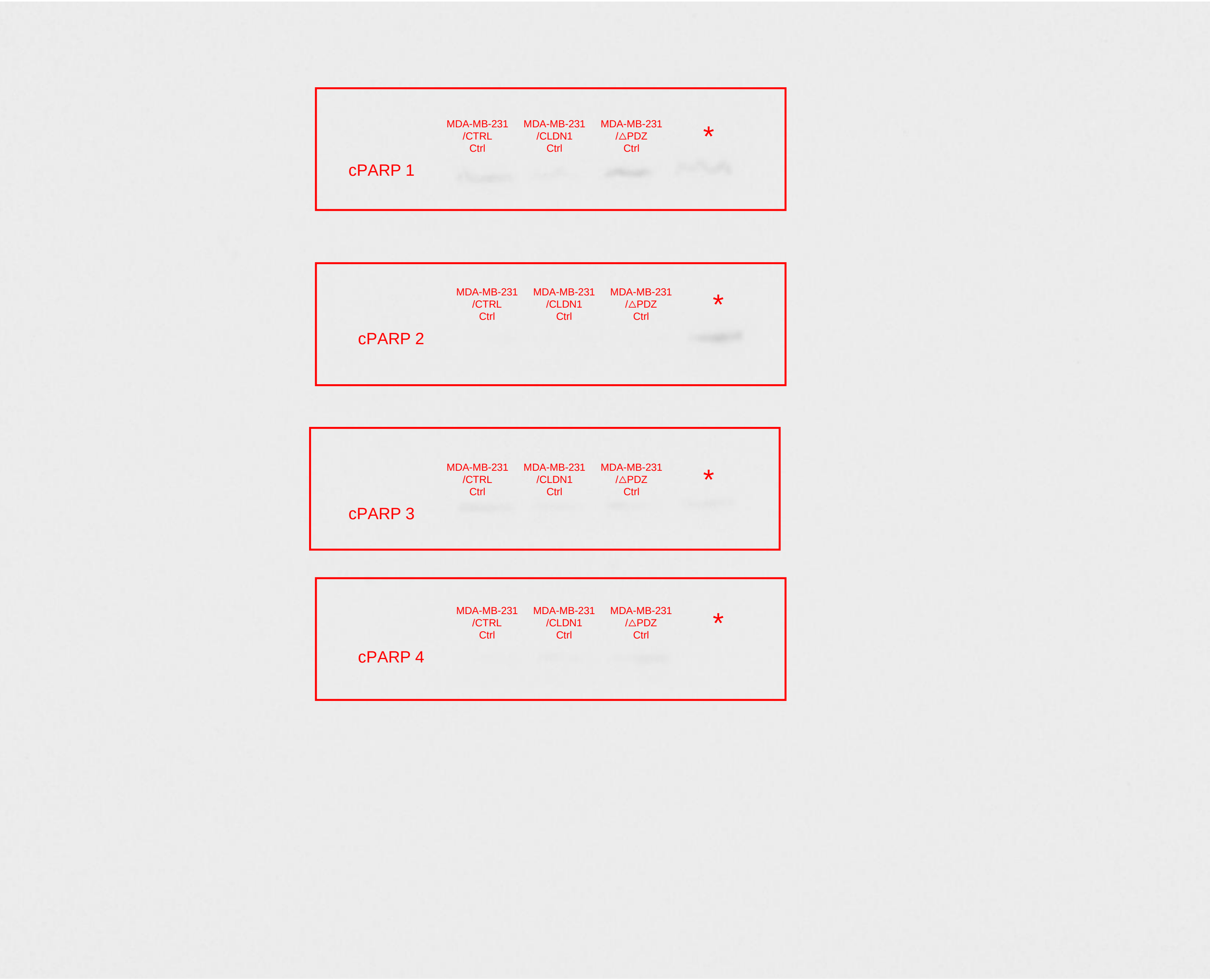

FIGURE 7F n° 1, 2, 3 & 4

* : Other tested condition not included in the article

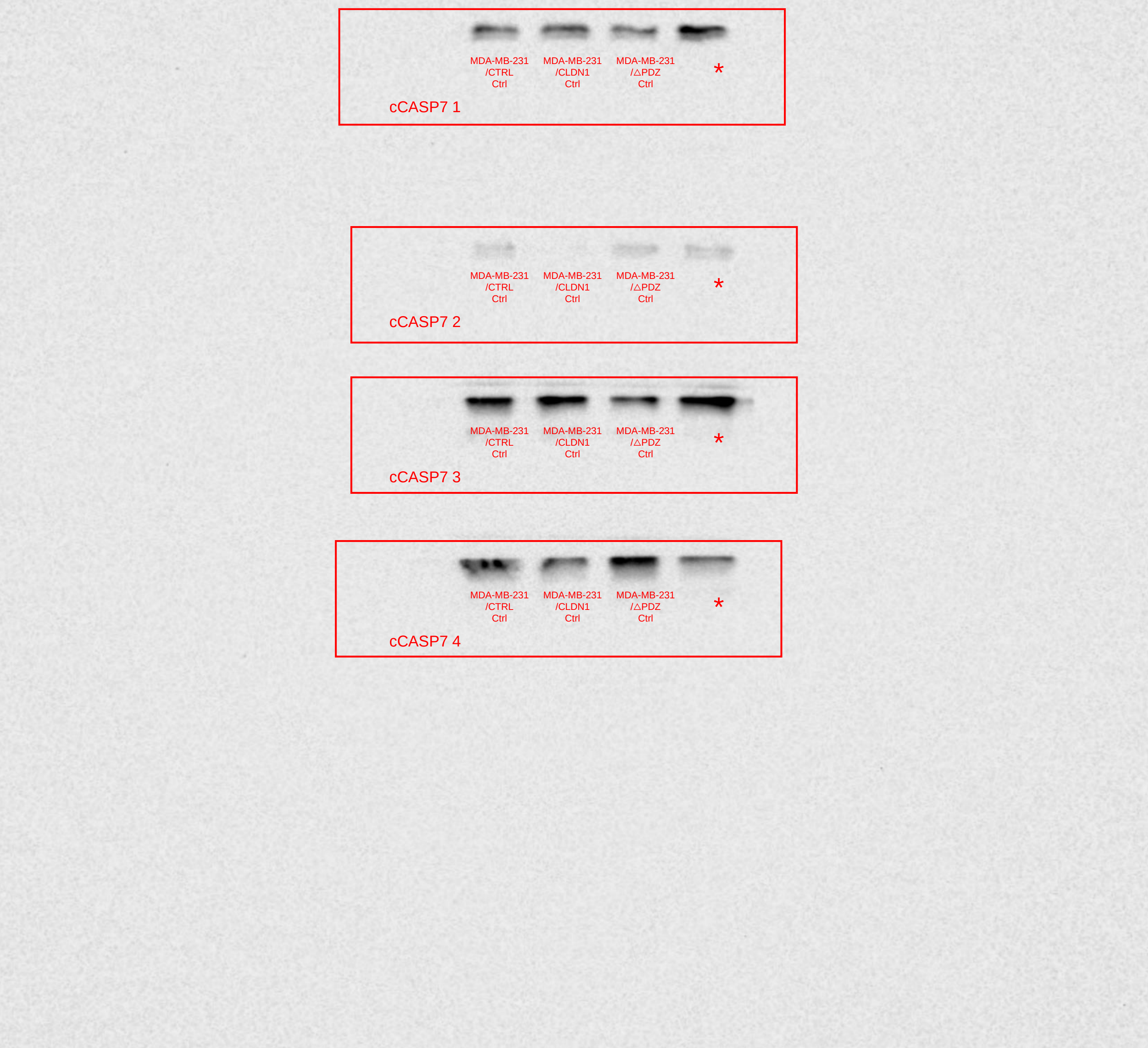

FIGURE 7F n° 1, 2, 3 & 4

* : Other tested condition not included in the article

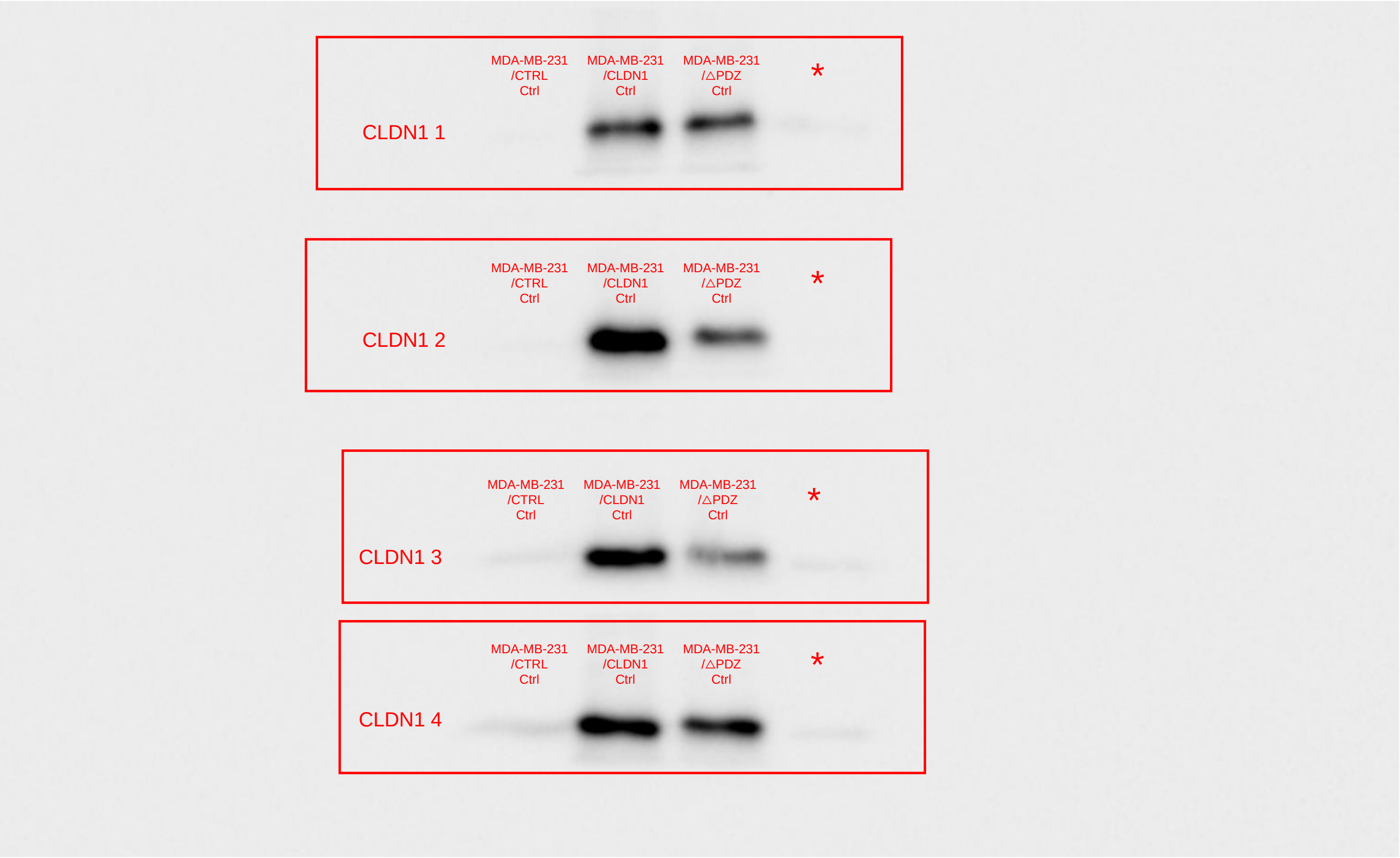

FIGURE 7F n° 1, 2, 3 & 4

* : Other tested condition not included in the article

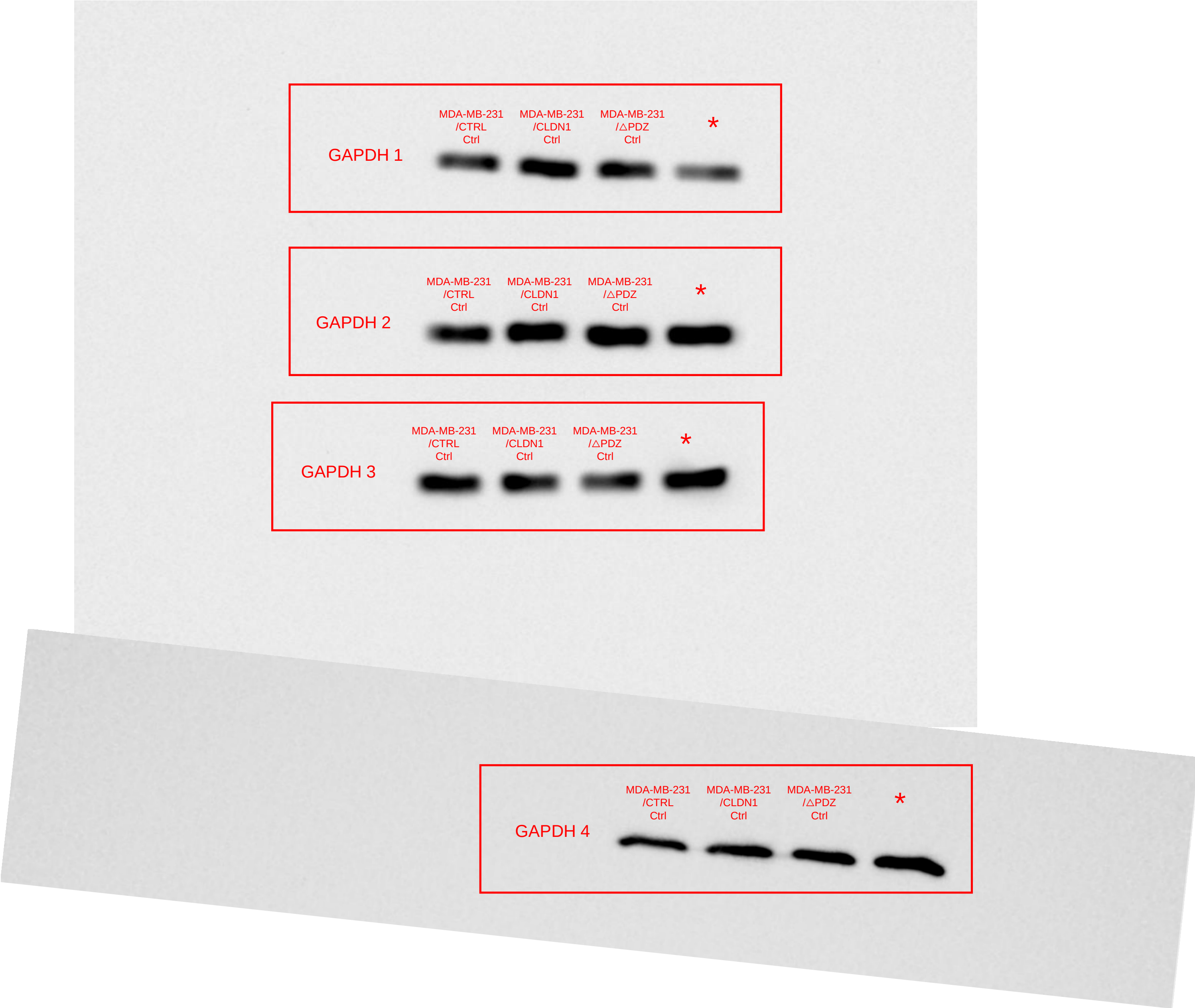

FIGURE 7F n° 1, 2, 3 & 4

* : Other tested condition not included in the article

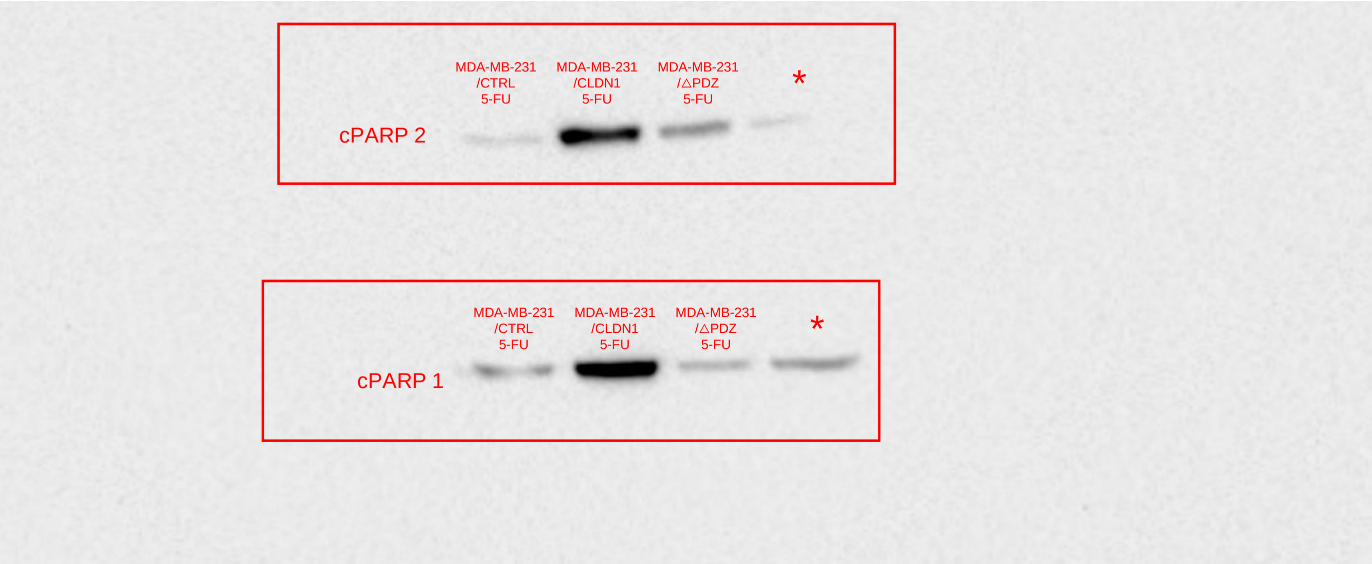

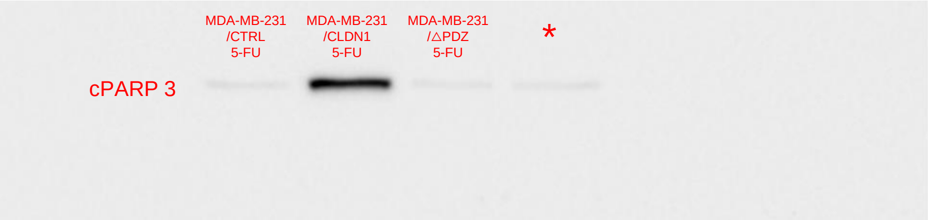

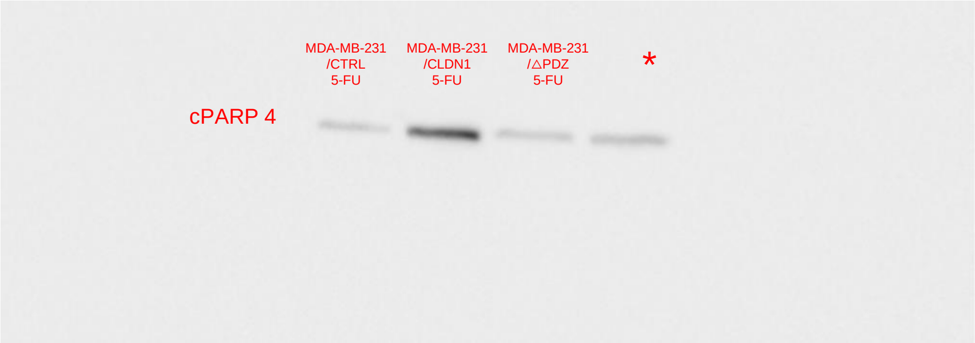

FIGURE 7F n° 1, 2, 3 & 4

* : Other tested condition not included in the article

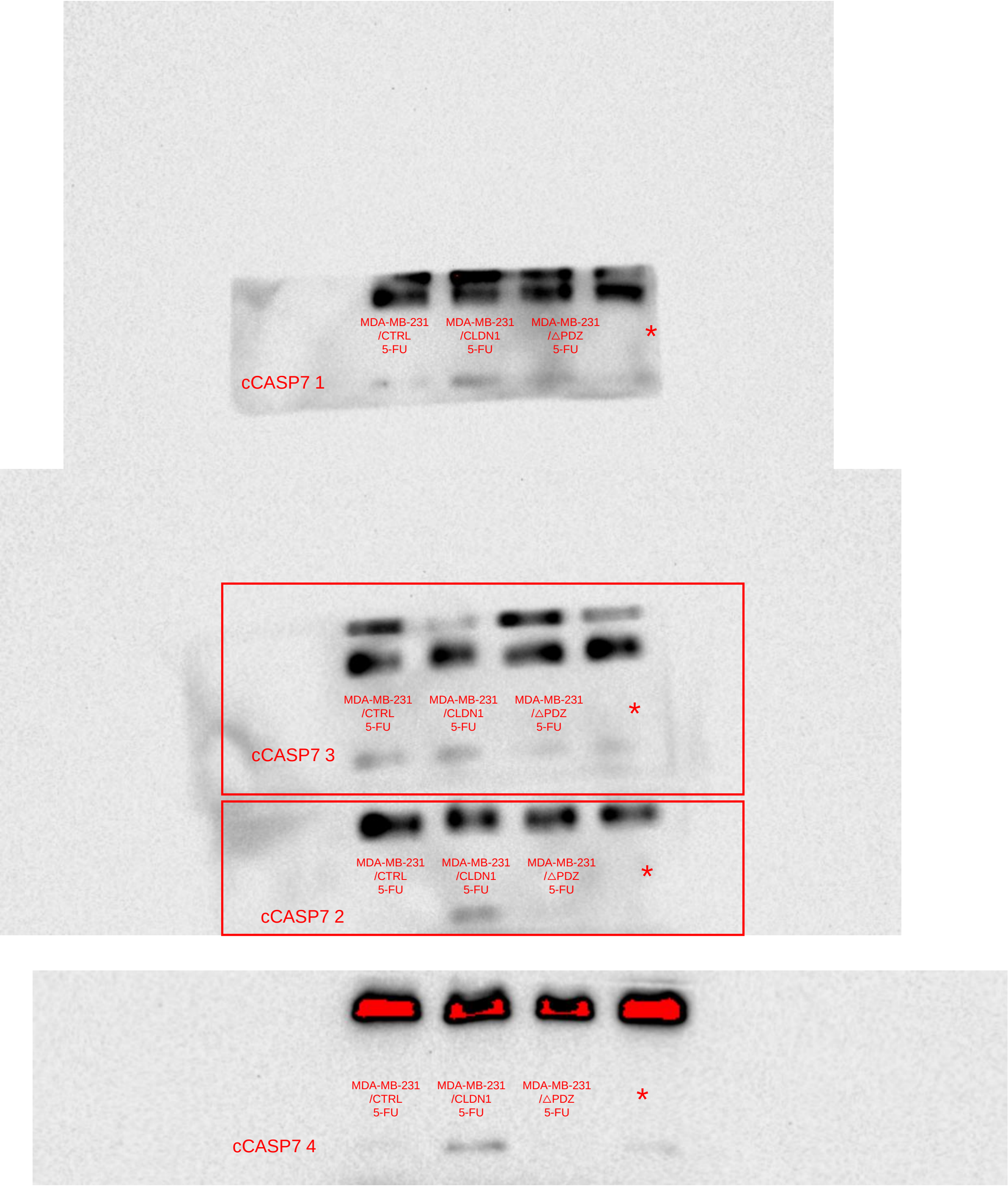

FIGURE 7F n° 1, 2, 3 & 4

* : Other tested condition not included in the article

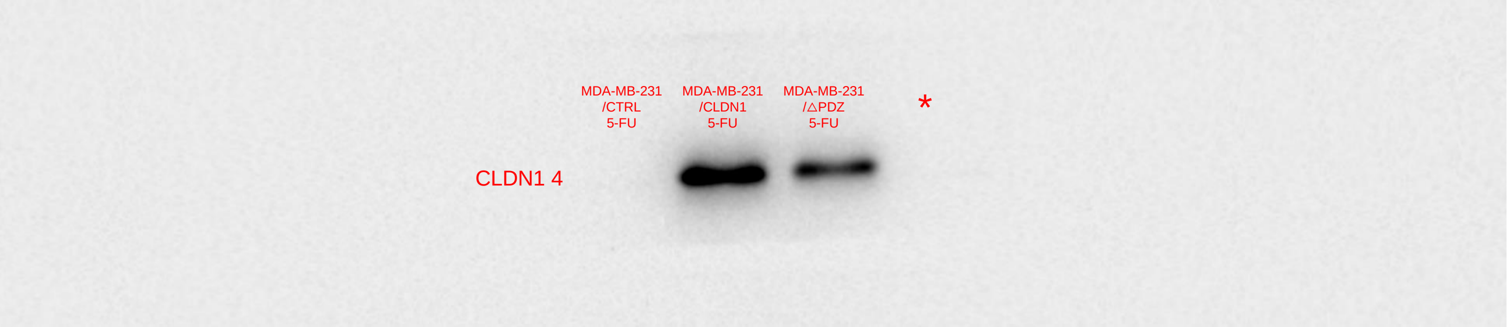

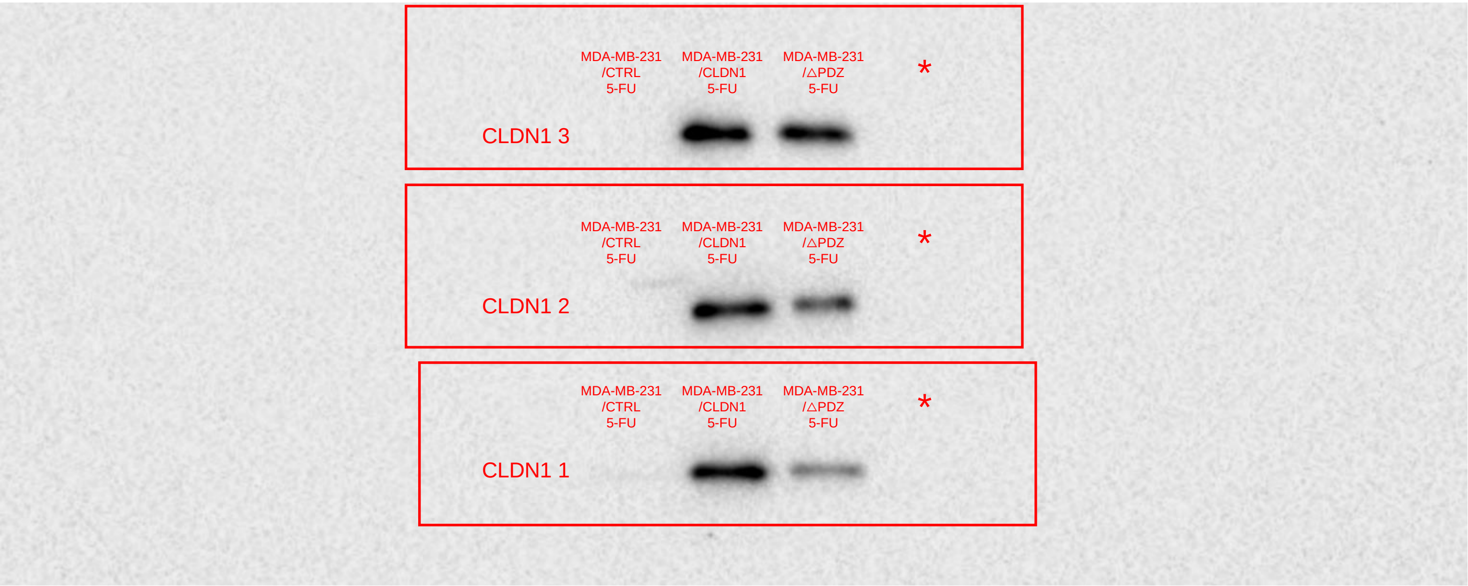

FIGURE 7F n° 1, 2, 3 & 4

* : Other tested condition not included in the article

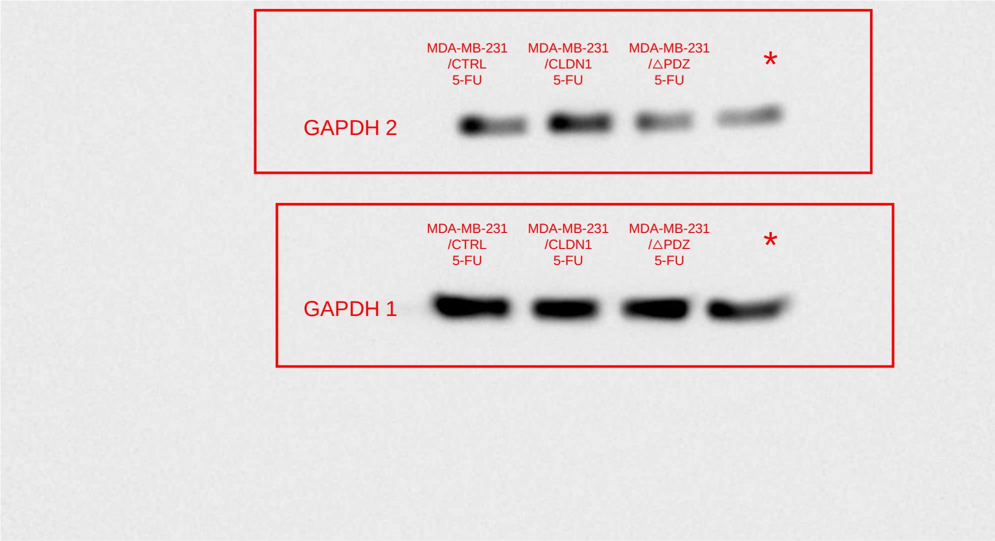

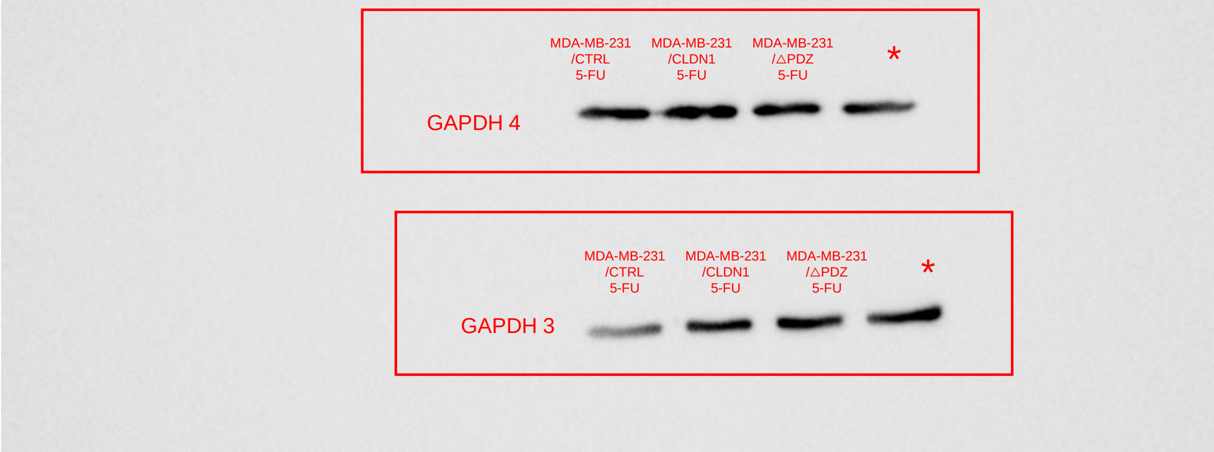

Supplement: Supplementary file 1 [file cancers-14-05026-s001.zip › cancers-1947216-File S1.pdf]
