# Supplementary material for: CLDN1 Sensitizes Triple-Negative Breast Cancer Cells to Chemotherapy
Source: Cancers (Basel). 2022 Oct 14;14(20):5026. doi: 10.3390/cancers14205026 (PMC9599637; doi:10.3390/cancers14205026)
Supplement: Supplementary file 1 [file cancers-14-05026-s001.zip › cancers-1947216-supplementary figures.pdf]

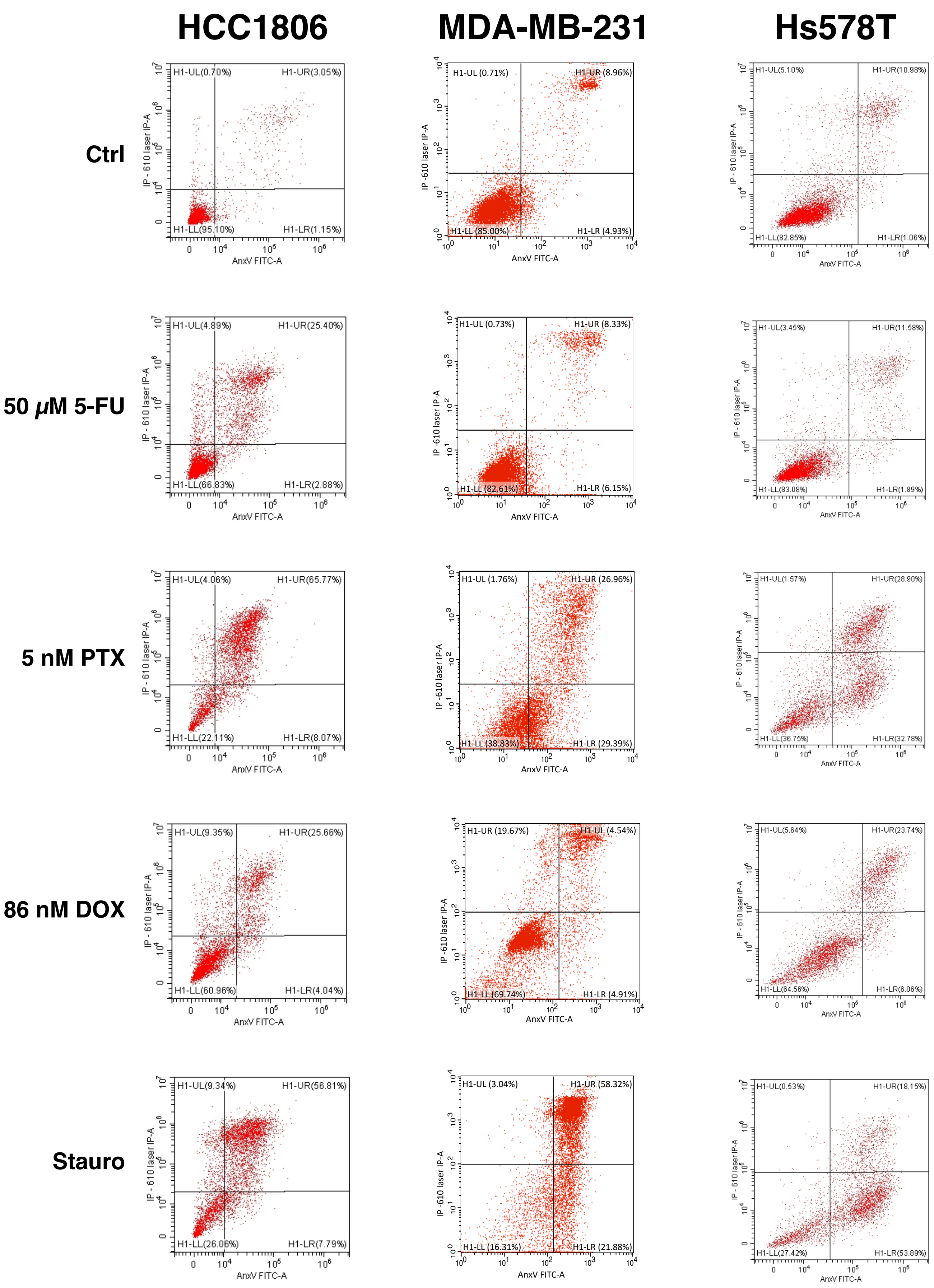

Figure S1. Chemotherapy induced more apoptosis in HCC1806 "claudin-1-high" cells than in "claudin-1-low" cells. HCC1806, MDA-MB-231 and Hs578T cells were treated with 50 µM of 5-FU, 5 nM of PTX and 86 nM of DOX or with solvent (Ctrl) for 72 h. Cells were treated with Staurosporine (Stauro), used as a positive apoptosis induction control, at 50, 500 and 100 nM for each cell line respectively. The cells are co-labeled with annexin V coupled to FITC and with propidium iodide and the living, early- and late-apoptotic cells were analyzed by flow cytometry. The percentage of early-apoptotic cells corresponds to cells labeled with annexin V, cells in late apoptosis or dead cells are co-labeled with annexin V and PI.

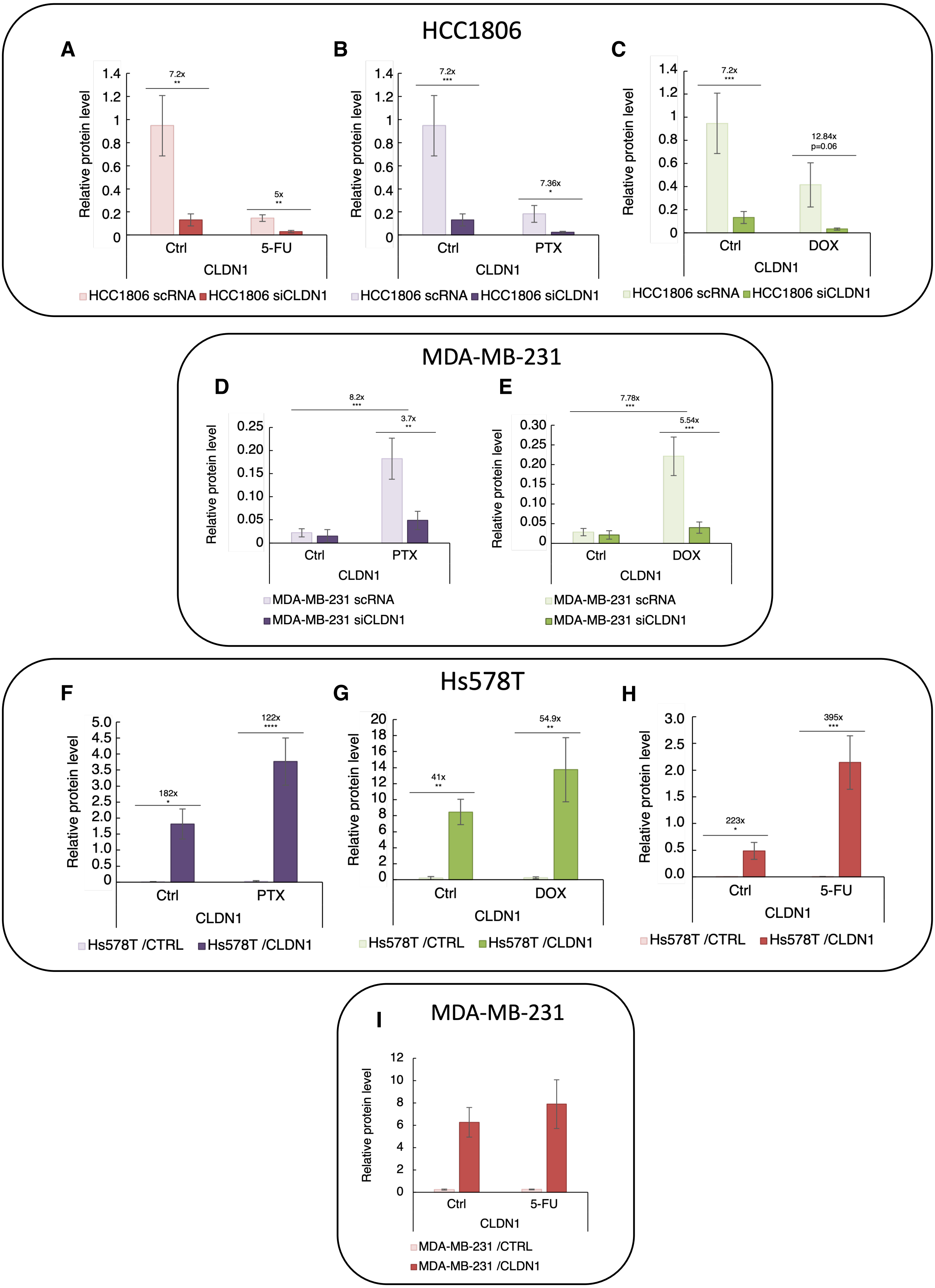

Figure S2. Analyses of CLDN1 expression by Western blot. (**A, B, C**) HCC1806 cells were transfected with a siRNA directed against claudin-1 (HCC 1806 siCLDN1) or with a scramble siRNA (HCC 1806 scRNA) and CLDN1 expression was studied by Western blot. 7 h after transfection, cells were treated for 72 h with (**A**) 50 µM of 5-FU, (**B**) 5 nM of PTX and (**C**) 86 nM of DOX or with solvent (Ctrl). (**D, E**) MDA-MB-231 cells were transfected with a siRNA directed against claudin-1 (MDA-MB-231 siCLDN1) or with a scramble (MDA-MB-231 scRNA). 7 h after transfection, cells were treated for 72 h with (**D**) 5 nM of PTX, (**E**) 86 nM of DOX or with solvent (Ctrl). (**F, G, H**) Hs578T cells stably transfected with CLDN1 vector (Hs578T/CLDN1) or the empty vector (Hs578T/CTRL) were treated for 72 h with (**F**) PTX at 5 nM, (**G**) DOX at 210 nM and (**H**) 5-FU at 181 µM or with solvent (Ctrl). (**I**) MDA-MB-231 cells stably transfected with CLDN1 vector (MDA-MB-231/CLDN1) or the empty vector (MDA-MB-231/CTRL). Relative protein level expressions correspond to CLDN1 on GAPDH ratios. The values represent the means ± SEM of three to eight different experiments. A p-value of less than 0.05 was considered statistically significant with * $p < 0.05$, ** $p < 0.01$, *** $p < 0.001$, **** $p < 0.0001$.

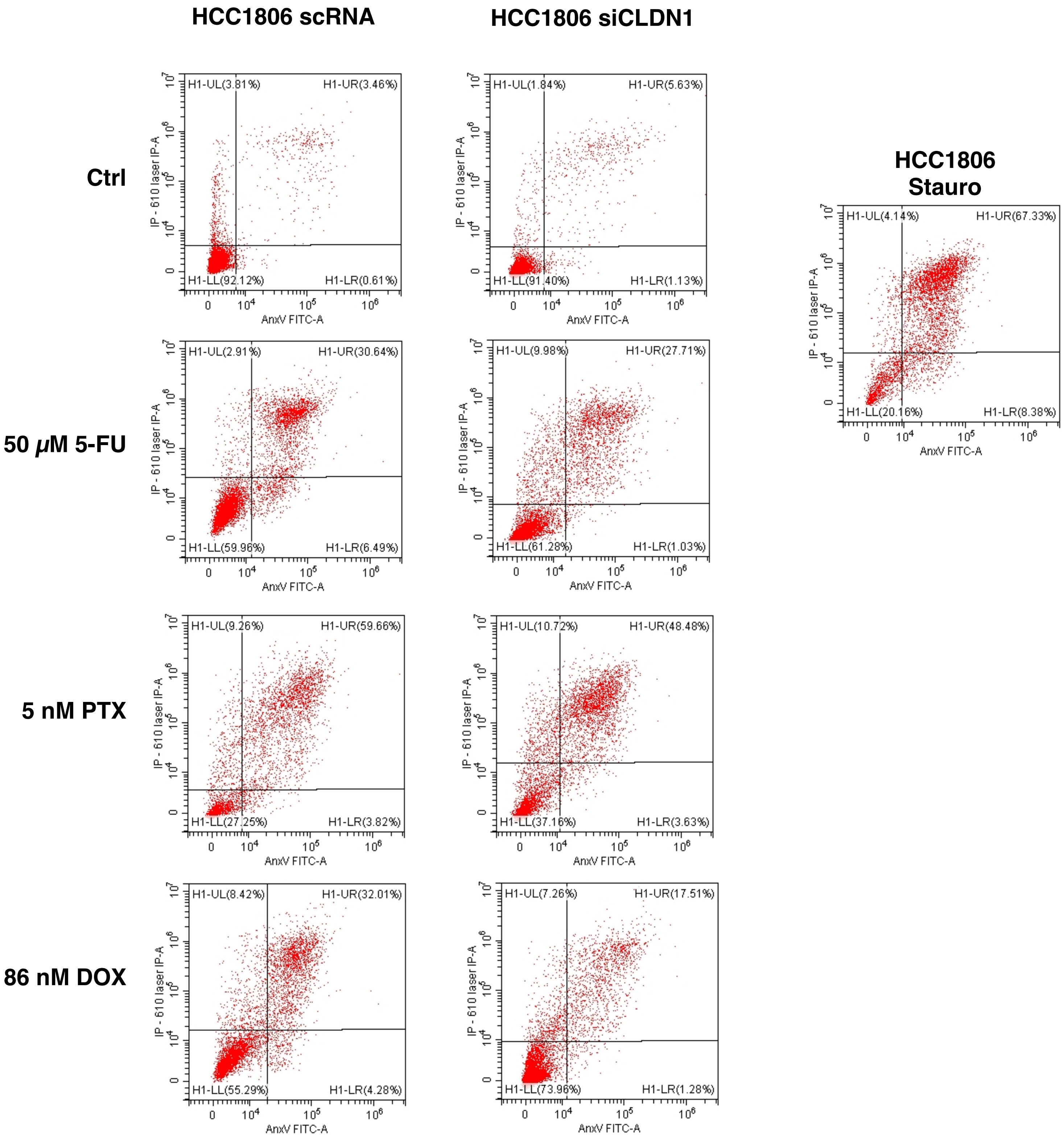

Figure S3. CLDN1 is involved in the chemosensitivity of HCC1806 "claudin-1-high" cells to 5-FU, PTX and DOX. HCC1806 cells were transfected with a siRNA directed against claudin-1 (siCLDN1) or with a scramble siRNA (scRNA) and CLDN1 expression was studied by western blot. 7 h after transfection, cells were treated for 72 h with 50 µM of 5-FU, 5 nM of PTX and 86 nM of DOX or with solvent (Ctrl). Cells were co-labeled with annexin V coupled to FITC and with propidium iodide and the living, early- and late-apoptotic cells were analyzed by flow cytometry. The percentage of early-apoptotic cells corresponds to cells labeled with annexin V, cells in late apoptosis or dead cells are co-labeled with annexin V and PI.

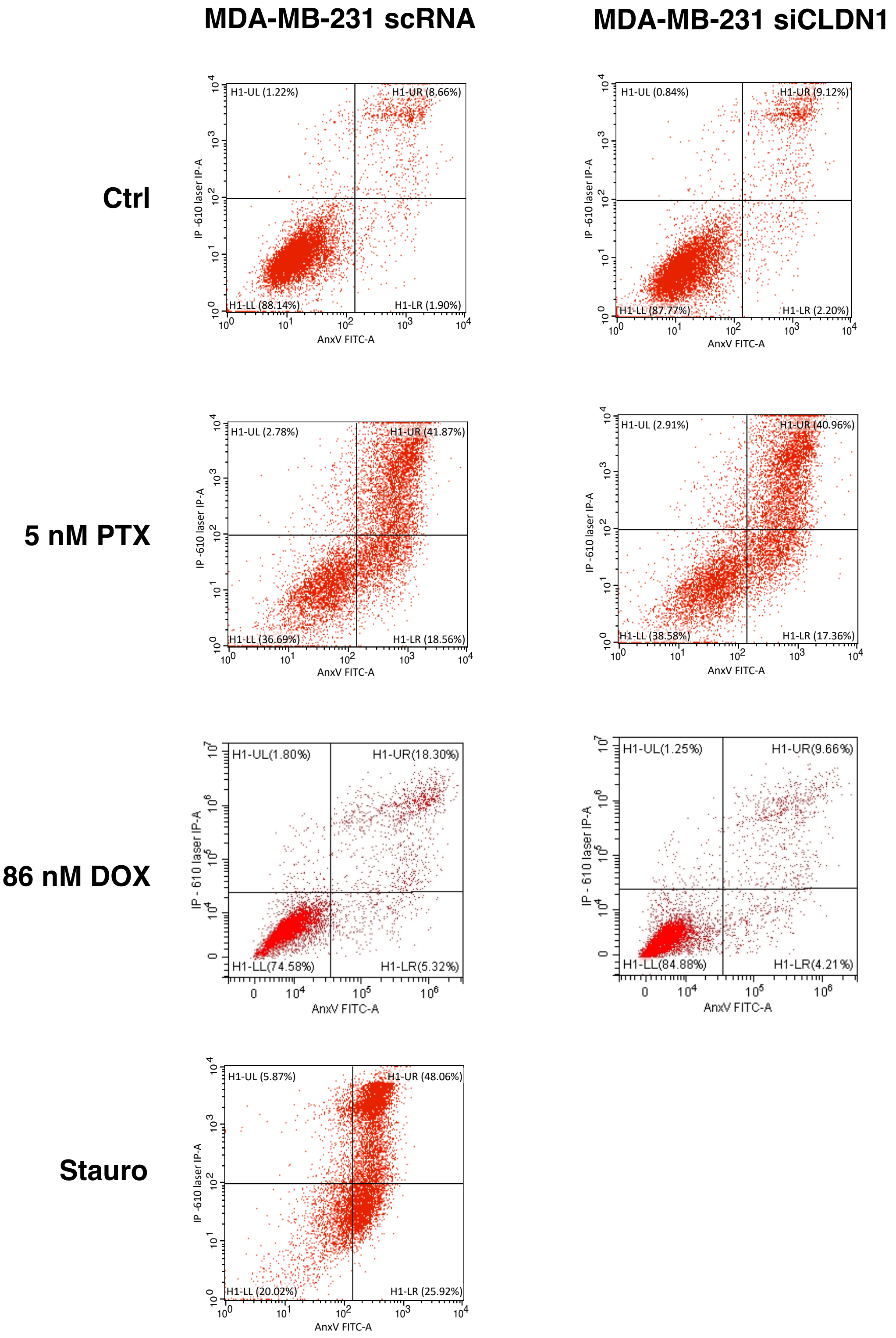

Figure S4. CLDN1 expression is involved in MDA-MB-231 cells sensitivity to PTX and DOX. MDA-MB-231 cells were transfected with a siRNA directed against claudin-1 (siCLDN1) or with a scramble scRNA. 7 h after transfection, cells were treated for 72 h with 5 nM of PTX, 86 nM of DOX or with solvent (Ctrl). MDA-MB-231 cells were co-labeled with annexin V coupled to FITC and with propidium iodide and the living, early- and late-apoptotic cells were analyzed by flow cytometry. The percentage of early-apoptotic cells corresponds to cells labeled with annexin V, cells in late apoptosis or dead cells are co-labeled with annexin V and PI.

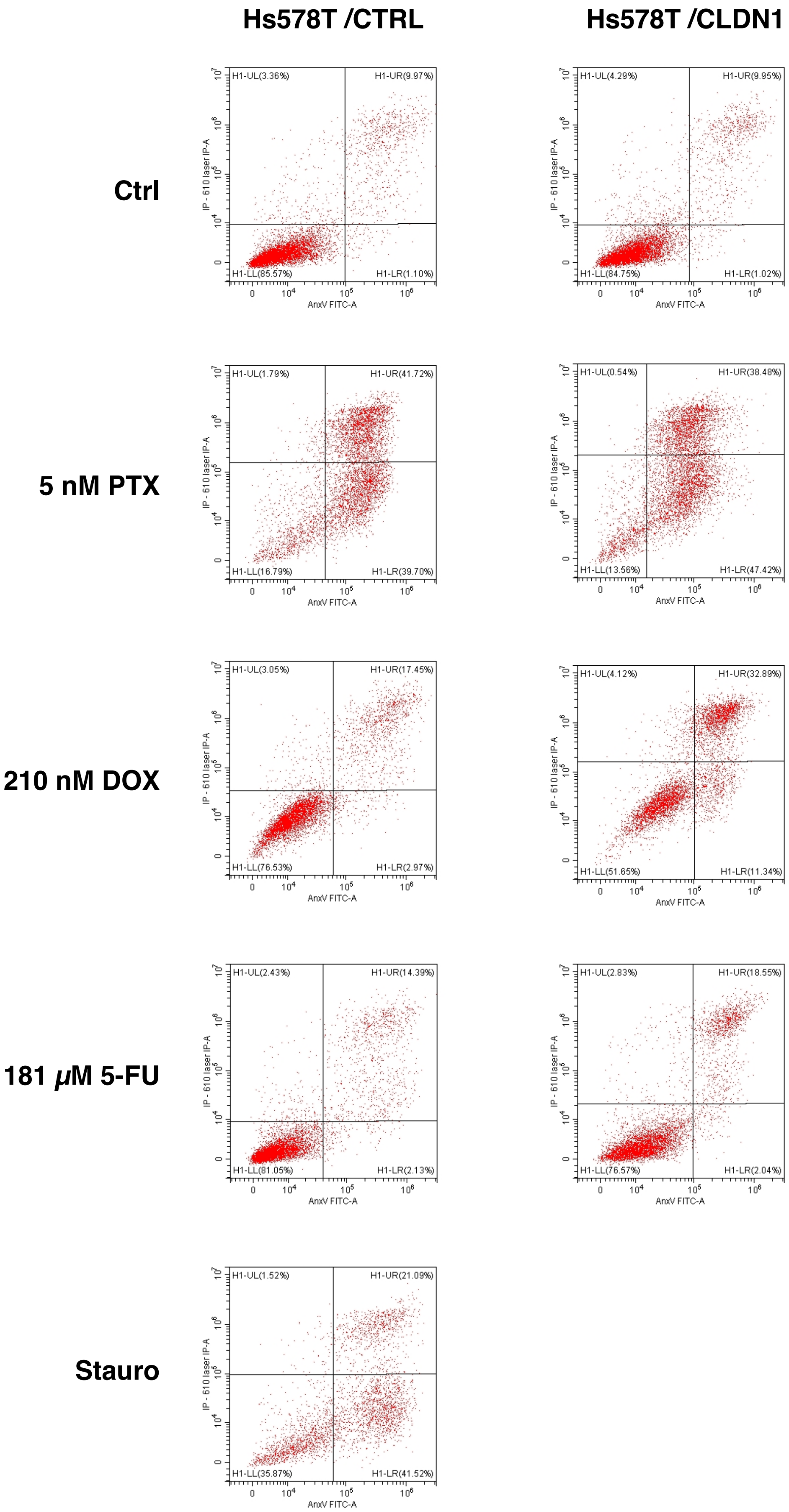

Figure S5. CLDN1 overexpression sensitizes Hs578T cells to PTX and DOX but not to 5-FU. Hs578T cells stably transfected with CLDN1 vector (Hs578T /CLDN1) or the empty vector (Hs578T /CTRL) were treated for 72 h with PTX at 5 nM, DOX at 210 nM and 5-FU at 181 μM, or with solvent (Ctrl). Hs578T cells were co-labeled with annexin V coupled to FITC and with propidium iodide and the living, early- and late-apoptotic cells were analyzed by flow cytometry. The percentage of early-apoptotic cells corresponds to cells labeled with annexin V, cells in late apoptosis or dead cells are co-labeled with annexin V and PI.

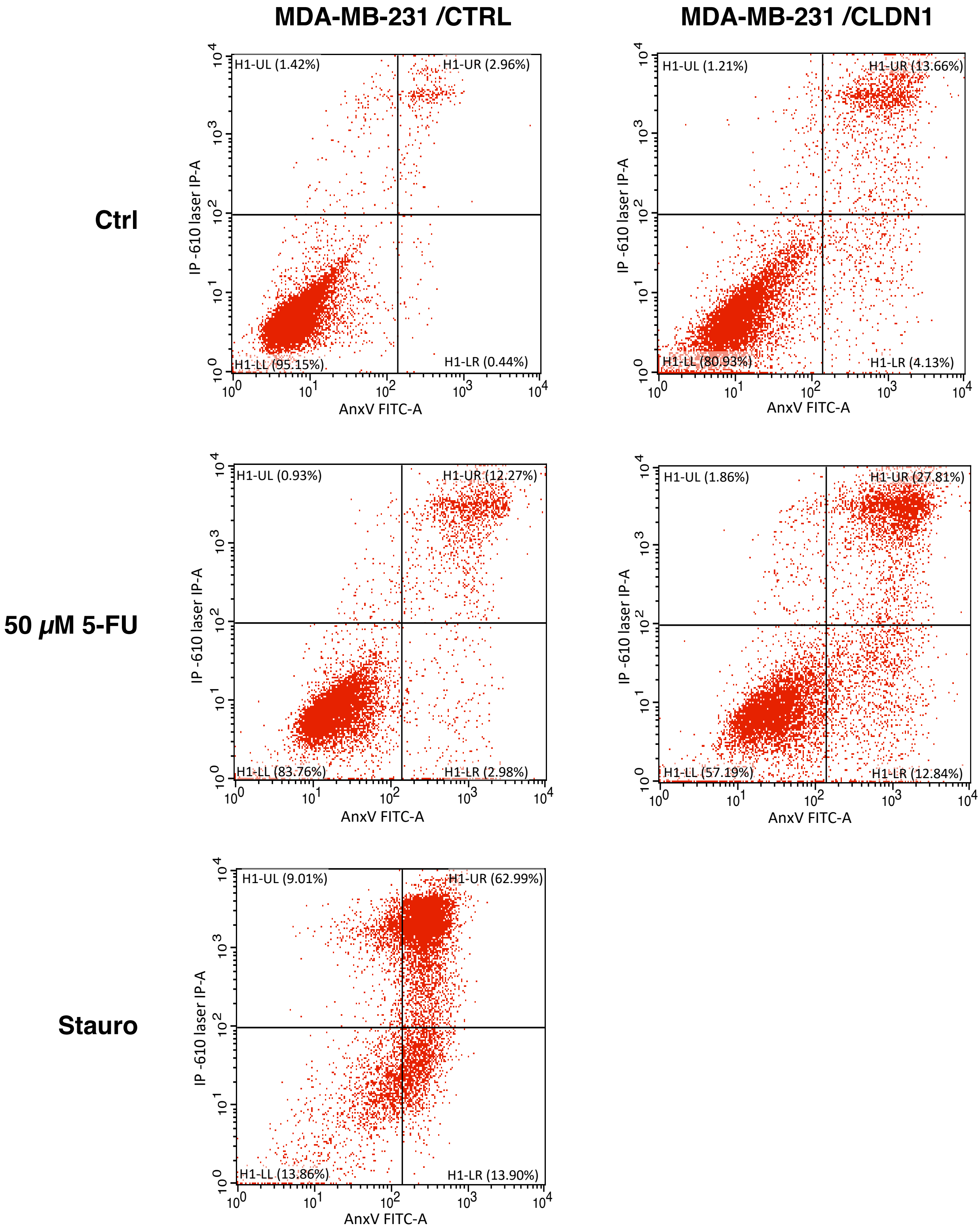

Figure S6. CLDN1 expression sensitizes MDA-MB-231 cells to 5-FU. MDA-MB-231 cells stably transfected with CLDN1 vector (MDA-MB-231 /CLDN1) or the empty vector (MDA-MB-231 /CTRL) were treated for 72 h with 5-FU at 50 µM or with solvent (Ctrl). Cells were co-labeled with annexin V coupled to FITC and with propidium iodide and the living, early- and late-apoptotic cells were analyzed by flow cytometry. The percentage of early-apoptotic cells corresponds to cells labeled with annexin V, cells in late apoptosis or dead cells are co-labeled with annexin V and PI.
